# A computational approach for modeling electronic circular dichroism of solvated chromophores

**DOI:** 10.1002/jcc.27001

**Published:** 2022-09-22

**Authors:** Marta Monti, Mauro Stener, Massimiliano Aschi

**Affiliations:** ^1^ Dipartimento di Scienze Chimiche e Farmaceutiche Università di Trieste Trieste Italy; ^2^ Dipartimento di Scienze Fisiche e Chimiche Università dell'Aquila L'Aquila Italy

**Keywords:** CD spectroscopy, DFT, essential dynamics, molecular dynamics, TDDFT

## Abstract

The present study consists in a novel computational protocol to model the UV‐circular dichroism spectra of solvated species. It makes use of quantum‐chemical calculations on a series of conformations of a flexible chromophore or on a series of chromophore/solvent clusters extracted from molecular dynamic simulations. The protocol is described and applied to the aqueous cationic tripeptide GAG^+^ and to the aqueous neutral decapeptide (GVGVP)_2_. The protocol has proven able to: (i) properly consider the conformational motion of solute in the given environment; (ii) give the actual statistical weight of each conformational state; (iii) provide a reliable quantum mechanical method able to reproduce the spectral features. Temperature effects on conformations and spectral properties are properly taken into account. The role of explicit solvent on the conformational analysis and the spectra calculation is discussed. The comparison of the calculated circular dichroism spectra with experimental ones recorded at different temperatures represents a strict validation test of the method.

## Graphical abstract

A new computational protocol to model the UV‐circular dichroism spectra of solvated species is presented. It makes use of quantum‐chemical calculations on a series of conformations of a flexible chromophore or on a series of chromophore/solvent clusters extracted from molecular dynamic simulations. The protocol is described and applied to the aqueous cationic tripeptide GAG^+^ and to the aqueous neutral decapeptide (GVGVP)_2_. Temperature effects on conformations and spectral properties are properly taken into account. The role of explicit solvent on the conformational analysis and the spectra calculation is discussed.

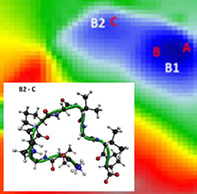

## 1 INTRODUCTION

The theoretical modeling of electronic circular dichroism (ECD) has attracted much interest in the last decades, due to its usefulness to ascribe the structure to absolute enantiomers of chiral compounds.^1^ Indeed, if a reliable protocol would be available to calculate the ECD, a direct comparison of the calculated ECD with the experimental one would allow to easily ascribe the absolute structure of the enantiomer. The possibility to employ such a strategy in practice depends on the reliability of the computational protocol. At the moment, ECD spectra can be calculated with enough accuracy by means of the Time Dependent Density Functional Theory^2^ employing accurate exchange‐correlation functionals, typically hybrid ones such as B3LYP^3^, ^4^ or more elaborate ones, for instance including Range Separated functionals such as CAM‐B3LYP.^5^ Such a scheme is generally adequate for the treatment of the electronic problem in organic compounds. However, it must be considered that the ECD is extremely sensitive to the conformations of the system under study,^1^ and that the conformations themselves are further sensitive to the presence of the solvent, a situation which is further amplified when hydrogen‐bonds are present such as in polypeptides. For this reason, a valid computational protocol for the calculation of the ECD not only needs an accurate electronic scheme, but also requires a physically coherent inclusion of the contributions of several conformations as well as of the solvent effects, at least at a partial extent.^6^, ^7^

Therefore, many scientists have worked on this topic and the scientific literature in the field is quite wide. Grimme et al. suggested a simplified TDDFT (sTDDFT), which is so cheap to allow many calculations on several snapshots taken from a preliminary molecular dynamics (MD)^8^ calculation, or an automated procedure to treat flexible molecules.^9^ From a different perspective, Caricato et al. have studied the problem of the gauge choice in optical rotation.^10^, ^11^, ^12^, ^13^, ^14^ Mennucci et al. worked on excitonic models^15^ while Cappelli on the chiroptical properties in aqueous solution.^16^ Chiroptical properties in solution have been treated also by Barone et al.,^17^ who recently suggested an automated procedure based on evolutionary algorithm.^18^ Autschbach et al. wrote a recent review on optical activity.^19^ Crawford has studied the effect of the basis set choice on optical rotation^20^ and included solvent effects in a combined MD + DFT approach.^21^ Finally, Ruud extended the methods for optical activity to the relativistic case.^22^, ^23^

Most of the above methods treat the contributions of the different conformers of the chromophore by a preliminary MD search, which allows to properly span the associated conformational repertoire. Subsequently the ECD spectra are calculated, a posteriori, for all conformers and properly weighted in order to obtain a Boltzmann averaged spectrum, which can be directly compared with the experiment. Such a procedure is in principle very accurate provided four basic mutually interconnected conditions are fulfilled: first, a sufficiently accurate MD simulation can be carried out for an adequately long time, typically from dozens to several hundreds of nanoseconds depending on the size and conformational flexibility of the chromophore of interest; second, the chromophore itself can be safely defined; third, an exhaustive conformational analysis can be performed and, finally, a valid but still affordable quantum‐chemical approach is available. Concerning the first argument, the reliability of the MD simulation—or more in general of whatever tool for the semi‐classical span of a conformational space—obviously depends on the accuracy of the force‐field. On the other hand, concerning the second argument, when considering chromophores in strong interaction with the solvent (e.g., through H‐bond), it might be necessary to include explicit solvent molecules in the definition of the chromophore itself. It follows that, in any case, the geometrical identification and the subsequent extraction from the MD simulation of the relevant chromophore conformations (the third argument), irrespectively from its definition, might not represent a straightforward task. As a matter of the fact if we exclude the trivial case of rigid chromophores not strongly interacting with the solvent or the less trivial, although still affordable, cases of chromophores whose conformational transitions can be easily followed by a reduced set of internal coordinates (e.g., the Ramachandran plot, see for example Reference 24), the unbiased conformational analysis might represent the actual bottleneck of the whole procedure. For this purpose, the essential dynamics (ED)^25^, ^26^ analysis could represent a powerful tool for obtaining a reduced, and hence computationally affordable, number of internal degrees of freedom. ED, which consists in the construction of the positional covariance matrix (CM) of a chromophore—excluding the roto‐translations—of arbitrary size, is usually employed to describe the fluctuations of a covalent framework (i.e., a single flexible chromophore), however recently it has been also extended for addressing the conformational repertoire of weakly interacting systems such as the previously mentioned clusters of solvated chromophores.^27^ In the present work, we provide a computational protocol for the calculation of ECD spectra of solvated species based on quantum‐chemical calculations carried out on a number of conformations extracted through MD‐ED analysis and also taking into account the possible effects of the inclusion of the explicit solvation shells in interaction with the chromophore. Note that with “conformations” in this study we identify well‐defined spatial organizations not only of the chromophore but also of the cluster formed by the chromophore and a predefined number of solvent molecules. For this purpose, we decided to use, as test cases, the aqueous cationic tripeptide (GAG^+^) and the aqueous neutral decapeptide (GVGVP)_2_ (see Figure S1) whose experimental ECD data^28^, ^29^, ^30^, ^31^ are available also at temperature values different from the room temperature. Most importantly, reliable force‐fields are available for these systems and, moreover their conformational features have been extensively investigated in the past.

The possibility to compare the calculated and the experimental ECD spectra also at different physical conditions certainly represents a stringent test for the presented computational protocol. The work is organized as follows: first, an outline of the employed theoretical methods is given (MD, ED, DFT, and TDDFT), then the results are presented according to a logical ordering starting from MD results, ED analysis, and ECD spectra for both systems. This part is repeated twice with and without explicit solvent molecules, in order to assess with more confidence the importance of the solvent effects. Finally, a direct comparison with the available experimental data is performed, in order to discuss the temperature effect and to identify all the possible drawbacks and source of errors.

## 2 COMPUTATIONAL DETAILS

### 2.1 Molecular dynamics

All the molecular dynamics (MD) simulations were carried out using the Gromacs package,^32^ version 5.1.2. The peptides (either cationic GAG or GVGVPGVGVP) were described using the OPLS‐AA force field.^33^, ^34^ For water solvent, the single point charge (spc) model was adopted.^35^ A proper number of counterions (chloride ions) was also added for ensuring the electroneutrality of the box. It is important to remark that the choice of the force‐field is essential for the final outcome. However, in this first study, we did not take into account the possible effects induced by the use different force fields on the quality of the final spectra.

The temperature was kept constant using the velocity rescaling model^36^ and the LINCS algorithm^37^ was employed to constrain all the bond lengths. Long range electrostatic interactions were computed by the Particle Mesh Ewald method^38^ with 34 wave vectors in each dimension and a 4^th^ order cubic interpolation and a cut‐off of 1.0 nm was used. All the simulations were carried out in the NVT ensemble. The solute (either GAG^+^ or (GVGVP)_2_) was inserted in a cubic box preventively adjusted to correctly reproduce the correct density, in infinite dilution in water, at the temperature of interest and 1.0 bar of pressure. This latter condition has been achieved by modifying the solute‐solvent box in order to reach the same average pressure of a box of pure water (i.e., with the same number of spc particles corresponding to 600 and 800 for GAG^+^ and (GVGVP)_2_, respectively) preventively simulated in the NVT ensemble at the experimental density of the pure water at the temperature of interest. In this respect, for the pure water simulations, we utilized the following densities at the corresponding temperatures: 999.06 g/L (15°C), 995.61 g/L (30°C), 968.59 g/L (85°C), 965.30 g/L (90°C). The solvated peptide simulations (hereafter free‐MD), after the initial mechanical and thermal equilibration disregarded from the analysis, were propagated for 40 and 100 ns for GAG^+^ and (GVGVP)_2_, respectively. Additional simulations (termed as constrained‐MD, see below) were subsequently carried out upon keeping frozen the peptide in each of the different conformations previously extracted from the free‐MD as described in the next paragraph.

### 2.2 Essential dynamics

The conformational repertoire of the chromophore (irrespectively of its definition, see above) was accomplished by analyzing the MD simulations using ED whose basic features are widely described in the literature^25^, ^26^ and here only briefly outlined. In the case of the free‐MD, the peptide positional CM is first constructed using, in the present case, the backbone atoms and then diagonalized. This operation produces a set of eigenvectors, that is, a set of eigendirections along which the peptide undergoes the internal fluctuations, with the associated eigenvalues corresponding to the actual value of the mean square fluctuations. Therefore, the eigenvectors characterized by the highest eigenvalues represent the eigendirections describing the fluctuations with largest amplitude necessary, that is, essential, to account for most of the peptide conformational transitions. If the number of these eigenvectors, hereafter termed as Essential Eigenvectors, is limited to one or two, the corresponding projection of the peptide trajectory on these eigenvectors, that is, the Principal Components, provides us with a conformational landscape (with respect to a single coordinate or to a plane) relatively easy to visualize and to analyze. In this respect, the regions of the conformational landscape more frequently spanned by the peptide represent the conformational basins.

This allows to directly calculate the statistical weight of the *i* ^th^ conformation hereafter termed as *P*(*i*) and corresponding to the number of projected points falling within the *i* ^th^ basin divided by the total number of frames. From *P*(*i*) we can also estimate the free energy difference between the *i* ^th^ basin and a reference basin (with probability *P*_ref_) using the standard relation

(1) $$P\left(i\right)=P_{\mathrm{ref}}e^{\frac{-∆G_{i}{}^{\circ}}{\mathrm{RT}}},$$

$∆G_{i}{}^{\circ}$ approximately corresponds to the (standard) Gibbs free energy difference between the *i* ^th^ basin (conformation) and the reference one, assuming: (i) a negligible difference between the corresponding partial molar volumes (it should be further remarked that our simulations are performed at constant volume and not at constant pressure) and (ii) negligible differences between the (quantum) vibrational partition functions of the *i* ^th^ conformation and the reference one. Subsequently, from all the basins, we can extract a number (hereafter indicated with *K*) of peptide structures in the range 0–3 kJ/mol, hence obtaining the statistically relevant peptide conformations representing the whole basin. Note that when we are dealing with a relatively large basin, more than one representative structure could be extracted (see Section 3). Each of the *K* extracted conformations characterized by the proper statistical weight and by the corresponding free energy ($∆G_{i}{}^{\circ}$, *i* = 1, *K*) are then used for the quantum‐chemical calculations for determining the observable of interest (e.g., a spectral feature). Note that this latter step should be carried out after a preliminary constrained geometry optimization of the peptide (possibly at the same level of the quantum‐chemical approach used for the calculation of the observable), starting from the MD‐extracted structure, performed by keeping frozen the internal degrees of freedom corresponding to semi‐classical motions, that is, typically the torsion angles.

In order to take into account also the possible contribution of the solvent molecules^28^ in the observable of interest, we decided to adopt the same protocol just described for locating plausible peptide‐solvent clusters to be used for subsequent quantum‐chemical calculations. For this purpose, we performed a further analysis, again based on the ED described in the detail elsewhere^27^ and here only briefly outlined.

For each of the *K* conformations previously extracted from the free‐MD we first perform a MD simulation, with the same box and protocol used for free‐MD, with the peptide kept frozen at the center of the box. We hereafter refer to these simulations as constrained‐MD. For each of the *K* constrained‐MD we then construct an internal reference frame, centered in the frozen peptide geometrical center. The projection of the frozen peptide coordinates onto the above unit vectors provides the axes of the ellipsoid best describing the peptide shape (in this case a fixed ellipsoid for each constrained‐MD). At each of the constrained‐MD trajectory, a pre‐selected number (*N*) of solvent molecules showing the lower square distances in the metric of the above ellipsoid, are then extracted. By repeating this last step for all the frame of the constrained‐MD we can obtain a trajectory of the peptide‐(solvent)_*N*_ cluster. The ED analysis, previously described for the single peptide and now carried out on such a cluster, as well as the consequent Principal Components, provides the conformational landscape of the peptide‐(solvent)_*N*_ cluster, that is, the location of a series of basins now corresponding to solvation shell conformations. Therefore, considering the *i* ^th^ constrained‐MD, which showed a probability *P*(*i*) in the free‐MD, we can obtain, from the corresponding ED analysis, a number *M*(*i*) of solvent conformational basins, each with probability *p*(*j*,*i*), with *j* = 1 to *M*(*i*) and *i* = 1 to *K*. Obviously the sum of all the *p*(*j*,*i*) is equal to *P*(*i*). Also in this case the relative free energy of each peptide‐(solvent)_*N*_ cluster conformation can be easily calculated from the standard expression previously reported and now making use of the obtained *p*(*j*,*i*). Each of these conformations is finally extracted and optimized through quantum‐chemical calculations using internal constraints for maintaining both the peptide conformation of the *i* ^th^ basin and the relative peptide‐water positions and orientations. The obtained *M*(1) + *M*(2) + ⋯+ *M(K)* clusters, with the related statistical weight, *p*(*i*,*j*), are then used for calculating the observable of interest, that is, the spectral intensities (see the next paragraphs) are considered as the weighted sum of the spectra of each conformational state (either the single chromophore of the cluster). Given the size and the shape of the peptides we have used 30 and 40 solvent molecules for cationic GAG^+^ and (GVGVP)_2_, respectively. An additional analysis of the conformational space is considered in Figure S2 while the atomic coordinates of the clusters have been reported in Tables S1–S4.

### 2.3 Quantum chemical calculations

Once a set of significant number of representative conformations has been identified by the ED analysis, a partial geometry optimization in internal coordinates of their MD non‐equilibrated geometries is performed at the density functional theory (DFT) level.^39^ The DFT calculations have been performed employing the ADF engine of the AMS code.^40^ All the geometry optimizations, with internal constraints as described in previous Section 2.2, have been performed employing a basis set of Slater‐type orbitals of triple‐zeta quality (STO‐TZP) and the range‐separated exchange‐correlation (xc) functional wB97X‐D. Such functional includes both the dispersion and non‐local exchange asymptotical corrections, allowing a suitable description of the physics of the system (i.e., intramolecular, intermolecular hydrogen bonds and charge‐transfer excitations).^41^

Finally, the calculations of the UV‐electronic circular dichroism (UV‐ECD) and UV‐Photoabsorption spectra have been performed at the Time‐Dependent DFT (TDDFT) level analyzing the lowest 70 and 100 excited‐states for GAG^+^ and (GVGVP)_2_, respectively. In order to allow an easy comparison with the experimental data,^28^, ^29^, ^30^, ^31^ the calculated spectra have been reported in terms of *ε* (photoabsorption) and Δ*ε* (ECD). The individual UV‐ECD spectra relative to the most relevant conformations in presence of the explicit solvent are reported in Figures S3–S8. To this end, a convolution of all the spectra has been realized by employing Gaussian functions of appropriate half width at half maximum (HWHM; i.e., 0.4 eV for GAG^+^, and 0.3 eV for (GVGVP)_2_) in order to have the best consistency with the experimental resolution. Since the calculated spectra consist of discrete lines in terms of oscillator strength (*f*_0*k*_, in atomic units) for photoabsorption and rotatory strength (*R*_0*k*_, in cgs units) for ECD, the following expressions have been employed to calculate *ε* and Δ*ε* ^42^, ^43^:

(2) $$\varepsilon \left(E\right)=2.870\times 10^{4}\frac{1}{\sigma \sqrt{\pi }}\sum_{k}f_{0k}e^{-{\left(\frac{E-E_{0k}}{\sigma }\right)}^{2}},$$

(3) $$∆\varepsilon \left(E\right)=\frac{1}{2.297\times 10^{-39}}\frac{1}{\sigma \sqrt{\pi }}\sum_{k}R_{0k}E_{0k}e^{-{\left(\frac{E-E_{0k}}{\sigma }\right)}^{2}},$$

where in expressions (2) and (3) *E*_0*k*_ is the calculated excitation energy (in eV) from the ground state to the *k* ^th^ excited state, and *σ* is related to HWHM according to the following relation (4):

(4) $$\sigma =\frac{\text{HWHM}}{\sqrt{\ln\left(2\right)}}.$$

This procedure allows a direct comparison with the experimental spectra not only in terms of excitation energy but also in terms of absolute intensity.

The UV‐CD spectra of the most probable conformations at low temperature values (i.e., GAG^+^(H_2_O)_30_ at 30°C, and (GVGVP)_2_(H_2_O)_40_ at 15°C) were also calculated with the hybrid xc functional CAM‐B3LYP^44^ to evaluate the quality of our choice (wB97X‐D) which was based on the data available in the literature.^30^ The effect of the functional on the calculations is briefly discussed in Figure S9. We also evaluated the mean‐field produced by the solvent using the conductor like screening model (COSMO) of solvation^45^ as implemented in ADF. Such scheme was adopted for the tripeptide conformers (extracted from the ED analysis) at 30°C, considering both the GAG^+^ by itself and the GAG^+^ solvated with 30 water molecules. The resulting statistically weighted ECD were compared and discussed in Figure S10.

**FIGURE 1:**
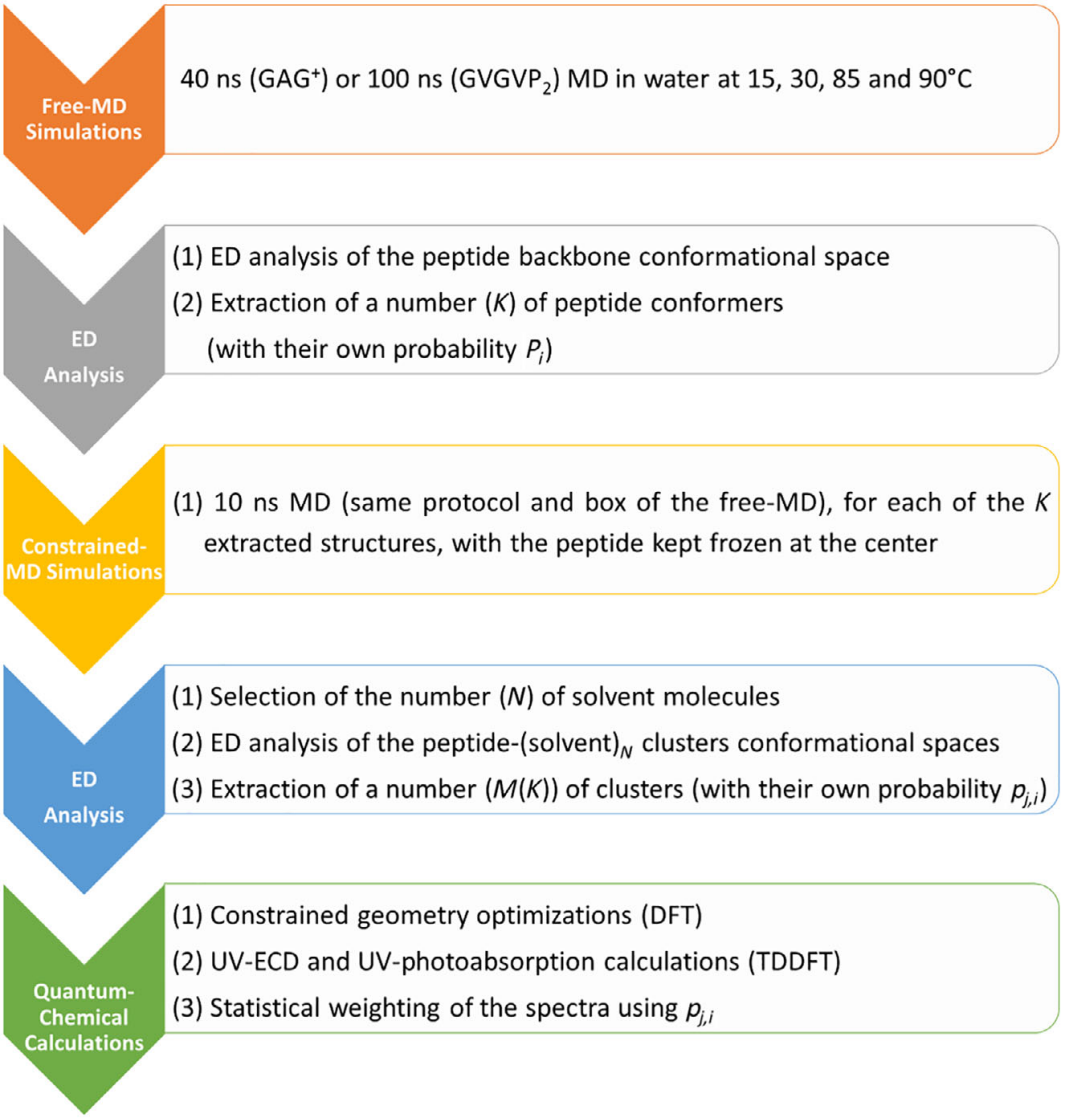
Flowchart of the procedure proposed in this work. The three methods (i.e., MD simulations, ED analysis, and quantum‐chemical calculations) are reported together with the specific conditions we used to realize the tests on the two peptides

The procedure just presented has been schematized in the flowchart shown in Figure 1 to help the visualization and comprehension of the multistep scheme we proposed.

## 3 RESULTS AND DISCUSSION

Although the main focus of the present study is to provide a computational strategy for addressing the spectra of relatively complex species in solution, we deserve the initial part of this section to briefly outline the preliminary conformational/structural analysis of the species of interest.

### 3.1 Free‐MD simulations and peptides conformational states

**FIGURE 2:**
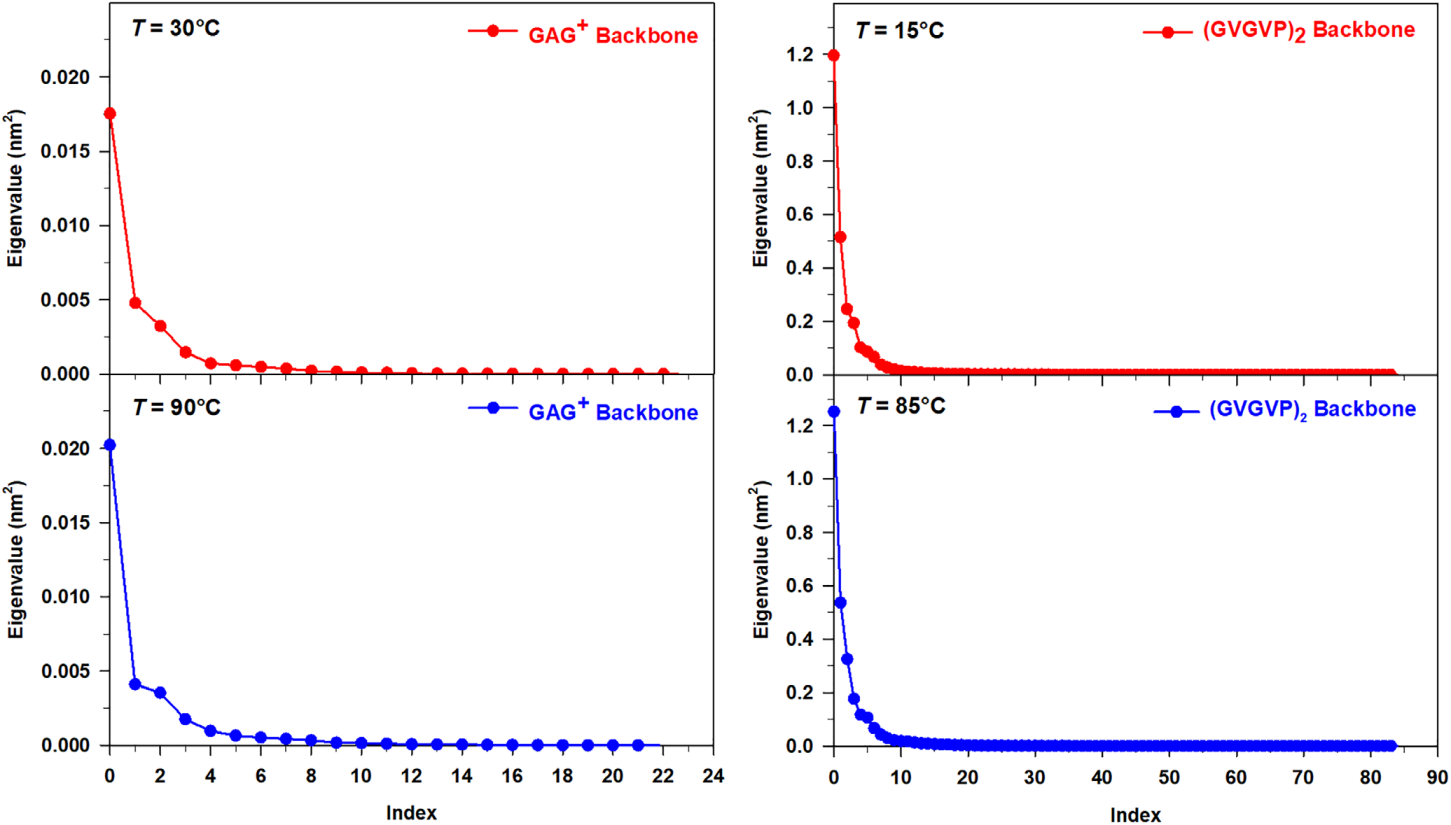
Eigenvalues of the GAG^+^ backbone covariance matrix at *T* = 30°C, and *T* = 90°C (left panels); (GVGVP)_2_ backbone covariance matrix at *T* = 15°C and *T* = 85°C (right panels)

**FIGURE 3:**
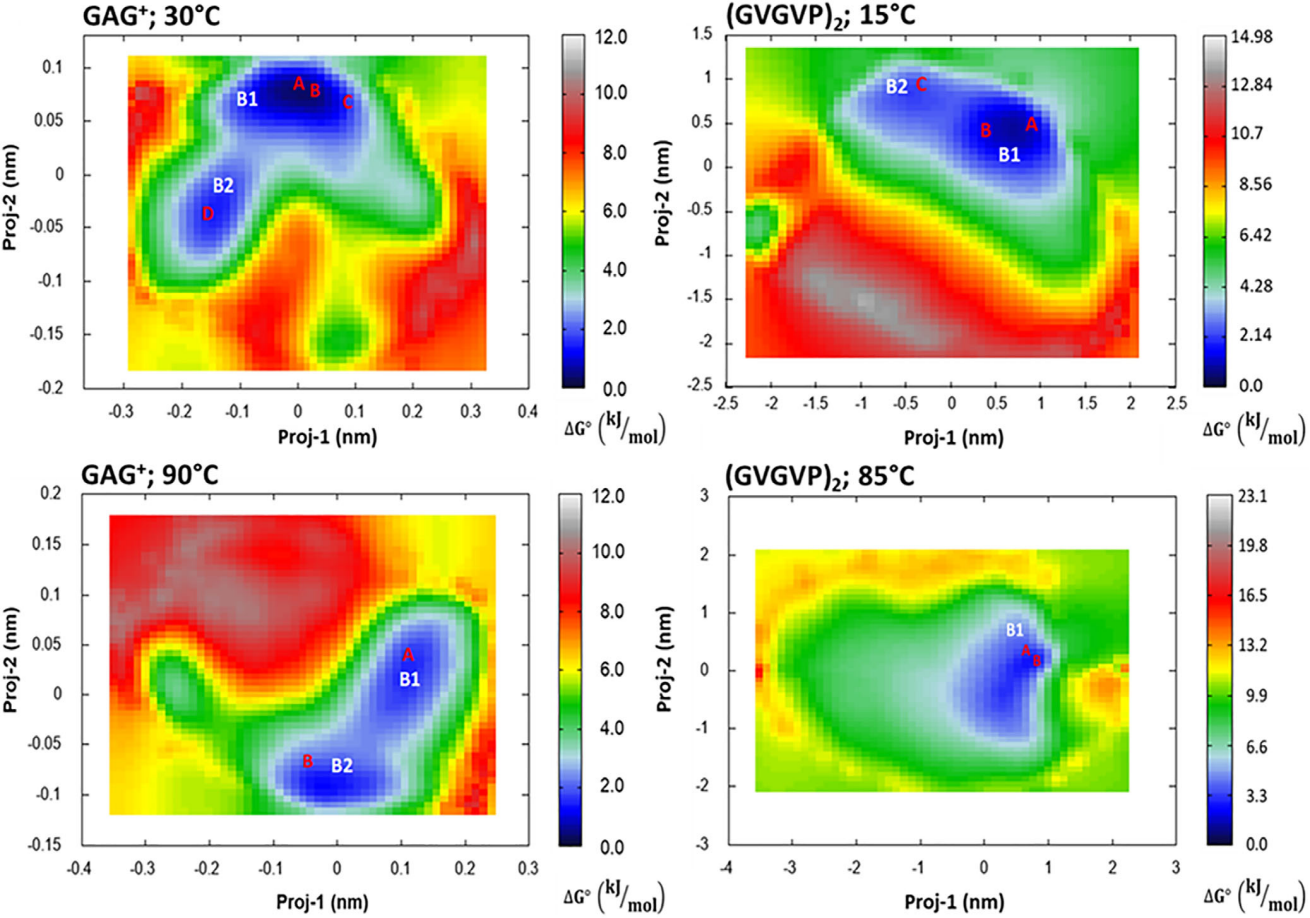
Free energy landscape (Equation 1 in kJ/mol) in the essential plane of GAG^+^ at *T* = 30°C, and *T* = 90°C (left panels); (GVGVP)_2_ at *T* = 15°C, and *T* = 85°C (right panels). The scale of the energy values is also reported as a vertical‐colored bar

In Figure 2, we report the spectrum of the eigenvalues from the diagonalization of the peptide (backbone) covariance matrix as obtained from the free‐MD simulations of the two peptides at the temperatures of interest. We first observe that, as expected, the spectrum for (GVGVP)_2_ is characterized by much larger eigenvalues (approximately 50 times larger than GAG^+^) indicating that peptides with a larger size undergo much higher fluctuations (see also the Figure 3). At the same time, it is also worth of comment that in both the systems the change of temperature does not induce a drastic change in the shape, that is, in the trace of the covariance matrix and hence in the whole backbone fluctuation. As a matter of the fact when passing from the lower to the higher temperature a sharp although not dramatic increase of the highest eigenvalues is systematically observed. Finally, we also wish to remark that, as usual for relatively small peptides,^26^ a large fraction (between 65% and 75%) of the whole peptide fluctuation, corresponding to the trace of the covariance matrix, can be safely described using only two eigenvectors of the backbone covariance matrix.

The corresponding Principal Components were then used for obtaining the free energy landscapes reported in the Figure 3.

**FIGURE 4:**
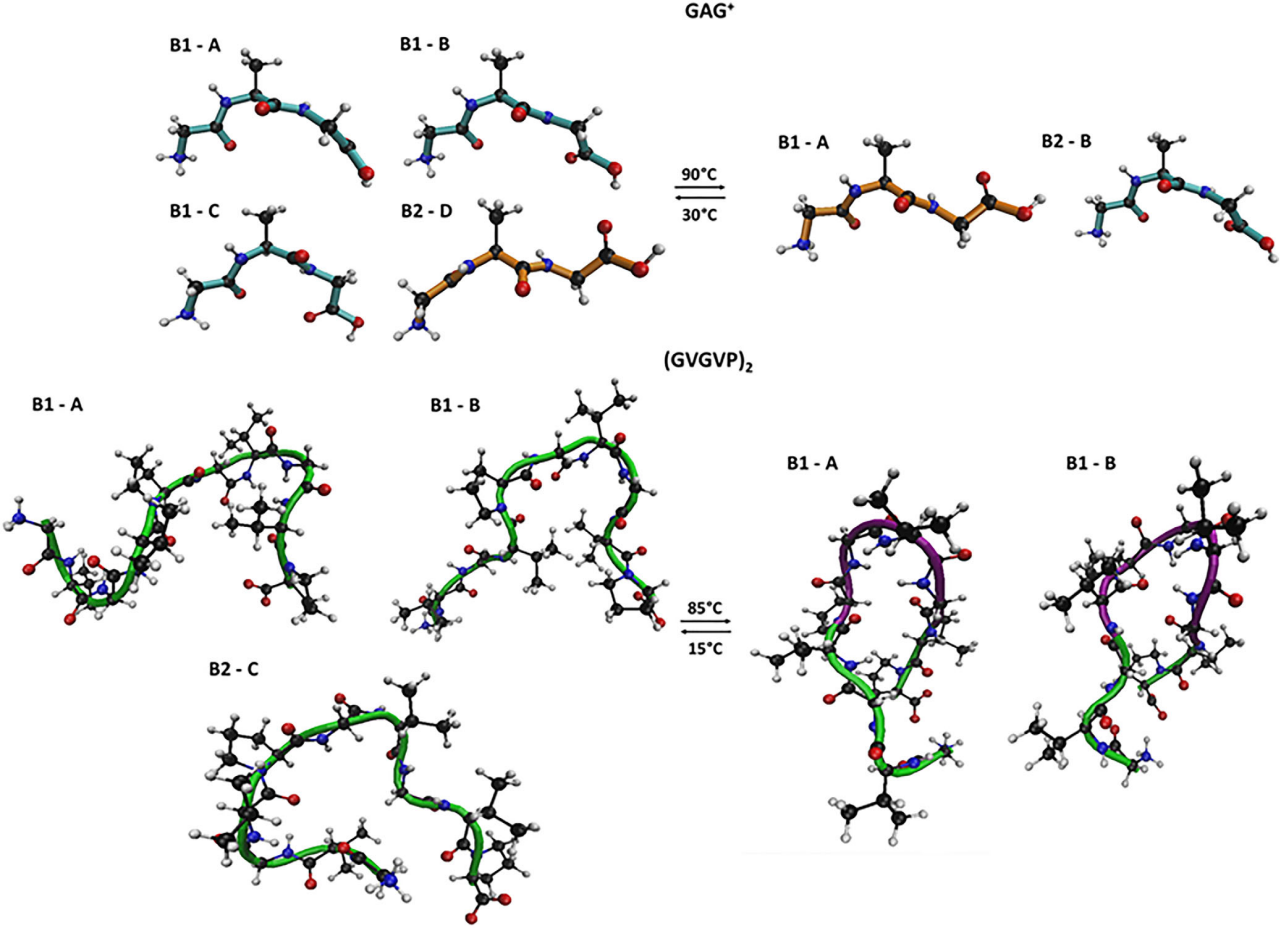
Schematic picture of the representative structures extracted by the free energy landscape of Figure 3

In the same Figure 3, we have also shown the conformational basins whose relative free‐energies, calculated using Equation (1), are reported in Table 1. The corresponding basin representative structures, extracted for subsequent quantum‐chemical calculations are reported in the Figure 4.

**TABLE 1:** Relative free energies, at the given temperatures, for GAG^+^ and (GVGVP)_2_ basins extracted from free‐MD simulations (see also Figures 3 and 4)

Peptide	Temperature (°C)	Basin – solute structure	Relative free energy (kJ/mol)
GAG^+^	30	B1 – A	0.7
GAG^+^	30	B1 – B	0.0
GAG^+^	30	B1 – C	2.4
GAG^+^	30	B2 – D	1.6
GAG^+^	90	B1 – A	1.6
GAG^+^	90	B2 – B	0.0
(GVGVP)_2_	15	B1 – A	1.8
(GVGVP)_2_	15	B1 – B	2.9
(GVGVP)_2_	15	B2 – C	2.7
(GVGVP)_2_	85	B1 – A	0.0
(GVGVP)_2_	85	B1 – B	2.9

First of all, it is interesting to observe, in the Figure 3, the different Principal Components values further reflecting the already remarked differences in the peptides whole fluctuation: the proj‐1 and proj‐2 domains become much wider when passing from GAG^+^ to (GVGVP)_2_ and, although to a lesser extent, upon the temperature increase. In the case of GAG^+^, we also systematically observe the presence of a bimodal distribution, that is, the presence of two low (free) energy conformational states in relatively fast interconversion.

In line with the experimental data GAG^+^ shows a relatively high propensity of adopting a pPII state represented by the basin B1 at 30°C. Moreover, confirming the low‐entropy character of pPII conformation, upon temperature increase such a motif (B1) becomes comparable in stability with the other structure B2, which more closely resembles the beta‐strand one. On the other hand, the conformational space of (GVGVP)_2_ appears as most sensitive to the temperature hence suggesting a more relevant entropic weight. In particular at higher temperature the conformations identified by our analysis strongly resemble beta‐turn structures both characterized by a relatively stable Val4‐Gly8 H‐bond. On the other hand, at lower temperatures the structures appear as slightly more extended, and almost completely devoid of intramolecular H‐bonds. In conclusion: our simulations confirm that the investigated systems exist in two main conformations (i.e., pPII and beta‐strand for GAG^+^; random coil and β‐turn for (GVGVP)_2_) whose ratio depends on the temperature of work.^28^, ^29^, ^31^ These systems have been then utilized with their own statistical weight, as obtained from the relative free energies, for the quantum‐chemical calculations as described in Section 2.3 and, at the same, for the subsequent analysis of the solvation shells as described in Section 2.2 and reported in the next paragraph.

### 3.2 Constrained‐MD and water‐peptide clusters conformational states

**FIGURE 5:**
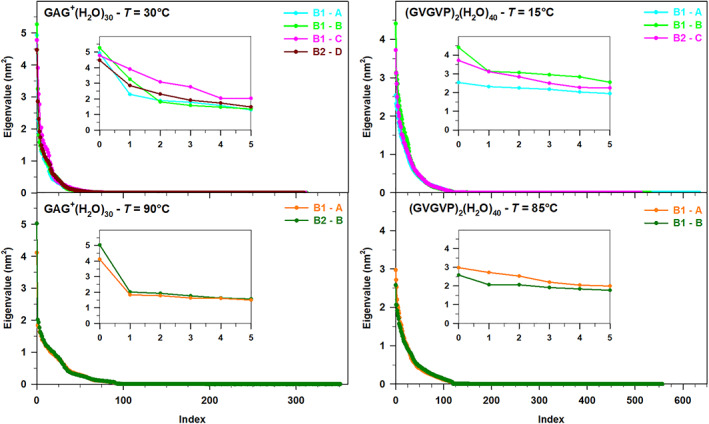
Spectrum of the eigenvalues of the covariance matrix of the peptide‐(solvent)_*N*_ clusters extracted from the constrained‐MD simulations. The first six eigenvalues have been highlighted in the inset for all the systems

In the Figure 5, we have reported the spectra of the eigenvalues from the diagonalization of the positional covariance matrix for the constrained‐MD trajectories, as obtained from the procedure previously described. Differently from the single peptide (Figure 2) in this case the spectra of the eigenvalues appear as much less steep confirming that when a non‐covalent system like the peptide‐(solvent)_*N*_ cluster is concerned, the overall fluctuation is (not surprisingly) spread over a higher number of internal degrees of freedom.^27^

It follows that in this case the conformational analysis, and hence the identification of the conformational basins should be carried out in an *N*‐dimensional (*N* > 2) hyperspace. This procedure, although possible in principle^46^ can represent a very complicated task and, hence, a compromise between quality of the result and computational difficulty is necessary. For this reason, also in this case we projected the water trajectory, with respect to the frozen peptide, onto the plane formed by the first two eigenvectors of Figure 5, being well aware of the possible lack of some details for identifying the conformational repertoire of the water molecules in closer interaction with the peptide.

**FIGURE 6:**
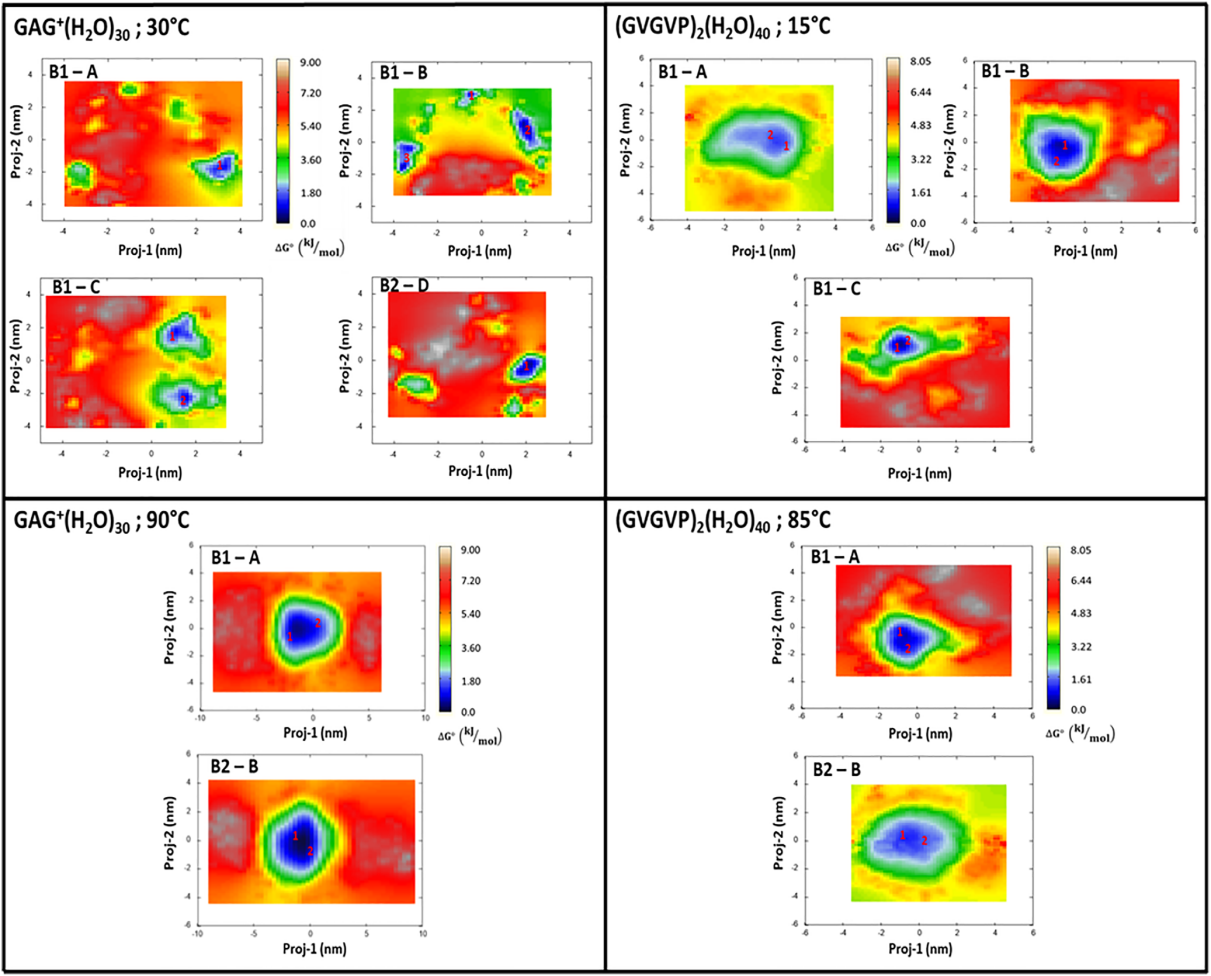
Free energy landscape (Equation 1, in kJ/mol) in the essential plane of GAG^+^ clusters at *T* = 30°C, and *T* = 90°C (left panels) and (GVGVP)_2_ cluster at *T* = 15°C, and *T* = 85°C (right panels). The peptide conformation (see Figure 4) is indicated in the upper left‐side of each inset. The scale of the energy values is also reported as a vertical‐colored bar

The free‐energy landscapes, with the corresponding free‐energy basins also in this case obtained using Equation (1), are shown in Figure 6. In this case, a general increase of the Principal Components, if compared to the single peptide results (Figure 3) is observed. Such a result obviously reflects the already commented spectrum of the cluster covariance‐matrix eigenvalues and, more precisely, the much higher mobility of the water molecules and the involvement of an increased number of internal degrees of freedom. Additional details on the selected (low free‐energy) basins indicated in Figure 6 are collected in Table 2. The representative structures of these basins, whose details can be found in the Supporting Information, were then used—with their own statistical weight and after a constrained optimization—for the calculation of the observables of interest as reported in the next paragraph.

**TABLE 2:** Relative free energies, at the given temperatures, for GAG^+^(H_2_O)_30_ and (GVGVP)_2_(H_2_O)_40_ cluster basins extracted from constrained‐MD simulations (see Figure 6)

Peptide	Temperature (°C)	Basin – cluster	Relative free energy (kJ/mol)
GAG^+^	30	B1 – A1	2.2
GAG^+^	30	B1 – B1	0.0
GAG^+^	30	B1 – B2	2.4
GAG^+^	30	B1 – B3	1.5
GAG^+^	30	B1 – C1	3.7
GAG^+^	30	B1 – C2	4.8
GAG^+^	30	B2 – D1	4.1
GAG^+^	90	B1 – A1	1.6
GAG^+^	90	B1 – A2	3.9
GAG^+^	90	B2 – B1	0.1
GAG^+^	90	B2 – B2	1.9
(GVGVP)_2_	15	B1 – A1	1.8
(GVGVP)_2_	15	B1 – A2	3.3
(GVGVP)_2_	15	B1 – B1	3.4
(GVGVP)_2_	15	B1 – B2	3.8
(GVGVP)_2_	15	B2 – C1	3.0
(GVGVP)_2_	15	B2 – C2	4.3
(GVGVP)_2_	85	B1 – A1	0.0
(GVGVP)_2_	85	B1 – A2	0.5
(GVGVP)_2_	85	B1 – B1	3.6
(GVGVP)_2_	85	B1 – B2	4.8

### 3.3 UV‐CD spectra

The UV‐CD spectrum has been calculated for each of the structures representing the basins collected in Table 2, then the overall spectra have been obtained by summing the individual ones weighted accordingly to the procedure described in previous Section 3.2. Hence, four final UV‐CD spectra (two for GAG^+^, and two for (GVGVP)_2_) have been compared with the experimental ones available in the literature.^28^, ^29^, ^31^

**FIGURE 7:**
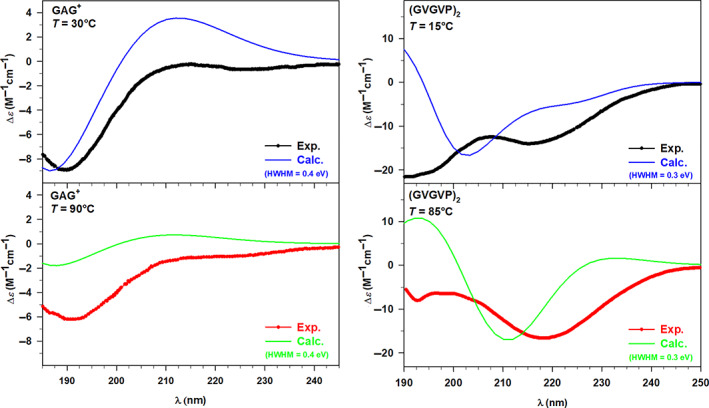
Left panels: Experimental (Exp.) and calculated (Calc.) UV‐circular dichroism (UV‐CD) spectra of GAG^+^ in water at 30°C (upper panel) and 90°C (lower panel). Right panels: Experimental (Exp.) and calculated (Calc.) UV‐CD spectra of (GVGVP)_2_ in water at 15°C (upper panel) and 85°C (lower panel). The GAG^+^ and (GVGVP)_2_ calculated spectra have been convoluted with Gaussian functions with HWHM of 0.4 and 0.3 eV, respectively

The general assessment of the spectra reported in the four panels of Figure 7 highlights the overall accuracy of our model, which allows us to coherently simulate the behavior of the selected systems. Indeed, the salient features of the experimental UV‐CD spectra are reproduced by the calculated ones, particularly well for the GAG^+^ system as shown in the left panels of Figure 7. At both temperature values, the experimental minimum around 190 nm has been observed in the corresponding calculated spectra, although some discrepancies in terms of absolute Δ*ε* can be found at 90°C (lower left panel of Figure 7). Moving toward lower energy values the calculated spectra follow nicely the increasing trend, which reaches its maximum around 212 nm. On the other hand, the experimental UV‐CD spectrum of (GVGVP)_2_ in water at 15°C reveals the presence of two minima at 197 and 218 nm, respectively, the first one is found as well in the calculated spectrum at 203 nm, while the calculated counterpart of the second minimum is a shallow inflection at 224 nm. Despite this moderate energy shift, once again the model takes into account the physics of the described system. At higher temperature values, the experimental spectrum is mostly characterized by the minimum at 218 nm, well reproduced by the theory at 211 nm.

Now we may focus in more detail on the relations among the conformational space, the optical response, and the temperature. Along the ED analysis, we have already pointed out the influence of the temperature on the conformers, while variations of the spectroscopic response related to the temperature have just been mentioned. Therefore, all the parameters can be collected together analyzing the UV‐CD spectra in terms of both temperature and conformational dependence, thus verifying if our model considers all the effects in a balanced way. For such an analysis, it is convenient to compare together the experimental CD spectra at different temperatures in order to point out qualitative trends, and then to verify if such trends are reproduced, at least partially, by the calculated spectra.

**FIGURE 8:**
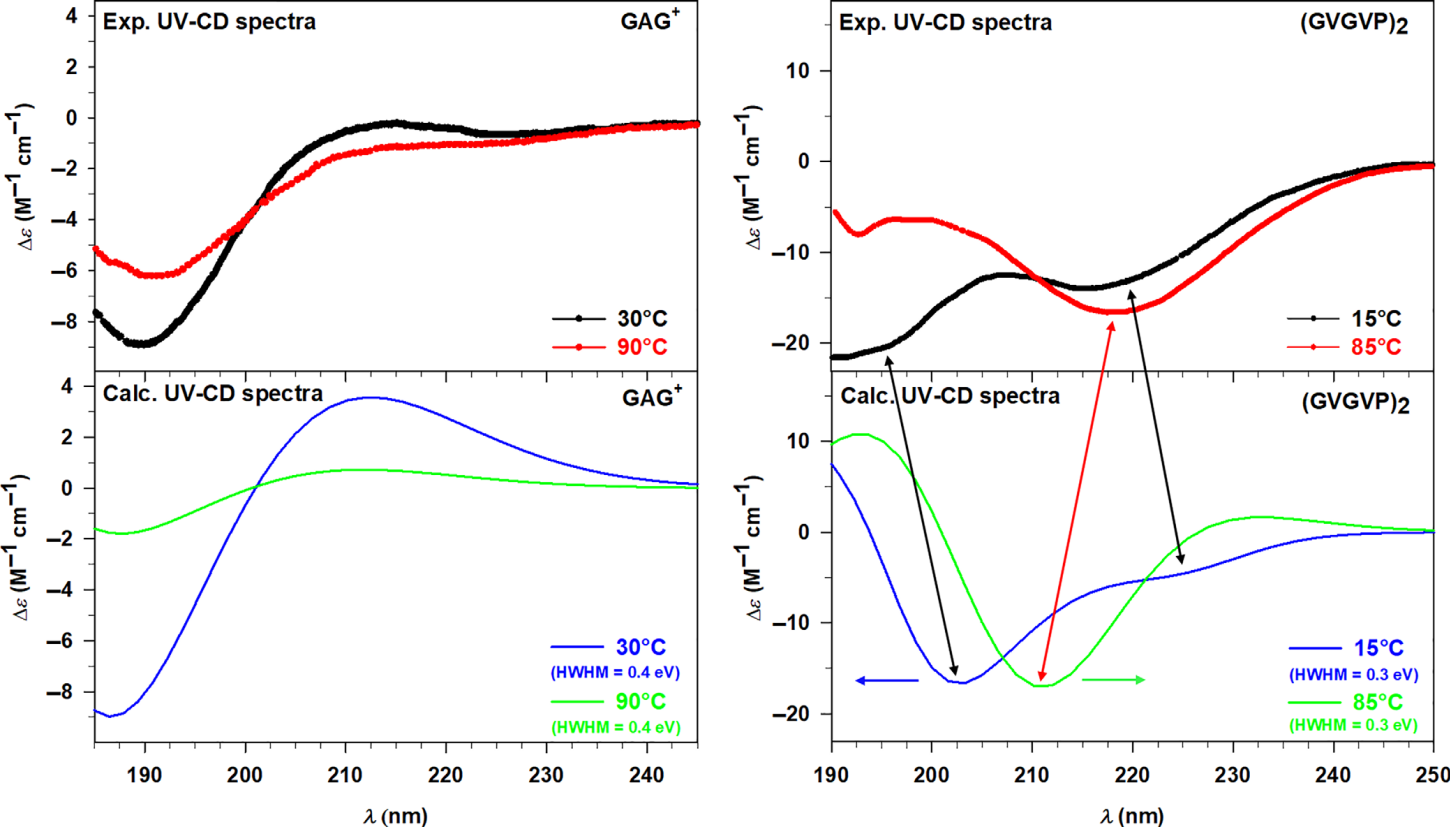
Left panels: Experimental (Exp.) UV‐circular dichroism (UV‐CD) spectra at 30 and 90°C (upper panel) and calculated (Calc.) UV‐CD spectra at 30 and 90°C (lower panel) of GAG^+^ in water. Right panels: Experimental (Exp.) UV‐CD spectra at 15 and 85°C (upper panel) and calculated (Calc.) UV‐CD spectra at 15 and 85°C (lower panel) of (GVGVP)_2_ in water. The GAG^+^ and (GVGVP)_2_ calculated spectra have been convoluted with Gaussian functions with HWHM of 0.4 and 0.3 eV, respectively

The experimental UV‐CD spectra recorded for GAG^+^ in water at 30 and 90°C show similar patterns, with the only significant difference related to the intensity. Indeed, both the spectra are characterized by a minimum point around 190 nm followed by an increasing trend at lower energy values. Looking at Figure 8 (lower left panel), it can be easily noticed that the calculated UV‐CD spectra closely resemble such behavior only showing a more marked amplitude in intensity, especially around the minimum region. Nevertheless, the discrepancies in terms of intensity distributions are so modest that do not affect the general qualitative agreement between theory and experiment. Therefore, we can conclude that the calculated spectra well reproduce the decrease of CD that occurs increasing the temperature, which can be interpreted considering the two possible conformations of the tripeptide GAG^+^. It is known from the literature^28^, ^29^ that these short peptides mostly exist in unordered conformations which are in this specific case the pPII and β‐strand structure. The populations of the conformers are strongly influenced by the temperature, thus determining the preference for the first conformation at lower *T* values, and for the latter one at higher *T* values. Hence, the reduction of the signal at the minimum corresponds to a decrease of the pPII population within the conformational space, also found and supported by our theoretical approach. Indeed, the peptide structures (see Figure 4 of the Section 3.1) that we have selected at each temperature from the ED analysis show how the conformations move from having the folded extremities (pPII) toward a more extended structure (β‐strand), corroborating the results just discussed.

Despite the evident influence of the conformations on the optical response, the GAG^+^ system is characterized by two possible similar structures, both unordered. Therefore, we have chosen the larger system (GVGVP)_2_ which, as known from the literature,^28^, ^29^ exhibits two possible conformations that present much more marked differences. From this perspective, we can discuss the temperature effect on the UV‐CD spectra of (GVGVP)_2_ in correlation with its possible conformations, starting from the experimental data shown in the upper right panel of Figure 8. The two UV‐CD spectra of (GVGVP)_2_ in water present some trend differences, particularly in the higher energy region (190–205 nm) where the spectrum at 15°C is characterized by a minimum, while that at 85°C by a maximum. From the isodichroic point (210 nm) on, the two spectra start showing a similar pattern defined by a minimum around 218 nm followed by an increasing behavior. Looking at the lower right panel of Figure 8, we can point out that a similar behavior has been qualitatively reproduced by our calculations. In addition, the first calculated isodichroic point has been found around 206 nm (slightly blueshifted with respect to the experiment) and the spectra present the minimum (85°) or at least an inflection (15°) at lower energy values (211–227 nm). A second isodichroic point is found in the calculation but is not present in the experiment. However, as stated above, some discrepancies in terms of energy and intensity distributions are noticed, affecting the accuracy of the results, which can be rationalized considering the predominant random coil structure at low *T* values and of the ordered β‐turn conformation at high *T* values. Furthermore, it is interesting to notice the presence of a small population of ordered structures also at 15°C, which defines the second less marked minimum point. Therefore, the temperature influence determines the conversion from an unordered conformation toward a folded structure characterized by intramolecular H bonds. Employing our theoretical approach, we have been able to extract the same two conformations (see Figure 4 of the Section 3.1) from the corresponding conformational spaces, thus corroborating the reliability of the method.

### 3.4 Analysis of the solvent effect on the optical response

The effect of the solvent on the spectral features of a solvated chromophore is twofold. On the one hand, we can first consider an *indirect* effect consisting in the solvent‐driven conformational transitions of the solute; on the other hand, if in the presence of a very polar solvent and a degree of local interactions with the chromophore (H‐bond), the solvent might produce a *direct* effect both produced by the bulk effects and by the electronic coupling between the solvent molecules directly linked to the chromophore and the chromophore itself which might significantly modify the electronic states as well as the chromophore geometry. In this last part of the study, we decided to address the *direct* solvent effect more explicitly on the optical response of the investigated species.

**FIGURE 9:**
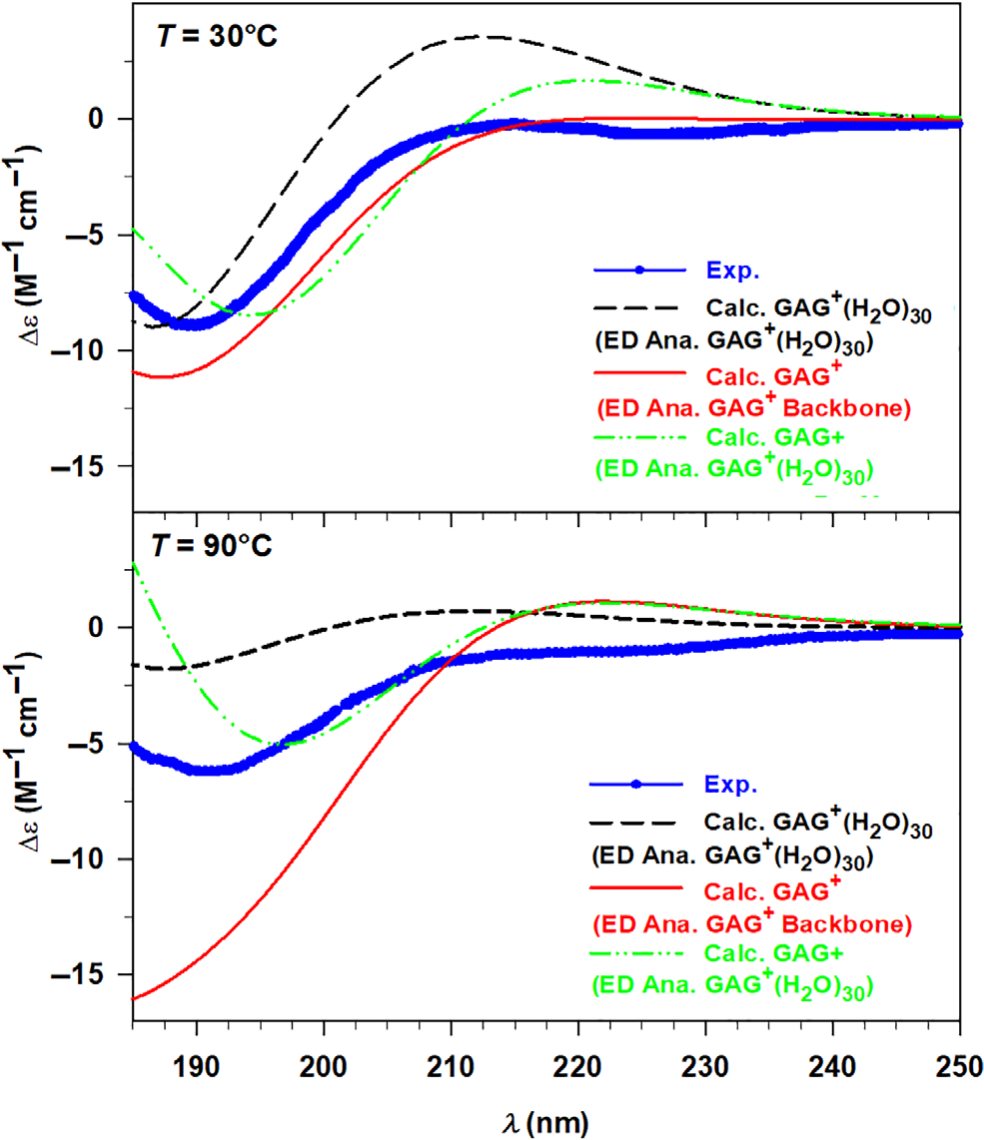
Experimental (Exp., blue dots) and calculated (Calc., black dashed line) UV‐circular dichroism spectra of GAG^+^ in water at 30°C (upper panel) and 90°C (lower panel). The two spectra have been compared with those calculated considering only the peptide structures extracted from the ED analysis of the backbone (red solid line) and only the final peptide structures extracted from the ED analysis of the clusters (green dotted line). All the calculated spectra have been convoluted by using Gaussian functions with HWHM of 0.4 eV.

For this purpose, we have compared the UV‐CD spectra of the GAG^+^ peptide structures extracted from free‐MD, reported in Table 1, with their own relative weights with the same structures in the presence of explicit water molecules, reported in Table 2. Obviously, the conformations of the peptides, kept frozen along the second MD simulations, are similar to the structures of the second analysis. However, the geometries employed for the quantum‐chemical calculations are not identical. Indeed, the constrained optimizations carried out with or without the solvent bring to slightly different relaxations of the peptide structures, which can be pointed out by the UV‐CD spectroscopy, very sensitive to the conformational details. Therefore, such a double analysis allows us to understand how the solvent influences the conformations of GAG^+^ and the relevance of its inclusion for the calculation of the spectra. All these results have been collected in the following Figure 9.

**FIGURE 10:**
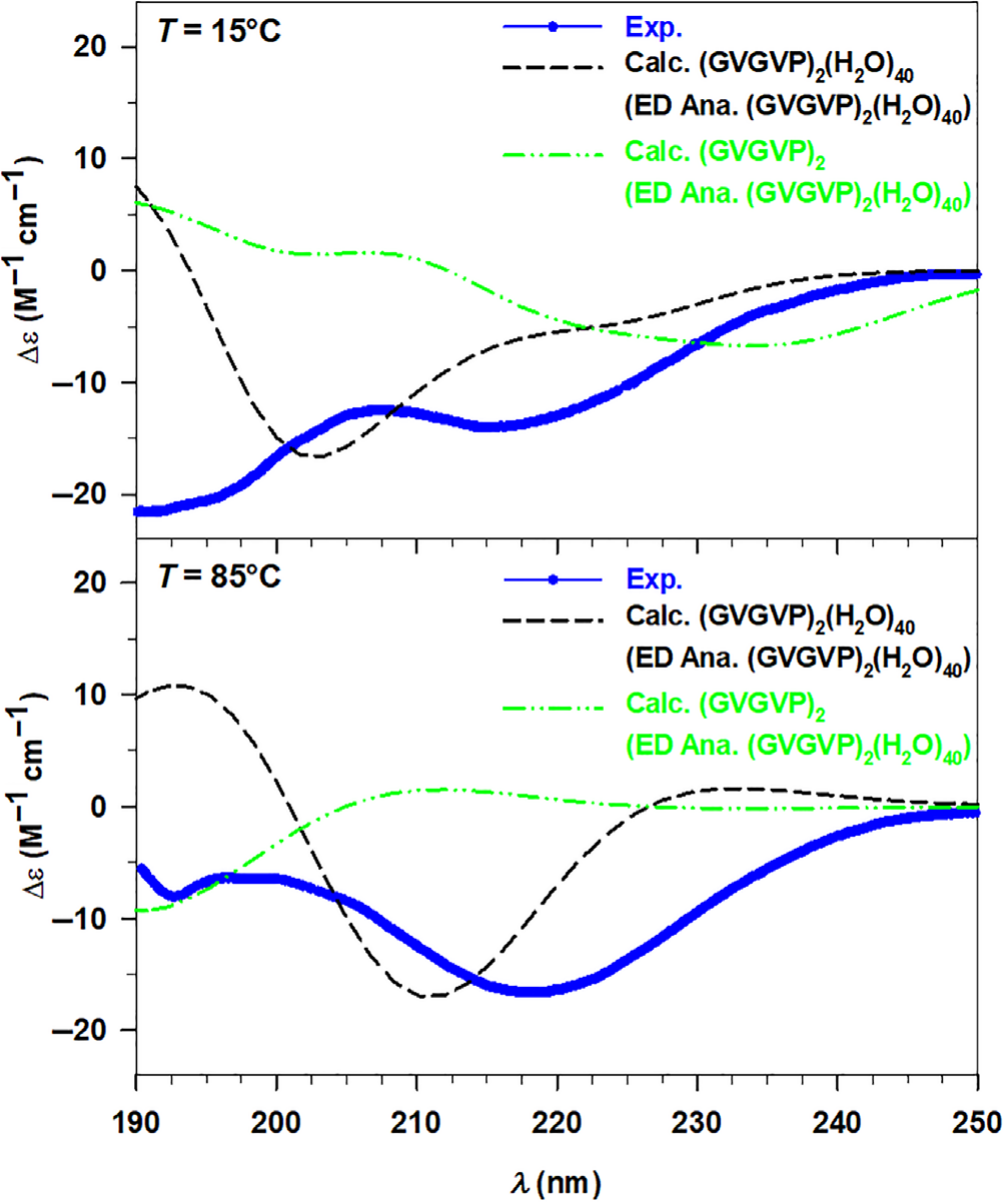
Experimental (Exp., blue dots) and calculated (Calc., black dashed line) UV‐circular dichroism spectra of (GVGVP)_2_ in water at 15°C (upper panel) and 85°C (lower panel). The two spectra have been compared with those calculated considering only the final peptide structures extracted from the ED analysis of the clusters (green dotted line). All the calculated spectra have been convoluted by using Gaussian functions with HWHM of 0.3 eV.

The results shown in Figure 9 reveal a clear effect of the water at different levels. First, at both the *T* values of interest, a worse description of the electronic response arises including only the peptide conformers extracted from the first ED analysis (i.e., ED analysis of the GAG^+^ backbone). The poorer agreement with the experimental spectrum becomes more evident at 90°C, where the minimum around 190 nm displays a much higher CD. This result suggests that the extraction of conformations based only on the free energy analysis of the solute is insufficient for a proper description of the optical response. Hence, the solvent must be included at least in the relaxation of the peptide structure. This hypothesis has been confirmed by considering the conformations extracted and relaxed within the water clusters but without explicit solvent in the calculation of the CD spectra (green lines in Figure 9). Such spectra give already a good agreement with the experimental ones, in particular at 90°C, but the inclusion of the solvent reduces the differences between the experimental and calculated energy distributions. We have applied the same procedure to the second system, the (GVGVP)_2_, calculating the spectra for the peptide structures previously obtained within the ED analysis of the clusters and optimized in presence of the 40 water molecules. The new results have been compared with the experimental spectra, as well as the overall calculated spectra, and shown in Figure 10.

In this second case, the influence of the solvent on the optical response is more evident than in the previous system and can be justified with the impact of the water molecules on the conformations. Indeed, the weighted UV‐CD spectrum calculated for the peptides at 15°C shows an opposite trend with respect to that obtained for the cluster spectrum, and for the experimental one, where the first minimum is the most pronounced. Significant differences can be found for the calculated spectrum at 85°C as well, where the minimum is strongly blueshifted and no qualitative agreement with the experimental result can be pointed out. To conclude our analysis, the small GAG^+^ system immediately reveals the effect of the water solvent, not only affecting the solute geometry but also providing a non‐negligible electronic effect on the calculated observable, and thus highlights the need of its indirect inclusion for a suitable calculation of the UV‐CD spectra. Instead, the direct influence of the solvent on the optical response seems to be more evident when larger systems are considered, for instance the short elastin‐like peptide.

## 4 CONCLUSIONS

A computational protocol aimed at modeling the UV‐CD spectra of solvated species and consisting of quantum‐chemical calculations on a series of conformations of a flexible chromophore or on a series of chromophore/solvent clusters extracted from MD simulations, is presented in this study and applied to the aqueous cationic tripeptide GAG^+^ and to the aqueous neutral decapeptide (GVGVP)_2_. The main aim of this study, beyond the description of the method, is to show its physically coherence that is expressed in: (i) its ability in taking into account the conformational repertoire of a solute with reference to the physical conditions of the experiment (temperature, ionic strength, type and density of solvent, etc.); (ii) its ability of directly measuring the actual statistical weight of each conformational state; (iii) its ability in providing us with a reliable quantum‐chemical method able of reproducing the observable of interest. The specific feature of the proposed method is its almost zero dependence on the arbitrariness to which it is often necessary to refer for the selection of the different conformational states of the chromophore or involved chromophore/solvent clusters. In the specific case presented in this study, the method satisfactorily reproduces the GAG^+^ and (GVGVP)_2_ UV‐CD spectra and, most importantly, their sensitivity to temperature variations. On the other hand the proposed method might intrinsically suffer from: (i) limitations raising from the semi‐classical nature of the utilized force field which, moreover, might be sometimes not available (and hence its construction might be necessary) particularly when dealing with species different from the peptides; (ii) a certain degree of arbitrariness in the choice of the number of solvent molecules defining the chromophore(cluster)_*N*_ species; (iii) the *possibly flat* spectrum of the CM eigenvalues which would make it very difficult the preliminary analysis on the chromophore conformational repertoire; (iv) the *certainly flat* spectrum of the CM eigenvalues for the chromophore(cluster)_*N*_ species which—as already remarked—might produce an incomplete conformational repertoire. However, our method definitely demonstrates the importance, at least for the systems addressed in the present case, of a physical coherent inclusion of the solvation shells closer to the peptides for the final CD modeling.

## Supporting information


**Appendix S1** Stick and ball model of the cationic GAG tripeptide and the GVGVPGVGVP decapeptide, additional analysis of the conformational spaces, individual calculated ECD spectra of the (GVGVP)_2_(H_2_O)_40_ clusters at 15°C and 85°C, individual calculated (fitted and discrete) ECD spectra of GAG^+^(H_2_O)_30_ and (GVGVP)_2_(H_2_O)_40_ at 15°C, 30°C, 85°C and 90°C, effect of the xc functional on the ECD spectra, UV‐ Photoabsorption Spectra, effect of the solvent mean‐field on the ECD spectra, and atomic coordinates of the clusters.

## Data Availability

Data available in article Supporting Information.
