# Supplementary material for: A computational approach for modeling electronic circular dichroism of solvated chromophores
Source: J Comput Chem. 2022 Sep 22;43(30):2023–36. doi: 10.1002/jcc.27001 (PMC9825941; doi:10.1002/jcc.27001)
Supplement: Supplementary file 1 — Appendix S1 Stick and ball model of the cationic GAG tripeptide and the GVGVPGVGVP decapeptide, additional analysis of the conformational spaces, individual calculated ECD spectra of the (GVGVP)2(H2O)40 clusters at 15°C and 85°C, individual calculated (fitted and discrete) ECD spectra of GAG+(H2O)30 and (GVGVP)2(H2O)40 at 15°C, 30°C, 85°C and 90°C, effect of the xc functional on the ECD spectra, UV‐ Photoabsorption Spectra, effect of the solvent mean‐field on the ECD spectra, and atomic coordinates of the clusters. [file JCC-43-2023-s001.doc]

**SUPPORTING INFORMATION**


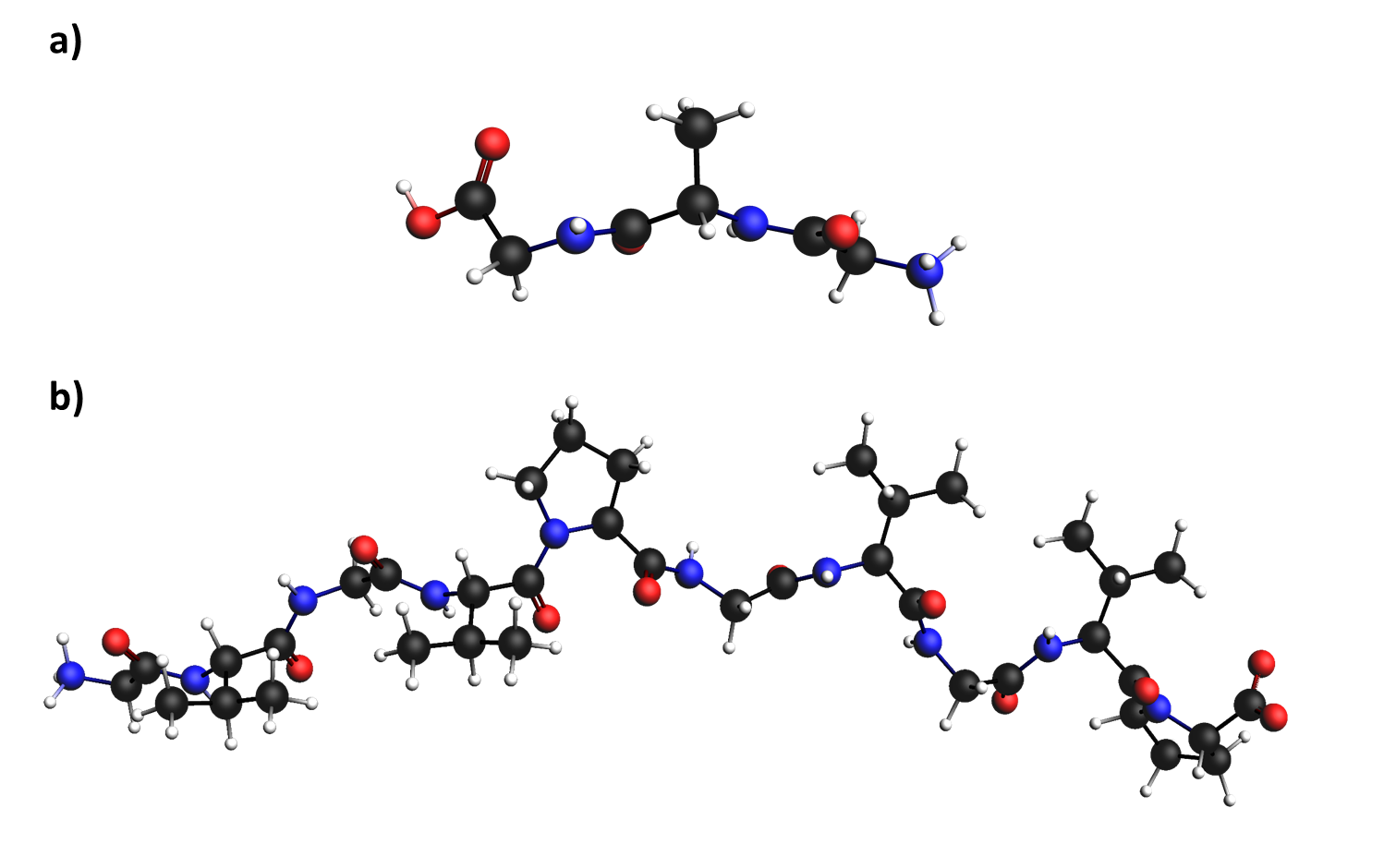


**Figure S1.** Stick and ball model of a) the cationic GAG tripeptide and b) the GVGVPGVGVP decapeptide. O, C, N, and H atoms are reported as red, black, blue, and white, respectively.

**Additional analysis of the conformational spaces**

We conducted a further analysis of the conformational spaces of the GAG^+^ backbone at the two selected T values, consisting in the projection of both the trajectories along the plane of the first two essential modes at the lower temperature (i.e., 30°C). The same analysis was then repeated for the (GVGVP)_2_ and all the results are collected in Figure S2.


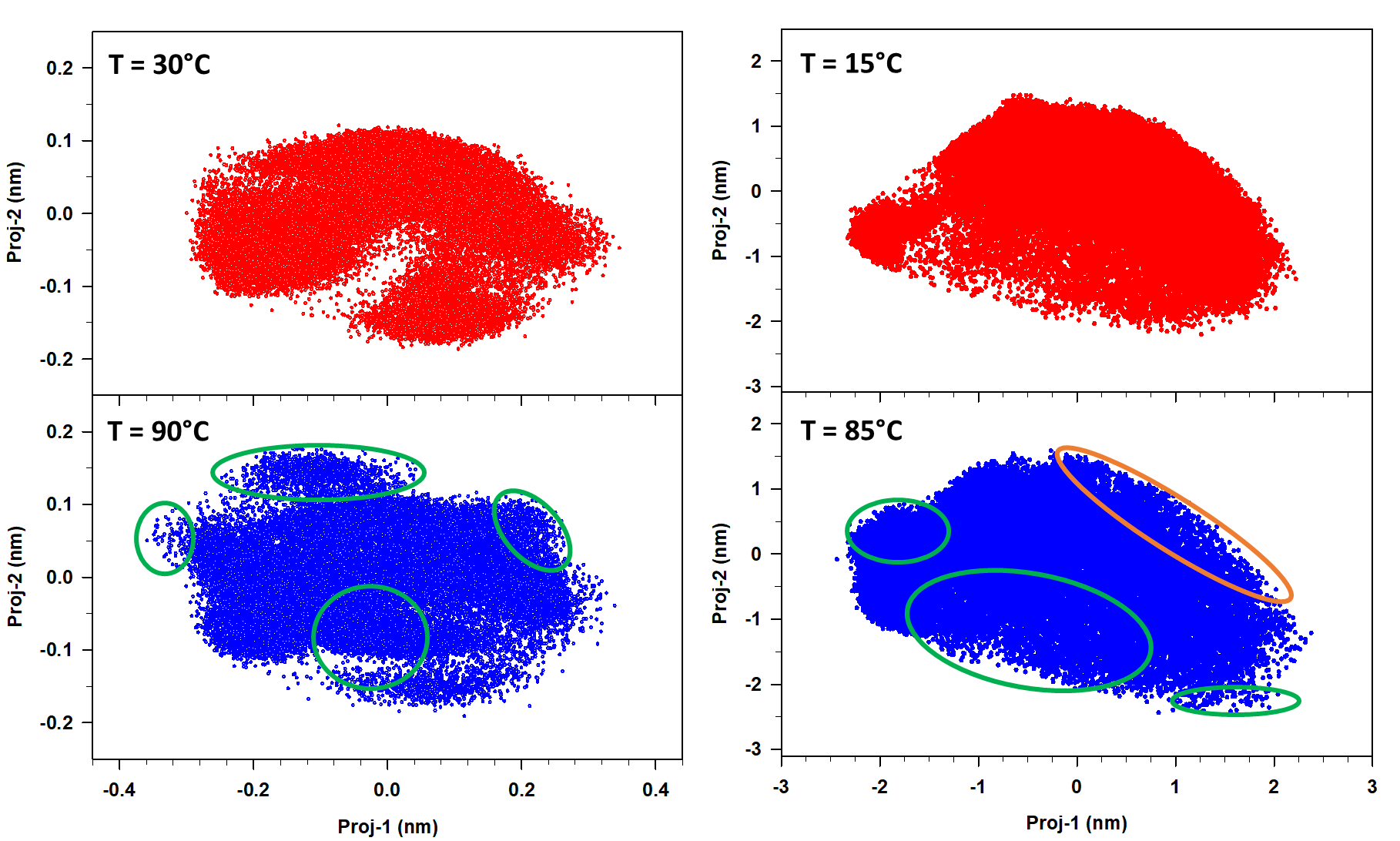


**Figure S2.** Conformational spaces of GAG^+^ (left panels) at 30°C (upper panel) and 90°C (lower panel), and (GVGVP)_2_ (right panels) at 15°C (upper panel) and 85°C (lower panel). The regions populated at high T values are circled in green, while the regions partially depleted ad high T values are circled in orange.

Figure S2 reveals that the populations which define the two conformational spaces are very similar, with only small regions that become filled increasing the temperature (green regions in the left panel of Figure S2), as expected by the dependence among temperature and accessible motions discussed in the main article. This result is naturally correlated to the small dimensions of the tripeptide which hence can be considered as a good starting point for the validation of the model but limited by its small size. Therefore, we have repeated such analysis of the conformational space for (GVGVP)_2_, following the procedure explained above for the GAG^+^ case. The comparison between the conformational spaces herein shows the increased population of states at higher T values, as well as a slight depletion of the upper right region (green and orange regions, respectively, in the low right panel of Figure S2), thus suggesting larger conformational differences induced by the temperature.

**Individual calculated ECD spectra of the (GVGVP)_2_(H_2_O)_40_ clusters at 15°C and 85°C**


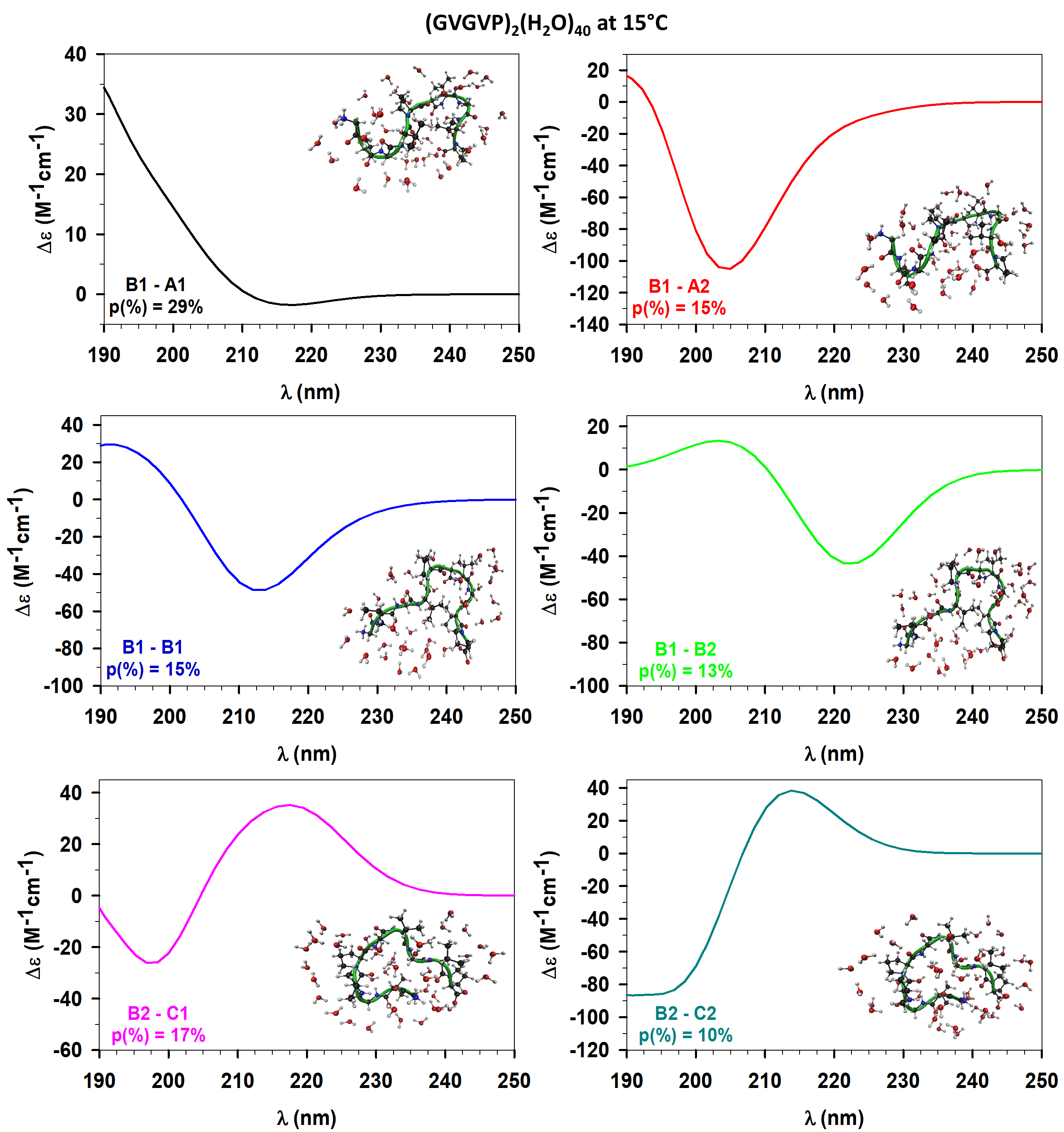


**Figure S3.** Individual calculated ECD spectra of the (GVGVP)_2_(H_2_O)_40_ clusters at 15°C. The labels of the structures are reported in the Figure together with the corresponding statistical weights and the representations of the clusters.


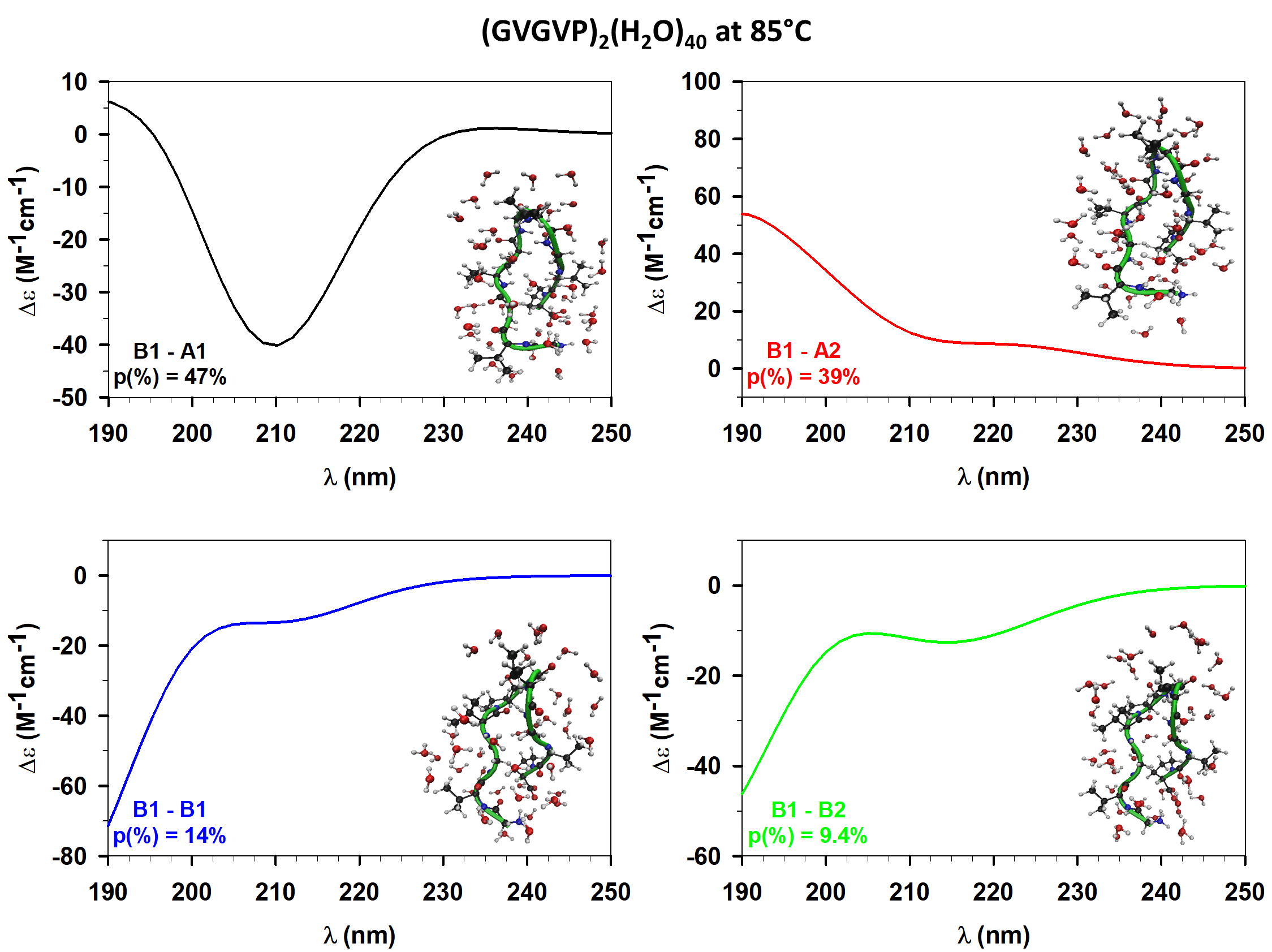


**Figure S4.** Individual calculated ECD spectra of the (GVGVP)_2_(H_2_O)_40_ clusters at 85°C. The labels of the structures are reported in the Figure together with the corresponding statistical weights and the representations of the clusters.

**Individual calculated (fitted and discrete) ECD spectra of GAG^+^(H_2_O)_30_ and (GVGVP)_2_(H_2_O)_40_ at 15°C, 30°C, 85°C and 90°C**

**Figure S5.** Individual total fitted and discrete ECD spectra of the GAG^+^(H_2_O)_30_ clusters at 30°C. The spectra reported herein are not statistically weighted. The labels of the structures are reported in the Figure and consistent with those proposed in Table 2 of the Main Article.

**Figure S6.** Individual total fitted and discrete ECD spectra of the GAG^+^(H_2_O)_30_ clusters at 90°C. The spectra reported herein are not statistically weighted. The labels of the structures are reported in the Figure and consistent with those proposed in Table 2 of the Main Article.

**Figure S7.** Individual total fitted and discrete ECD spectra of the (GVGVP)_2_(H_2_O)_40_ clusters at 15°C. The spectra reported herein are not statistically weighted. The labels of the structures are reported in the Figure and consistent with those proposed in Table 2 of the Main Article.

**Figure S8.** Individual total fitted and discrete ECD spectra of the (GVGVP)_2_(H_2_O)_40_ clusters at 85°C. The spectra reported herein are not statistically weighted. The labels of the structures are reported in the Figure and consistent with those proposed in Table 2 of the Main Article.

**Effect of the xc functional on the ECD spectra**

The choice of the xc functional (wB97X-D) was based on its ability to describe properly intra- and inter-molecular H bonds, as well as charge-transfer excitations[[1]](#endnote-2), which are very relevant to the physics of the systems selected in this work. However, the quality of this decision has been briefly tested employing a second functional, the hybrid CAM-B3LYP, for comparison as explained in the Computational Details section.

The calculations with the CAM-B3LYP were realised only on the most probable conformations at low temperature values (i.e., GAG^+^(H_2_O)_30_ at 30°C, and (GVGVP)_2_(H_2_O)_40_ at 15°C) and compared with the ECD spectra obtained for the same structures with the wB97X-D. All the calculated spectra are reported in the following Figure S9 with the experimental ECD as well.


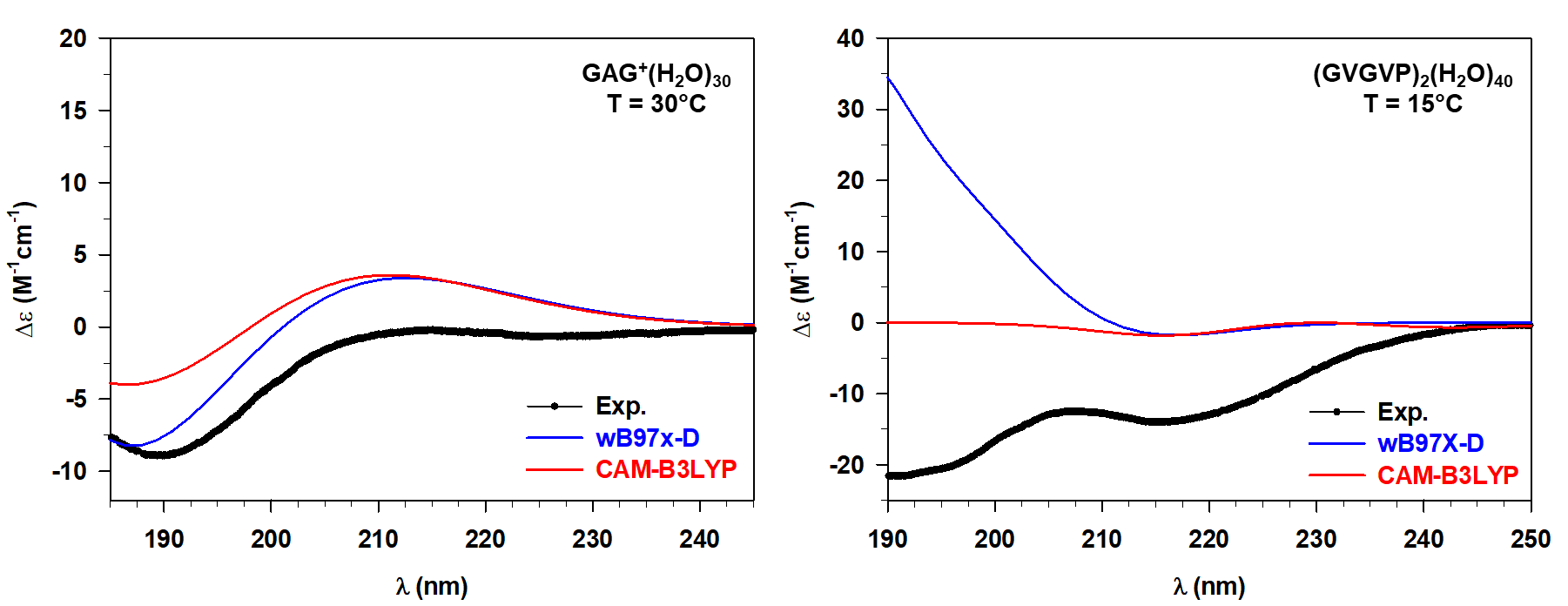


**Figure S9.** Experimental (Exp.) UV-CD and calculated UV-CD with wB97X-D and CAM-B3LYP xc functional of the most probable conformation of the solvated GAG^+^ at 30°C (left panel), and solvated (GVGVP)_2_ at 15°C (right panel). The spectra reported herein are not statistically weighted.

The left panel of Figure S9 reveals that both the calculated ECD spectra are in good quality agreement with respect to the experimental one, therefore a hybrid functional could be a valid choice as well. However, small improvements of the calculated spectrum can be noticed considering the wB97X-D functional instead of the CAM-B3LYP in terms of intensity scale and position of the minimum peak. The situation becomes more complex for the short elastin-like peptide since both the calculated ECD behaviours differ from the experimental one, as shown in the right panel of Figure S9. Because of the higher complexity of this system, it is clear that a single conformation is not able to reproduce properly all the experimental features (see also Figure S3), but a statistical approach is needed. From a very qualitative perspective, we can just notice that the shape of the experimental minimum around 218 nm is vaguely reproduced by the calculation with the wB97X-D, even though the intensity scale is different from the experimental one, while the dichroism is extremely reduced with the CAM-B3LYP, obtaining an almost null response. However, this is just a preliminary analysis and all the extracted conformers should be treated with both (or more) xc functionals before selecting the one which gives the best agreement with the experiment. Since this would result in an increase of the computational cost, we acknowledge that the calculation with only one xc functional is an approximation and eventual limitation of the approach.

**Effect of the solvent mean-field on the ECD spectra**

The effect of the solvent on the spectral features has been widely discussed in the article, in particular in Section 3.4 of the Results and Discussion. We focused there on both direct and indirect effects of the water molecules on the treated peptides, such as modification of the electronic states and chromophore geometry. Herein we want to focus on the effect of the mean-field produced by the solvent, thus on the differences obtained using an implicit solvent model instead of treating explicitly the water molecules. A similar discussion can be also found in the literature for the GAG^+^ system[[2]](#endnote-3),[[3]](#endnote-4).

For this purpose, as briefly explained in the Computational Details section, we calculated the ECD spectra of the bare GAG^+^ conformers and of the GAG^+^(H_2_O)_30_ clusters using the COSMO solvation scheme. The two new resulting statistically weighted ECD spectra have been compared with respect to the experimental one and the calculated spectrum obtained considering only the explicit water molecules. All the data are collected in the following Figure S10.

**Figure S10.** Experimental (Exp.) and statistical calculated (Calc.) ECD spectra of GAG^+^ at 30°C. The following conditions were used for the calculated spectra: (i) GAG^+^ explicitly solvated with 30 water molecules (black dashed line), (ii) GAG^+^ explicitly solvated with 30 water molecules and the COSMO solvation model (pink dashed line), (iii) GAG^+^ solvated with the COSMO solvation model (green dashed line).

The results obtained suggest a decrease of the quality agreement between the experimental and calculated ECD when the dielectric model is included. Indeed, in both cases with COSMO reported in Figure S10 (i.e., bare tripeptide, solvated tripeptide) we observe a not negligible blueshift of the main minimum point, as well as large differences in intensity considering only the tripeptide in the dielectric medium. Therefore, a change in the optical response occurs when the systems is surrounded by the dielectric medium, while a good quality agreement is obtained even considering a small number of explicit water molecules (i.e., 30). The deterioration of the results obtained with only the implicit COSMO approach is not surprising, since in present case the role of solvent hydrogen bonds is crucial for a proper description of the spectrum. On the other hand, also the hybrid implicit COSMO in presence of explicit water molecules does not perform better than the pure explicit solvent treatment. This is probably due to artifacts generated at the boundary between explicit solvent and polarizable continuum, as already pointed out by Brancato et al.[[4]](#endnote-5)

**UV- Photoabsorption Spectra**

The UV-Photoabsorption spectra have been also considered and compared with those available in the literatureError: Reference source not found, first discussing the results for GAG^+^ obtained at 30°C in Figure S11.

**Figure S11.** Experimental (Exp. – black dashed line) and calculated (Calc. – pink solid line) UV-Photoabsorption spectra of GAG^+^ in water at 30°C. Details of both the convolution and the intensity unit are reported in the Computational Details.

The comparison between the experimental and calculated results points out that a global agreement is found, due to the structureless behaviour of the photoabsorption, although some discrepancies must be taken in account. The intensity difference is particularly significant in the (185-205) nm region, then reduced moving towards lower energy values. Such discrepancy is strongly influenced by the different decreasing trend that can be observed for the two spectra. Indeed, it is possible to notice that the calculated UV-Photoabsorption spectrum shows a much steeper descent behaviour with respect to that experimentally measured. Furthermore, around 193 nm the experimental spectrum displays a weak shoulder which is not present in the calculated profile.

As previously done, it is possible to include within the analysis the variation of the spectral features as an effect of the temperature. According to our knowledge, the UV-Photoabsorption spectrum of this system has not been measured at 90°C. However, a general correlation among the temperature and the spectral features can be extracted comparing our two calculated UV-Photoabsorption spectra (i.e., at 30°C and 90°C) with those experimentally recorded at several T values (i.e., 10°C, 30°C and 70°C).

**Figure S12.** Upper panel: Experimental (Exp.) UV-Photoabsorption (Photoabs.) spectra of GAG^+^ in water taken at 10°C (blue dashed line), 30°C (black dashed line), and 70°C (orange dashed line), respectively. Lower panel: Calculated (Calc.) UV-Photoabsorption (Photoabs.) spectra of GAG^+^ in water taken at 30°C (pink solid line) and 90°C (green solid line), respectively. Details about both the convolution and the intensity unit are reported in the Computational Details.

A qualitative analysis of the two calculated spectra (lower panel of Figure S12) highlights no significant changes increasing the temperature, except for a uniform intensity increase. Actually, this difference is particularly marked at higher energy values (around 185 – 190 nm), while it tends to zero towards low energy values. Furthermore, the separation between the different experimental patterns is rapidly reduced from 195 nm onward, bringing to an overlay of the spectra. The same coincidence is achieved also for the calculated results but in a much slighter way (i.e., from 205 nm onward). Beside the limited discrepancies between the experimental and theoretical results, the latter ones describe the spectral changes occurring after the temperature increase.

**Table S1.** Atomic coordinates of the GAG^+^(H_2_O)_30_ clusters at 30°C.

| B1-A1 | x (Å) | y (Å) | z (Å) |
| --- | --- | --- | --- |
| N | 0.006 | 0.017 | 0.012 |
| C | 1.474 | 0.018 | 0.011 |
| C | 2.007 | 1.451 | 0.016 |
| N | 3.293 | 1.501 | -0.317 |
| C | 4.084 | 2.680 | -0.583 |
| C | 3.736 | 3.177 | -2.005 |
| N | 3.999 | 4.470 | -2.236 |
| C | 3.785 | 5.063 | -3.525 |
| C | 5.053 | 5.664 | -4.099 |
| O | 4.823 | 6.582 | -5.021 |
| H | -0.363 | -0.832 | -0.431 |
| H | -0.470 | 0.202 | 0.910 |
| H | -0.341 | 0.626 | -0.746 |
| H | 1.826 | -0.517 | 0.891 |
| H | 1.825 | -0.506 | -0.876 |
| O | 1.273 | 2.390 | 0.288 |
| H | 3.716 | 0.622 | -0.581 |
| H | 3.771 | 3.447 | 0.128 |
| C | 5.578 | 2.389 | -0.421 |
| H | 6.142 | 3.282 | -0.167 |
| H | 5.763 | 1.565 | 0.264 |
| H | 5.988 | 2.095 | -1.394 |
| O | 3.247 | 2.414 | -2.826 |
| H | 4.413 | 5.028 | -1.494 |
| H | 3.429 | 4.294 | -4.213 |
| H | 3.009 | 5.823 | -3.492 |
| O | 6.164 | 5.354 | -3.727 |
| H | 5.642 | 6.973 | -5.402 |
| O | 0.192 | 2.912 | -3.477 |
| H | 1.036 | 2.453 | -3.546 |
| H | -0.265 | 2.591 | -2.695 |
| O | 8.507 | 9.262 | -4.093 |
| H | 8.286 | 10.055 | -4.607 |
| H | 8.936 | 9.601 | -3.300 |
| O | 6.659 | 8.224 | -6.401 |
| H | 7.440 | 8.606 | -5.997 |
| H | 6.802 | 8.190 | -7.350 |
| O | 6.029 | -1.458 | 3.682 |
| H | 5.162 | -1.052 | 3.707 |
| H | 6.242 | -1.717 | 4.582 |
| O | -0.106 | 4.858 | -5.433 |
| H | 0.683 | 5.406 | -5.405 |
| H | -0.094 | 4.222 | -4.709 |
| O | 8.794 | 4.472 | -3.364 |
| H | 7.928 | 4.883 | -3.480 |
| H | 8.683 | 3.558 | -3.631 |
| O | 3.756 | 0.118 | 2.978 |
| H | 3.407 | -0.525 | 3.607 |
| H | 3.825 | 0.944 | 3.467 |
| O | -3.818 | -2.844 | 1.872 |
| H | -4.456 | -2.130 | 1.820 |
| H | -4.080 | -3.412 | 2.602 |
| O | 7.933 | 0.485 | 2.262 |
| H | 7.142 | -0.027 | 2.465 |
| H | 8.135 | 0.371 | 1.332 |
| O | 3.145 | -2.662 | 1.842 |
| H | 3.509 | -1.771 | 1.911 |
| H | 3.830 | -3.278 | 1.575 |
| O | 3.977 | -4.254 | 4.461 |
| H | 4.802 | -4.746 | 4.492 |
| H | 3.881 | -3.919 | 3.565 |
| O | 3.888 | 8.158 | -7.588 |
| H | 2.975 | 8.452 | -7.551 |
| H | 4.421 | 8.720 | -7.021 |
| O | 8.414 | 11.345 | -6.013 |
| H | 8.634 | 11.020 | -6.893 |
| H | 8.633 | 12.282 | -6.035 |
| O | 0.539 | 5.077 | -0.530 |
| H | 0.788 | 4.145 | -0.539 |
| H | 1.155 | 5.471 | 0.092 |
| O | -1.614 | 1.687 | -1.604 |
| H | -2.151 | 1.089 | -2.135 |
| H | -2.251 | 2.213 | -1.109 |
| O | -1.345 | -2.316 | 0.135 |
| H | -0.623 | -2.961 | 0.082 |
| H | -2.088 | -2.719 | 0.604 |
| O | 4.671 | -0.950 | -0.621 |
| H | 4.602 | -1.905 | -0.561 |
| H | 5.616 | -0.771 | -0.647 |
| O | 9.237 | 6.026 | -1.089 |
| H | 8.856 | 6.859 | -1.385 |
| H | 9.267 | 5.424 | -1.842 |
| O | 2.072 | 6.871 | -5.396 |
| H | 1.715 | 7.333 | -4.635 |
| H | 3.015 | 7.055 | -5.467 |
| O | 7.375 | 7.619 | -2.474 |
| H | 6.948 | 6.825 | -2.813 |
| H | 7.635 | 8.114 | -3.261 |
| O | 10.186 | 8.985 | -6.316 |
| H | 11.005 | 8.530 | -6.117 |
| H | 9.661 | 9.063 | -5.511 |
| O | 10.242 | 2.601 | -0.333 |
| H | 10.396 | 3.547 | -0.227 |
| H | 9.860 | 2.575 | -1.214 |
| O | 5.047 | 5.978 | -0.040 |
| H | 4.988 | 6.926 | -0.197 |
| H | 5.947 | 5.863 | 0.286 |
| O | 0.848 | -3.887 | 0.006 |
| H | 0.666 | -4.768 | 0.338 |
| H | 1.742 | -3.644 | 0.261 |
| O | -0.875 | -6.883 | 5.377 |
| H | -1.427 | -7.358 | 4.754 |
| H | -0.005 | -6.693 | 5.008 |
| O | -2.678 | -4.811 | 5.483 |
| H | -1.934 | -5.432 | 5.385 |
| H | -3.381 | -5.441 | 5.666 |
| O | 1.230 | -4.064 | 6.137 |
| H | 0.410 | -3.571 | 6.067 |
| H | 1.271 | -4.616 | 5.351 |
| O | 1.629 | -5.946 | 3.979 |
| H | 2.472 | -5.506 | 3.797 |
| H | 1.401 | -6.452 | 3.197 |
| O | -1.142 | 0.224 | 2.259 |
| H | -0.825 | 0.783 | 2.973 |
| H | -1.841 | -0.339 | 2.602 |
| O | 6.067 | 11.082 | -4.491 |
| H | 6.802 | 11.323 | -5.066 |
| H | 5.284 | 11.332 | -4.986 |
|  |  |  |  |
| B1-B1 |  |  |  |
| N | 0.000 | -0.008 | -0.001 |
| C | 1.464 | -0.014 | -0.003 |
| C | 2.017 | 1.399 | -0.004 |
| N | 3.306 | 1.489 | 0.371 |
| C | 4.050 | 2.742 | 0.334 |
| C | 5.487 | 2.496 | -0.168 |
| N | 5.885 | 1.209 | -0.252 |
| C | 7.169 | 0.741 | -0.682 |
| C | 7.098 | 0.036 | -2.023 |
| O | 7.998 | -0.942 | -2.088 |
| H | -0.381 | 0.402 | 0.879 |
| H | -0.404 | 0.426 | -0.826 |
| H | -0.373 | -0.957 | 0.095 |
| H | 1.796 | -0.617 | 0.838 |
| H | 1.818 | -0.537 | -0.892 |
| O | 1.325 | 2.327 | -0.382 |
| H | 3.729 | 0.656 | 0.741 |
| H | 3.560 | 3.364 | -0.415 |
| C | 4.092 | 3.474 | 1.670 |
| H | 3.125 | 3.876 | 1.954 |
| H | 4.477 | 2.798 | 2.439 |
| H | 4.864 | 4.243 | 1.620 |
| O | 6.207 | 3.445 | -0.419 |
| H | 5.242 | 0.470 | -0.042 |
| H | 7.838 | 1.594 | -0.823 |
| H | 7.605 | 0.075 | 0.062 |
| O | 6.393 | 0.369 | -2.946 |
| H | 8.116 | -1.199 | -3.018 |
| O | -0.848 | -1.841 | -2.317 |
| H | -1.734 | -1.964 | -1.967 |
| H | -0.999 | -1.626 | -3.245 |
| O | 1.986 | -3.053 | 0.106 |
| H | 2.553 | -3.036 | -0.673 |
| H | 1.175 | -3.511 | -0.122 |
| O | -3.622 | 2.132 | 2.505 |
| H | -4.034 | 2.825 | 1.985 |
| H | -3.940 | 1.270 | 2.214 |
| O | 4.080 | -4.033 | -2.721 |
| H | 4.789 | -3.995 | -3.378 |
| H | 3.419 | -3.405 | -3.015 |
| O | -1.765 | -1.660 | 0.959 |
| H | -1.890 | -2.611 | 0.909 |
| H | -2.581 | -1.272 | 1.309 |
| O | 10.247 | 2.051 | -2.344 |
| H | 10.535 | 2.054 | -1.428 |
| H | 10.981 | 1.787 | -2.917 |
| O | 0.671 | 3.798 | 3.136 |
| H | 0.230 | 3.904 | 3.984 |
| H | 1.091 | 2.923 | 3.184 |
| O | -1.354 | 4.014 | 1.107 |
| H | -0.769 | 4.205 | 0.364 |
| H | -0.776 | 3.747 | 1.842 |
| O | 7.482 | 6.466 | 1.237 |
| H | 7.816 | 5.596 | 1.002 |
| H | 8.093 | 7.109 | 0.876 |
| O | 10.707 | 2.021 | 0.375 |
| H | 10.237 | 1.534 | 1.061 |
| H | 11.491 | 2.397 | 0.790 |
| O | 1.124 | 6.505 | 2.170 |
| H | 0.932 | 5.570 | 2.313 |
| H | 1.646 | 6.777 | 2.928 |
| O | -3.487 | 5.662 | 2.471 |
| H | -2.894 | 5.756 | 3.217 |
| H | -3.023 | 5.120 | 1.824 |
| O | -2.668 | 2.312 | -0.610 |
| H | -3.617 | 2.369 | -0.489 |
| H | -2.272 | 2.922 | 0.037 |
| O | 8.282 | 4.363 | -2.147 |
| H | 8.707 | 3.612 | -2.579 |
| H | 7.488 | 4.012 | -1.721 |
| O | -1.008 | 1.018 | 2.310 |
| H | -0.987 | 0.614 | 3.180 |
| H | -1.735 | 1.670 | 2.318 |
| O | -4.178 | -0.384 | 1.167 |
| H | -4.348 | -0.236 | 0.232 |
| H | -4.991 | -0.755 | 1.527 |
| O | 1.890 | -2.523 | -2.899 |
| H | 2.383 | -1.742 | -3.204 |
| H | 1.003 | -2.229 | -2.671 |
| O | 9.828 | -3.325 | -3.945 |
| H | 9.521 | -2.453 | -4.196 |
| H | 9.873 | -3.851 | -4.746 |
| O | 7.483 | -1.595 | -4.895 |
| H | 7.835 | -1.992 | -5.695 |
| H | 7.498 | -0.634 | -4.967 |
| O | 4.629 | -1.171 | 0.960 |
| H | 5.012 | -2.053 | 0.877 |
| H | 4.145 | -1.263 | 1.786 |
| O | 7.588 | 3.332 | 3.222 |
| H | 6.807 | 3.763 | 3.572 |
| H | 7.930 | 3.860 | 2.492 |
| O | 3.197 | -0.246 | -3.405 |
| H | 2.810 | 0.622 | -3.509 |
| H | 4.160 | -0.250 | -3.401 |
| O | 2.545 | 6.225 | -0.229 |
| H | 3.497 | 6.135 | -0.163 |
| H | 2.208 | 6.456 | 0.649 |
| O | 12.433 | 1.317 | -4.071 |
| H | 13.050 | 2.001 | -3.804 |
| H | 12.950 | 0.544 | -4.311 |
| O | 9.428 | 4.508 | 0.692 |
| H | 9.760 | 3.609 | 0.611 |
| H | 9.112 | 4.770 | -0.189 |
| O | 12.664 | -2.039 | -2.388 |
| H | 13.270 | -2.767 | -2.542 |
| H | 12.009 | -2.389 | -1.779 |
| O | 10.932 | -0.934 | 0.472 |
| H | 10.126 | -1.410 | 0.263 |
| H | 10.866 | -0.063 | 0.070 |
| O | 1.991 | 1.365 | 3.584 |
| H | 2.095 | 1.167 | 4.519 |
| H | 2.487 | 0.686 | 3.124 |
| O | 6.052 | -3.933 | -4.549 |
| H | 6.183 | -4.227 | -5.451 |
| H | 6.525 | -3.098 | -4.458 |
| O | -0.226 | -4.419 | -0.468 |
| H | 0.277 | -4.798 | -1.200 |
| H | -1.118 | -4.726 | -0.647 |
|  |  |  |  |
| B1-B2 |  |  |  |
| N | 0.007 | 0.001 | -0.005 |
| C | 1.476 | 0.000 | -0.004 |
| C | 2.043 | 1.410 | -0.007 |
| N | 3.320 | 1.486 | 0.367 |
| C | 4.112 | 2.709 | 0.345 |
| C | 5.534 | 2.385 | -0.151 |
| N | 5.859 | 1.081 | -0.235 |
| C | 7.138 | 0.604 | -0.669 |
| C | 6.942 | -0.056 | -2.027 |
| O | 7.747 | -1.074 | -2.244 |
| H | -0.344 | 0.420 | 0.894 |
| H | -0.408 | 0.428 | -0.822 |
| H | -0.268 | -0.987 | 0.096 |
| H | 1.786 | -0.570 | 0.868 |
| H | 1.841 | -0.520 | -0.887 |
| O | 1.376 | 2.320 | -0.482 |
| H | 3.699 | 0.690 | 0.850 |
| H | 3.656 | 3.382 | -0.378 |
| C | 4.229 | 3.371 | 1.705 |
| H | 3.303 | 3.801 | 2.068 |
| H | 4.616 | 2.644 | 2.428 |
| H | 5.027 | 4.107 | 1.649 |
| O | 6.317 | 3.291 | -0.366 |
| H | 5.168 | 0.360 | -0.116 |
| H | 7.810 | 1.450 | -0.813 |
| H | 7.575 | -0.099 | 0.038 |
| O | 6.177 | 0.377 | -2.870 |
| H | 7.733 | -1.252 | -3.218 |
| O | 2.193 | -2.595 | -2.699 |
| H | 1.638 | -2.401 | -3.455 |
| H | 2.780 | -1.839 | -2.508 |
| O | 6.982 | 5.110 | 2.827 |
| H | 7.871 | 4.793 | 2.575 |
| H | 7.122 | 5.599 | 3.644 |
| O | -0.601 | 1.422 | 2.282 |
| H | -0.710 | 1.543 | 3.230 |
| H | -0.568 | 2.337 | 1.937 |
| O | -2.344 | 3.406 | 3.229 |
| H | -2.863 | 2.833 | 2.644 |
| H | -1.776 | 3.941 | 2.666 |
| O | 4.496 | -1.272 | 0.358 |
| H | 4.071 | -1.560 | 1.168 |
| H | 4.483 | -2.031 | -0.254 |
| O | 9.490 | 3.927 | 2.227 |
| H | 9.618 | 3.063 | 1.793 |
| H | 10.279 | 4.177 | 2.710 |
| O | 3.955 | 5.910 | 3.467 |
| H | 3.792 | 6.668 | 2.899 |
| H | 4.787 | 5.553 | 3.138 |
| O | 3.399 | -0.163 | -2.613 |
| H | 2.922 | 0.603 | -2.936 |
| H | 4.359 | -0.027 | -2.724 |
| O | -0.028 | 4.044 | 1.444 |
| H | 0.259 | 4.082 | 0.530 |
| H | 0.706 | 4.016 | 2.075 |
| O | 1.614 | 3.533 | 3.797 |
| H | 2.106 | 2.721 | 3.905 |
| H | 1.804 | 4.120 | 4.529 |
| O | 10.257 | -2.679 | -3.737 |
| H | 9.731 | -3.037 | -3.009 |
| H | 11.127 | -2.493 | -3.367 |
| O | 10.397 | 5.148 | -0.526 |
| H | 10.840 | 5.958 | -0.262 |
| H | 10.200 | 4.702 | 0.311 |
| O | 4.045 | -3.727 | -1.284 |
| H | 4.368 | -4.538 | -1.683 |
| H | 3.295 | -3.509 | -1.852 |
| O | -1.154 | -2.679 | -2.682 |
| H | -1.957 | -2.137 | -2.602 |
| H | -1.356 | -3.266 | -3.420 |
| O | 0.072 | -2.786 | 0.276 |
| H | -0.339 | -3.177 | -0.507 |
| H | 0.419 | -3.519 | 0.791 |
| O | 8.080 | 4.431 | -2.304 |
| H | 8.998 | 4.430 | -2.021 |
| H | 7.578 | 4.047 | -1.573 |
| O | -3.264 | -1.071 | -1.280 |
| H | -4.058 | -1.387 | -1.722 |
| H | -3.238 | -1.630 | -0.480 |
| O | 10.998 | 1.177 | -1.534 |
| H | 11.489 | 1.961 | -1.853 |
| H | 10.641 | 0.745 | -2.336 |
| O | -3.842 | 1.737 | 1.751 |
| H | -4.663 | 2.232 | 1.693 |
| H | -3.449 | 1.776 | 0.868 |
| O | 10.243 | 0.043 | -3.851 |
| H | 9.947 | 0.340 | -4.711 |
| H | 10.121 | -0.920 | -3.769 |
| O | 10.212 | 1.657 | 0.946 |
| H | 10.231 | 1.566 | -0.030 |
| H | 10.234 | 0.806 | 1.386 |
| O | 12.298 | 3.577 | -2.236 |
| H | 13.225 | 3.810 | -2.278 |
| H | 11.788 | 4.371 | -2.037 |
| O | 6.133 | 6.747 | 0.715 |
| H | 6.851 | 7.039 | 0.150 |
| H | 6.528 | 6.341 | 1.498 |
| O | 1.824 | -5.429 | -2.488 |
| H | 1.739 | -4.492 | -2.693 |
| H | 1.758 | -5.886 | -3.329 |
| O | -3.304 | -3.055 | 0.733 |
| H | -2.899 | -3.704 | 0.149 |
| H | -4.213 | -3.357 | 0.828 |
| O | 7.846 | -4.273 | -1.817 |
| H | 8.130 | -5.190 | -1.770 |
| H | 7.015 | -4.293 | -2.298 |
| O | 12.517 | 3.023 | 0.921 |
| H | 13.353 | 2.604 | 0.713 |
| H | 11.828 | 2.390 | 0.695 |
| O | 11.748 | -1.330 | -0.623 |
| H | 11.704 | -0.420 | -0.968 |
| H | 12.663 | -1.559 | -0.813 |
| O | 7.434 | -1.673 | -4.846 |
| H | 6.876 | -1.069 | -5.345 |
| H | 8.229 | -1.860 | -5.355 |
| O | -2.154 | 2.346 | -0.622 |
| H | -2.357 | 3.258 | -0.857 |
| H | -1.842 | 2.006 | -1.466 |
|  |  |  |  |
| B1-B3 |  |  |  |
| N | 0.015 | 0.000 | -0.008 |
| C | 1.483 | 0.000 | -0.009 |
| C | 2.032 | 1.428 | -0.009 |
| N | 3.321 | 1.521 | 0.368 |
| C | 4.109 | 2.748 | 0.353 |
| C | 5.539 | 2.426 | -0.147 |
| N | 5.848 | 1.136 | -0.230 |
| C | 7.091 | 0.554 | -0.651 |
| C | 6.918 | -0.137 | -1.993 |
| O | 7.707 | -1.206 | -2.091 |
| H | -0.349 | 0.415 | 0.889 |
| H | -0.401 | 0.430 | -0.828 |
| H | -0.313 | -0.972 | 0.090 |
| H | 1.810 | -0.562 | 0.865 |
| H | 1.857 | -0.507 | -0.898 |
| O | 1.349 | 2.334 | -0.453 |
| H | 3.635 | 0.803 | 0.998 |
| H | 3.653 | 3.407 | -0.386 |
| C | 4.163 | 3.426 | 1.716 |
| H | 3.206 | 3.845 | 2.012 |
| H | 4.523 | 2.706 | 2.459 |
| H | 4.953 | 4.173 | 1.712 |
| O | 6.335 | 3.331 | -0.386 |
| H | 5.119 | 0.446 | -0.133 |
| H | 7.845 | 1.331 | -0.779 |
| H | 7.449 | -0.159 | 0.089 |
| O | 6.230 | 0.275 | -2.892 |
| H | 7.766 | -1.460 | -3.037 |
| O | -0.577 | -1.878 | -2.312 |
| H | -1.353 | -2.320 | -1.956 |
| H | -0.716 | -1.853 | -3.265 |
| O | 5.469 | -3.698 | -0.944 |
| H | 5.066 | -4.492 | -0.595 |
| H | 5.314 | -3.649 | -1.894 |
| O | -3.360 | 1.784 | 2.423 |
| H | -3.894 | 2.113 | 1.696 |
| H | -3.880 | 2.010 | 3.202 |
| O | 2.087 | -1.885 | -3.421 |
| H | 1.938 | -1.585 | -4.321 |
| H | 1.228 | -2.017 | -3.006 |
| O | 4.555 | -1.220 | 0.278 |
| H | 4.214 | -1.304 | 1.169 |
| H | 4.853 | -2.087 | -0.068 |
| O | 10.642 | -1.168 | -3.176 |
| H | 9.765 | -1.321 | -3.531 |
| H | 10.850 | -1.989 | -2.721 |
| O | 0.408 | 3.671 | 2.918 |
| H | -0.022 | 3.744 | 3.775 |
| H | -0.276 | 3.912 | 2.258 |
| O | -3.160 | 5.292 | 2.703 |
| H | -2.674 | 5.486 | 3.502 |
| H | -2.637 | 4.833 | 2.041 |
| O | 9.344 | 4.742 | 0.674 |
| H | 8.750 | 5.483 | 0.539 |
| H | 8.756 | 4.013 | 0.902 |
| O | 13.390 | 3.940 | -1.107 |
| H | 12.596 | 4.390 | -1.410 |
| H | 13.190 | 3.652 | -0.215 |
| O | 8.081 | 2.248 | 1.974 |
| H | 8.941 | 1.958 | 1.626 |
| H | 7.831 | 1.527 | 2.555 |
| O | 1.696 | 6.439 | 1.927 |
| H | 1.371 | 5.619 | 2.309 |
| H | 2.211 | 6.872 | 2.633 |
| O | -1.412 | 4.175 | 0.987 |
| H | -0.892 | 4.794 | 0.461 |
| H | -1.584 | 3.378 | 0.468 |
| O | 8.186 | 3.951 | -2.549 |
| H | 7.639 | 3.761 | -3.314 |
| H | 7.693 | 3.573 | -1.809 |
| O | -0.791 | 1.234 | 2.159 |
| H | -0.183 | 1.909 | 2.504 |
| H | -1.694 | 1.587 | 2.272 |
| O | 4.176 | -4.009 | -3.268 |
| H | 3.566 | -3.255 | -3.312 |
| H | 3.762 | -4.735 | -3.737 |
| O | -0.726 | -2.435 | 0.837 |
| H | -0.136 | -3.179 | 0.627 |
| H | -1.346 | -2.738 | 1.504 |
| O | 5.501 | 7.010 | 2.219 |
| H | 5.266 | 6.597 | 1.366 |
| H | 5.985 | 6.342 | 2.710 |
| O | 3.437 | 7.724 | 3.727 |
| H | 3.599 | 7.508 | 4.644 |
| H | 4.231 | 7.611 | 3.175 |
| O | 7.913 | -1.945 | -4.748 |
| H | 7.739 | -1.076 | -5.131 |
| H | 7.125 | -2.456 | -4.962 |
| O | 5.460 | 5.853 | -0.472 |
| H | 6.218 | 6.443 | -0.432 |
| H | 5.839 | 4.954 | -0.446 |
| O | -2.179 | 1.867 | -1.083 |
| H | -2.989 | 1.380 | -1.268 |
| H | -1.941 | 2.211 | -1.951 |
| O | 0.294 | -4.577 | -0.338 |
| H | -0.477 | -4.558 | -0.910 |
| H | 0.928 | -5.158 | -0.765 |
| O | 12.878 | 1.077 | -2.346 |
| H | 12.495 | 0.639 | -1.584 |
| H | 13.587 | 1.603 | -1.955 |
| O | 14.300 | -0.818 | -4.071 |
| H | 13.679 | -1.158 | -4.716 |
| H | 13.755 | -0.365 | -3.410 |
| O | 10.551 | -3.450 | -5.254 |
| H | 9.699 | -3.161 | -5.591 |
| H | 10.983 | -2.628 | -4.999 |
| O | 1.992 | 1.132 | 3.719 |
| H | 1.833 | 2.012 | 3.360 |
| H | 1.139 | 0.725 | 3.886 |
| O | -6.443 | 2.259 | 2.634 |
| H | -6.307 | 1.347 | 2.889 |
| H | -6.061 | 2.414 | 1.770 |
| O | -5.679 | -0.894 | 0.609 |
| H | -6.612 | -0.941 | 0.383 |
| H | -5.674 | -0.720 | 1.553 |
| O | 10.671 | 1.528 | 1.036 |
| H | 10.834 | 0.755 | 0.494 |
| H | 11.487 | 2.027 | 1.094 |
|  |  |  |  |
| B1-C1 |  |  |  |
| N | 0.015 | 0.000 | -0.008 |
| C | 1.483 | 0.000 | -0.009 |
| C | 2.032 | 1.428 | -0.009 |
| N | 3.321 | 1.521 | 0.368 |
| C | 4.109 | 2.748 | 0.353 |
| C | 5.539 | 2.426 | -0.147 |
| N | 5.848 | 1.136 | -0.230 |
| C | 7.091 | 0.554 | -0.651 |
| C | 6.918 | -0.137 | -1.993 |
| O | 7.707 | -1.206 | -2.091 |
| H | -0.349 | 0.415 | 0.889 |
| H | -0.401 | 0.430 | -0.828 |
| H | -0.313 | -0.972 | 0.090 |
| H | 1.810 | -0.562 | 0.865 |
| H | 1.857 | -0.507 | -0.898 |
| O | 1.349 | 2.334 | -0.453 |
| H | 3.635 | 0.803 | 0.998 |
| H | 3.653 | 3.407 | -0.386 |
| C | 4.163 | 3.426 | 1.716 |
| H | 3.206 | 3.845 | 2.012 |
| H | 4.523 | 2.706 | 2.459 |
| H | 4.953 | 4.173 | 1.712 |
| O | 6.335 | 3.331 | -0.386 |
| H | 5.119 | 0.446 | -0.133 |
| H | 7.845 | 1.331 | -0.779 |
| H | 7.449 | -0.159 | 0.089 |
| O | 6.230 | 0.275 | -2.892 |
| H | 7.766 | -1.460 | -3.037 |
| O | -0.577 | -1.878 | -2.312 |
| H | -1.353 | -2.320 | -1.956 |
| H | -0.716 | -1.853 | -3.265 |
| O | 5.469 | -3.698 | -0.944 |
| H | 5.066 | -4.492 | -0.595 |
| H | 5.314 | -3.649 | -1.894 |
| O | -3.360 | 1.784 | 2.423 |
| H | -3.894 | 2.113 | 1.696 |
| H | -3.880 | 2.010 | 3.202 |
| O | 2.087 | -1.885 | -3.421 |
| H | 1.938 | -1.585 | -4.321 |
| H | 1.228 | -2.017 | -3.006 |
| O | 4.555 | -1.220 | 0.278 |
| H | 4.214 | -1.304 | 1.169 |
| H | 4.853 | -2.087 | -0.068 |
| O | 10.642 | -1.168 | -3.176 |
| H | 9.765 | -1.321 | -3.531 |
| H | 10.850 | -1.989 | -2.721 |
| O | 0.408 | 3.671 | 2.918 |
| H | -0.022 | 3.744 | 3.775 |
| H | -0.276 | 3.912 | 2.258 |
| O | -3.160 | 5.292 | 2.703 |
| H | -2.674 | 5.486 | 3.502 |
| H | -2.637 | 4.833 | 2.041 |
| O | 9.344 | 4.742 | 0.674 |
| H | 8.750 | 5.483 | 0.539 |
| H | 8.756 | 4.013 | 0.902 |
| O | 13.390 | 3.940 | -1.107 |
| H | 12.596 | 4.390 | -1.410 |
| H | 13.190 | 3.652 | -0.215 |
| O | 8.081 | 2.248 | 1.974 |
| H | 8.941 | 1.958 | 1.626 |
| H | 7.831 | 1.527 | 2.555 |
| O | 1.696 | 6.439 | 1.927 |
| H | 1.371 | 5.619 | 2.309 |
| H | 2.211 | 6.872 | 2.633 |
| O | -1.412 | 4.175 | 0.987 |
| H | -0.892 | 4.794 | 0.461 |
| H | -1.584 | 3.378 | 0.468 |
| O | 8.186 | 3.951 | -2.549 |
| H | 7.639 | 3.761 | -3.314 |
| H | 7.693 | 3.573 | -1.809 |
| O | -0.791 | 1.234 | 2.159 |
| H | -0.183 | 1.909 | 2.504 |
| H | -1.694 | 1.587 | 2.272 |
| O | 4.176 | -4.009 | -3.268 |
| H | 3.566 | -3.255 | -3.312 |
| H | 3.762 | -4.735 | -3.737 |
| O | -0.726 | -2.435 | 0.837 |
| H | -0.136 | -3.179 | 0.627 |
| H | -1.346 | -2.738 | 1.504 |
| O | 5.501 | 7.010 | 2.219 |
| H | 5.266 | 6.597 | 1.366 |
| H | 5.985 | 6.342 | 2.710 |
| O | 3.437 | 7.724 | 3.727 |
| H | 3.599 | 7.508 | 4.644 |
| H | 4.231 | 7.611 | 3.175 |
| O | 7.913 | -1.945 | -4.748 |
| H | 7.739 | -1.076 | -5.131 |
| H | 7.125 | -2.456 | -4.962 |
| O | 5.460 | 5.853 | -0.472 |
| H | 6.218 | 6.443 | -0.432 |
| H | 5.839 | 4.954 | -0.446 |
| O | -2.179 | 1.867 | -1.083 |
| H | -2.989 | 1.380 | -1.268 |
| H | -1.941 | 2.211 | -1.951 |
| O | 0.294 | -4.577 | -0.338 |
| H | -0.477 | -4.558 | -0.910 |
| H | 0.928 | -5.158 | -0.765 |
| O | 12.878 | 1.077 | -2.346 |
| H | 12.495 | 0.639 | -1.584 |
| H | 13.587 | 1.603 | -1.955 |
| O | 14.300 | -0.818 | -4.071 |
| H | 13.679 | -1.158 | -4.716 |
| H | 13.755 | -0.365 | -3.410 |
| O | 10.551 | -3.450 | -5.254 |
| H | 9.699 | -3.161 | -5.591 |
| H | 10.983 | -2.628 | -4.999 |
| O | 1.992 | 1.132 | 3.719 |
| H | 1.833 | 2.012 | 3.360 |
| H | 1.139 | 0.725 | 3.886 |
| O | -6.443 | 2.259 | 2.634 |
| H | -6.307 | 1.347 | 2.889 |
| H | -6.061 | 2.414 | 1.770 |
| O | -5.679 | -0.894 | 0.609 |
| H | -6.612 | -0.941 | 0.383 |
| H | -5.674 | -0.720 | 1.553 |
| O | 10.671 | 1.528 | 1.036 |
| H | 10.834 | 0.755 | 0.494 |
| H | 11.487 | 2.027 | 1.094 |
|  |  |  |  |
| B1-C2 |  |  |  |
| N | -0.132 | 0.059 | 0.023 |
| C | 1.344 | 0.066 | 0.004 |
| C | 1.873 | 1.502 | 0.006 |
| N | 3.123 | 1.610 | -0.459 |
| C | 3.789 | 2.818 | -0.893 |
| C | 3.558 | 3.102 | -2.382 |
| N | 3.714 | 4.379 | -2.774 |
| C | 3.602 | 4.725 | -4.165 |
| C | 4.980 | 4.996 | -4.774 |
| O | 4.886 | 5.562 | -5.968 |
| H | -0.496 | 0.472 | -0.845 |
| H | -0.513 | -0.890 | 0.270 |
| H | -0.385 | 0.766 | 0.716 |
| H | 1.710 | -0.458 | 0.889 |
| H | 1.698 | -0.468 | -0.875 |
| O | 1.161 | 2.423 | 0.383 |
| H | 3.494 | 0.779 | -0.923 |
| H | 3.445 | 3.668 | -0.303 |
| C | 5.299 | 2.645 | -0.673 |
| H | 5.606 | 1.708 | -1.146 |
| H | 5.903 | 3.469 | -1.033 |
| H | 5.474 | 2.400 | 0.376 |
| O | 3.310 | 2.206 | -3.179 |
| H | 3.898 | 5.096 | -2.096 |
| H | 3.158 | 3.881 | -4.689 |
| H | 2.959 | 5.596 | -4.296 |
| O | 6.004 | 4.702 | -4.215 |
| H | 5.791 | 5.817 | -6.295 |
| O | 6.947 | 8.707 | -5.433 |
| H | 7.450 | 9.095 | -4.706 |
| H | 6.164 | 9.234 | -5.631 |
| O | -0.354 | 4.445 | -3.612 |
| H | -0.166 | 3.790 | -4.287 |
| H | -0.627 | 5.221 | -4.110 |
| O | 3.253 | -1.569 | 2.702 |
| H | 4.196 | -1.323 | 2.746 |
| H | 3.218 | -2.461 | 2.352 |
| O | -0.791 | -2.523 | 0.616 |
| H | -1.658 | -2.967 | 0.565 |
| H | -0.432 | -2.846 | 1.470 |
| O | 6.642 | 6.535 | -0.563 |
| H | 6.846 | 5.844 | -1.199 |
| H | 5.688 | 6.625 | -0.572 |
| O | 7.042 | 10.614 | -8.015 |
| H | 6.471 | 9.868 | -7.807 |
| H | 7.282 | 10.498 | -8.940 |
| O | -0.990 | 0.960 | -2.355 |
| H | -0.413 | 0.884 | -3.125 |
| H | -1.835 | 1.276 | -2.683 |
| O | 4.923 | 10.334 | -5.804 |
| H | 4.166 | 10.371 | -5.218 |
| H | 4.680 | 10.564 | -6.700 |
| O | -3.354 | -3.378 | 0.387 |
| H | -3.901 | -2.638 | 0.115 |
| H | -3.696 | -4.182 | -0.011 |
| O | -0.130 | 4.708 | -0.804 |
| H | -0.283 | 4.614 | -1.749 |
| H | 0.314 | 3.914 | -0.484 |
| O | -4.434 | -0.958 | -0.095 |
| H | -5.034 | -0.207 | -0.129 |
| H | -4.023 | -0.997 | -0.964 |
| O | 5.993 | -0.794 | 2.844 |
| H | 6.018 | 0.129 | 3.111 |
| H | 6.521 | -1.261 | 3.498 |
| O | 7.882 | 9.994 | -3.117 |
| H | 7.063 | 10.125 | -2.633 |
| H | 8.070 | 10.810 | -3.593 |
| O | 2.397 | -3.516 | 4.796 |
| H | 2.421 | -3.173 | 3.904 |
| H | 1.848 | -2.892 | 5.275 |
| O | 4.090 | -0.438 | -2.225 |
| H | 3.444 | -0.363 | -2.930 |
| H | 4.311 | -1.352 | -2.037 |
| O | 1.055 | 6.638 | -6.203 |
| H | 1.409 | 5.825 | -6.562 |
| H | 0.112 | 6.722 | -6.342 |
| O | -2.887 | -5.923 | 2.656 |
| H | -2.169 | -5.548 | 3.176 |
| H | -3.560 | -5.239 | 2.669 |
| O | -1.547 | -5.027 | 5.216 |
| H | -1.107 | -5.670 | 5.778 |
| H | -1.657 | -4.243 | 5.761 |
| O | 0.039 | -3.255 | 3.109 |
| H | -0.185 | -2.481 | 3.661 |
| H | -0.003 | -4.024 | 3.688 |
| O | 1.416 | 0.128 | 4.375 |
| H | 1.443 | 0.956 | 3.889 |
| H | 1.901 | -0.521 | 3.842 |
| O | 9.969 | 8.634 | -1.979 |
| H | 9.649 | 7.736 | -1.845 |
| H | 9.211 | 9.119 | -2.319 |
| O | 7.418 | 6.199 | -6.787 |
| H | 7.407 | 7.081 | -6.363 |
| H | 7.901 | 6.307 | -7.610 |
| O | 1.519 | 0.196 | -3.512 |
| H | 1.574 | 1.137 | -3.727 |
| H | 1.968 | -0.216 | -4.258 |
| O | 3.683 | 7.773 | -7.664 |
| H | 3.350 | 7.531 | -6.795 |
| H | 3.384 | 8.679 | -7.769 |
| O | 9.453 | 9.256 | -8.024 |
| H | 8.770 | 9.808 | -7.629 |
| H | 9.979 | 9.781 | -8.632 |
| O | 9.975 | 5.849 | -2.878 |
| H | 10.778 | 6.180 | -3.289 |
| H | 9.419 | 5.587 | -3.617 |
| O | -1.226 | -1.281 | 4.346 |
| H | -0.613 | -0.537 | 4.280 |
| H | -1.828 | -1.236 | 3.583 |
| O | -1.042 | 1.253 | 2.159 |
| H | -1.519 | 1.951 | 1.696 |
| H | -1.161 | 1.450 | 3.092 |
| O | -3.226 | -0.851 | 2.585 |
| H | -4.044 | -1.174 | 2.973 |
| H | -3.402 | -0.828 | 1.637 |
| O | 6.968 | 12.652 | -6.387 |
| H | 6.319 | 13.243 | -6.771 |
| H | 7.115 | 11.952 | -7.029 |
|  |  |  |  |
| B2-D1 |  |  |  |
| N | 0.004 | -0.007 | 0.010 |
| C | 1.482 | -0.006 | 0.009 |
| C | 1.944 | 1.470 | 0.011 |
| N | 3.267 | 1.649 | -0.039 |
| C | 3.965 | 2.896 | -0.319 |
| C | 3.516 | 3.603 | -1.616 |
| N | 3.821 | 4.922 | -1.604 |
| C | 3.434 | 5.850 | -2.640 |
| C | 2.868 | 7.131 | -2.065 |
| O | 2.163 | 7.836 | -2.965 |
| H | -0.309 | 0.377 | 0.908 |
| H | -0.371 | 0.523 | -0.778 |
| H | -0.376 | -0.961 | -0.023 |
| H | 1.842 | -0.572 | 0.864 |
| H | 1.832 | -0.489 | -0.900 |
| O | 1.106 | 2.353 | -0.026 |
| H | 3.844 | 0.824 | 0.054 |
| H | 3.772 | 3.588 | 0.505 |
| C | 5.460 | 2.598 | -0.401 |
| H | 5.852 | 2.168 | 0.519 |
| H | 5.628 | 1.878 | -1.207 |
| H | 6.013 | 3.482 | -0.707 |
| O | 2.984 | 3.014 | -2.539 |
| H | 4.147 | 5.335 | -0.732 |
| H | 4.272 | 6.116 | -3.294 |
| H | 2.676 | 5.386 | -3.272 |
| O | 2.898 | 7.433 | -0.901 |
| H | 1.861 | 8.636 | -2.498 |
| O | 5.615 | 8.514 | -4.689 |
| H | 5.234 | 9.142 | -4.064 |
| H | 6.306 | 8.001 | -4.253 |
| O | -2.418 | 2.059 | 0.320 |
| H | -3.009 | 1.643 | -0.316 |
| H | -2.951 | 2.070 | 1.120 |
| O | -2.337 | 5.126 | -1.280 |
| H | -2.910 | 4.829 | -0.573 |
| H | -2.193 | 4.448 | -1.947 |
| O | 3.314 | -4.874 | 1.662 |
| H | 2.713 | -4.597 | 0.948 |
| H | 3.276 | -5.832 | 1.716 |
| O | 2.704 | -2.602 | 2.806 |
| H | 3.001 | -3.520 | 2.798 |
| H | 3.374 | -2.113 | 2.311 |
| O | -0.816 | 0.450 | -2.503 |
| H | -0.351 | 0.937 | -3.192 |
| H | -1.483 | -0.070 | -2.961 |
| O | 4.657 | 9.912 | -2.553 |
| H | 5.294 | 10.621 | -2.736 |
| H | 3.779 | 10.283 | -2.709 |
| O | 4.662 | -0.586 | 1.618 |
| H | 5.257 | 0.112 | 1.909 |
| H | 5.219 | -1.054 | 0.986 |
| O | 7.355 | 6.849 | -3.180 |
| H | 6.974 | 6.921 | -2.295 |
| H | 8.300 | 6.743 | -3.061 |
| O | 7.287 | -2.353 | 2.343 |
| H | 6.686 | -2.473 | 3.080 |
| H | 6.765 | -2.459 | 1.547 |
| O | 6.038 | -1.096 | -0.624 |
| H | 5.904 | -2.013 | -0.325 |
| H | 6.910 | -1.111 | -1.030 |
| O | 4.283 | 12.805 | -5.871 |
| H | 4.088 | 11.907 | -5.584 |
| H | 3.490 | 13.302 | -5.667 |
| O | 6.279 | 8.200 | -0.728 |
| H | 5.588 | 8.759 | -1.130 |
| H | 6.920 | 8.832 | -0.395 |
| O | -0.047 | -3.327 | 3.283 |
| H | -0.002 | -4.101 | 3.846 |
| H | 0.866 | -3.119 | 3.028 |
| O | -0.672 | 7.068 | -3.590 |
| H | -0.038 | 7.720 | -3.886 |
| H | -1.136 | 7.390 | -2.817 |
| O | -0.861 | -7.137 | 2.929 |
| H | -0.152 | -7.616 | 3.370 |
| H | -1.354 | -6.792 | 3.681 |
| O | 3.187 | 10.395 | -5.209 |
| H | 3.539 | 9.697 | -5.774 |
| H | 2.366 | 10.026 | -4.877 |
| O | -1.647 | -2.365 | 0.941 |
| H | -1.641 | -2.128 | 1.872 |
| H | -1.520 | -3.330 | 0.897 |
| O | -3.155 | -2.948 | -1.468 |
| H | -2.838 | -2.991 | -0.561 |
| H | -3.342 | -2.024 | -1.637 |
| O | 2.016 | 10.747 | -2.208 |
| H | 1.621 | 11.513 | -2.635 |
| H | 1.715 | 10.789 | -1.295 |
| O | 7.089 | 11.338 | -2.596 |
| H | 7.497 | 11.909 | -1.942 |
| H | 7.535 | 11.531 | -3.426 |
| O | 4.951 | 6.250 | 1.078 |
| H | 5.342 | 6.989 | 0.582 |
| H | 4.140 | 6.609 | 1.447 |
| O | -2.335 | 2.993 | -3.118 |
| H | -1.768 | 2.486 | -2.530 |
| H | -2.201 | 2.671 | -4.010 |
| O | -0.163 | 4.883 | 0.876 |
| H | 0.491 | 4.323 | 0.444 |
| H | -0.748 | 5.119 | 0.141 |
| O | 5.503 | -3.764 | 0.375 |
| H | 4.720 | -4.091 | 0.848 |
| H | 6.002 | -4.507 | 0.033 |
| O | 8.617 | 2.664 | 1.271 |
| H | 9.287 | 2.897 | 1.920 |
| H | 7.872 | 3.219 | 1.516 |
| O | 2.268 | -3.287 | -0.430 |
| H | 2.837 | -3.080 | -1.177 |
| H | 1.415 | -3.558 | -0.781 |
| O | -1.240 | -5.317 | 0.841 |
| H | -0.959 | -5.860 | 0.102 |
| H | -1.224 | -5.908 | 1.608 |
| O | 0.187 | 0.234 | 2.743 |
| H | -0.371 | -0.001 | 3.489 |
| H | 1.078 | 0.222 | 3.105 |
| O | -0.998 | 10.292 | -2.661 |
| H | -1.562 | 9.602 | -2.304 |
| H | -0.550 | 9.886 | -3.404 |

**Table S2.** Atomic coordinates of the GAG^+^(H_2_O)_30_ clusters extracted at 90°C.

| B1-A1 | x (Å) | y (Å) | z (Å) |
| --- | --- | --- | --- |
| N | 0.003 | 0.007 | 0.000 |
| C | 1.485 | 0.010 | -0.003 |
| C | 1.932 | 1.478 | 0.000 |
| N | 3.094 | 1.719 | 0.597 |
| C | 3.625 | 3.018 | 0.998 |
| C | 4.786 | 3.407 | 0.070 |
| N | 5.019 | 4.704 | -0.115 |
| C | 6.278 | 5.190 | -0.655 |
| C | 6.885 | 6.267 | 0.221 |
| O | 8.203 | 6.359 | 0.061 |
| H | -0.357 | 0.488 | 0.835 |
| H | -0.338 | 0.495 | -0.843 |
| H | -0.328 | -0.961 | -0.060 |
| H | 1.849 | -0.538 | 0.862 |
| H | 1.834 | -0.474 | -0.910 |
| O | 1.142 | 2.334 | -0.405 |
| H | 3.598 | 0.917 | 0.961 |
| H | 2.815 | 3.737 | 0.890 |
| C | 4.093 | 2.988 | 2.449 |
| H | 4.289 | 3.987 | 2.838 |
| H | 3.279 | 2.590 | 3.058 |
| H | 4.945 | 2.322 | 2.582 |
| O | 5.508 | 2.513 | -0.394 |
| H | 4.407 | 5.382 | 0.334 |
| H | 6.967 | 4.358 | -0.752 |
| H | 6.142 | 5.624 | -1.648 |
| O | 6.230 | 6.983 | 0.934 |
| H | 8.588 | 6.869 | 0.803 |
| O | 4.413 | 0.565 | -2.223 |
| H | 5.115 | -0.086 | -2.156 |
| H | 4.752 | 1.339 | -1.746 |
| O | 2.273 | 3.953 | -2.279 |
| H | 1.922 | 3.357 | -1.607 |
| H | 3.234 | 3.927 | -2.216 |
| O | -0.891 | -3.919 | 2.886 |
| H | -1.589 | -3.402 | 2.469 |
| H | -0.504 | -4.511 | 2.241 |
| O | 9.537 | 7.688 | 2.202 |
| H | 9.548 | 8.645 | 2.348 |
| H | 10.323 | 7.318 | 2.607 |
| O | 0.555 | -0.286 | -3.412 |
| H | 0.835 | -1.182 | -3.234 |
| H | 1.211 | 0.178 | -3.937 |
| O | 11.440 | 10.394 | 3.801 |
| H | 11.992 | 10.154 | 3.053 |
| H | 10.545 | 10.073 | 3.699 |
| O | 4.627 | 3.518 | -3.468 |
| H | 5.575 | 3.364 | -3.473 |
| H | 4.418 | 3.946 | -4.301 |
| O | -4.359 | -7.048 | -0.389 |
| H | -3.593 | -6.734 | -0.874 |
| H | -4.901 | -6.281 | -0.166 |
| O | 1.590 | -2.650 | 4.055 |
| H | 0.827 | -2.990 | 3.561 |
| H | 1.320 | -2.511 | 4.964 |
| O | 10.947 | 8.596 | -0.684 |
| H | 11.114 | 7.650 | -0.703 |
| H | 11.414 | 8.901 | 0.108 |
| O | 8.942 | 10.550 | 2.729 |
| H | 9.213 | 11.078 | 1.974 |
| H | 8.337 | 11.094 | 3.238 |
| O | -1.653 | 1.270 | -1.873 |
| H | -1.739 | 2.222 | -1.980 |
| H | -1.926 | 0.883 | -2.713 |
| O | 7.811 | 6.375 | -3.355 |
| H | 7.875 | 5.690 | -4.024 |
| H | 8.621 | 6.880 | -3.472 |
| O | 4.415 | -0.522 | 1.745 |
| H | 5.221 | -0.550 | 1.218 |
| H | 4.696 | -0.603 | 2.662 |
| O | 12.110 | 11.951 | -1.054 |
| H | 11.216 | 12.019 | -1.397 |
| H | 12.349 | 11.035 | -1.216 |
| O | 8.556 | 9.659 | -1.634 |
| H | 8.666 | 10.543 | -1.278 |
| H | 9.372 | 9.205 | -1.380 |
| O | 12.762 | 9.093 | 1.638 |
| H | 12.603 | 8.319 | 2.181 |
| H | 13.629 | 9.017 | 1.231 |
| O | 0.408 | -2.640 | -0.935 |
| H | 0.448 | -3.417 | -0.368 |
| H | 0.855 | -2.931 | -1.734 |
| O | -2.787 | -1.255 | -1.240 |
| H | -2.494 | -0.333 | -1.217 |
| H | -3.747 | -1.258 | -1.168 |
| O | -5.025 | -4.313 | -1.575 |
| H | -4.993 | -3.357 | -1.600 |
| H | -4.760 | -4.644 | -2.436 |
| O | -2.064 | -1.907 | 1.161 |
| H | -2.538 | -1.801 | 0.316 |
| H | -2.437 | -1.311 | 1.814 |
| O | -0.690 | 2.051 | 2.004 |
| H | -1.634 | 2.135 | 1.859 |
| H | -0.239 | 2.753 | 1.522 |
| O | 2.264 | 6.185 | 0.588 |
| H | 1.905 | 5.440 | 0.093 |
| H | 2.393 | 6.868 | -0.076 |
| O | -5.754 | -5.295 | 1.238 |
| H | -5.279 | -4.484 | 1.028 |
| H | -5.120 | -5.879 | 1.665 |
| O | 7.969 | 2.912 | 1.192 |
| H | 7.152 | 2.596 | 0.782 |
| H | 8.077 | 2.397 | 1.993 |
| O | 6.851 | 7.098 | 3.882 |
| H | 7.416 | 7.007 | 3.109 |
| H | 7.150 | 7.893 | 4.327 |
| O | 3.515 | -3.225 | 1.825 |
| H | 3.864 | -2.385 | 1.522 |
| H | 2.832 | -3.086 | 2.490 |
| O | 4.806 | 5.446 | 4.768 |
| H | 5.013 | 4.817 | 5.461 |
| H | 5.634 | 5.908 | 4.561 |
| O | -5.385 | -2.586 | 0.841 |
| H | -6.311 | -2.530 | 1.104 |
| H | -4.977 | -1.880 | 1.350 |
| O | -1.759 | -6.423 | -1.437 |
| H | -1.782 | -5.516 | -1.746 |
| H | -0.846 | -6.715 | -1.450 |
|  |  |  |  |
| B1-A2 |  |  |  |
| N | 0.025 | -0.001 | -0.009 |
| C | 1.506 | -0.007 | -0.012 |
| C | 2.111 | 1.378 | -0.013 |
| N | 3.387 | 1.411 | 0.373 |
| C | 4.108 | 2.649 | 0.594 |
| C | 4.758 | 3.175 | -0.693 |
| N | 5.029 | 4.492 | -0.646 |
| C | 5.909 | 5.182 | -1.558 |
| C | 5.100 | 6.100 | -2.468 |
| O | 5.508 | 6.087 | -3.719 |
| H | -0.343 | -0.865 | -0.398 |
| H | -0.378 | 0.278 | 0.908 |
| H | -0.265 | 0.779 | -0.623 |
| H | 1.851 | -0.589 | 0.839 |
| H | 1.838 | -0.514 | -0.919 |
| O | 1.452 | 2.339 | -0.397 |
| H | 3.855 | 0.531 | 0.557 |
| H | 3.403 | 3.375 | 0.998 |
| C | 5.206 | 2.415 | 1.634 |
| H | 5.458 | 3.355 | 2.124 |
| H | 4.755 | 1.897 | 2.485 |
| H | 6.073 | 1.878 | 1.262 |
| O | 5.189 | 2.412 | -1.542 |
| H | 4.509 | 5.059 | 0.012 |
| H | 6.615 | 5.808 | -1.011 |
| H | 6.449 | 4.445 | -2.147 |
| O | 4.215 | 6.802 | -2.029 |
| H | 5.057 | 6.779 | -4.246 |
| O | 1.694 | 5.460 | -2.253 |
| H | 2.134 | 6.273 | -1.983 |
| H | 1.911 | 4.819 | -1.570 |
| O | -3.674 | 0.872 | -2.150 |
| H | -3.449 | 0.233 | -1.459 |
| H | -3.967 | 0.375 | -2.917 |
| O | -1.077 | 1.068 | 2.177 |
| H | -1.839 | 1.554 | 1.848 |
| H | -1.323 | 0.694 | 3.027 |
| O | 0.263 | -2.495 | 4.192 |
| H | 0.867 | -2.901 | 3.545 |
| H | 0.888 | -2.089 | 4.806 |
| O | -0.921 | -2.204 | 1.777 |
| H | -0.473 | -2.203 | 2.640 |
| H | -1.855 | -2.386 | 1.959 |
| O | -3.861 | -2.565 | 2.051 |
| H | -4.138 | -3.488 | 1.941 |
| H | -4.618 | -2.081 | 1.705 |
| O | 4.593 | -1.473 | 0.742 |
| H | 5.462 | -1.520 | 0.333 |
| H | 4.133 | -2.253 | 0.424 |
| O | -3.145 | -1.624 | -0.757 |
| H | -3.104 | -2.333 | -0.098 |
| H | -3.481 | -2.120 | -1.513 |
| O | 3.667 | 0.718 | -3.217 |
| H | 4.224 | 1.274 | -2.653 |
| H | 2.756 | 1.036 | -3.178 |
| O | 5.262 | 9.434 | -7.036 |
| H | 4.986 | 8.509 | -7.101 |
| H | 6.208 | 9.418 | -6.875 |
| O | 1.939 | -5.236 | 5.317 |
| H | 1.466 | -5.188 | 6.153 |
| H | 2.792 | -5.600 | 5.573 |
| O | -0.179 | -1.704 | -2.420 |
| H | -1.014 | -1.899 | -2.852 |
| H | 0.448 | -1.523 | -3.128 |
| O | 3.435 | -2.015 | 5.162 |
| H | 3.458 | -1.495 | 4.356 |
| H | 3.087 | -2.866 | 4.875 |
| O | 4.533 | 6.888 | -6.377 |
| H | 3.651 | 6.531 | -6.211 |
| H | 5.036 | 6.119 | -6.667 |
| O | 0.935 | 1.512 | -3.189 |
| H | 0.371 | 1.192 | -3.893 |
| H | 0.381 | 1.834 | -2.468 |
| O | 2.469 | -3.642 | 2.862 |
| H | 2.974 | -3.618 | 2.050 |
| H | 2.358 | -4.558 | 3.141 |
| O | 9.151 | 3.059 | 2.899 |
| H | 9.192 | 2.411 | 2.194 |
| H | 9.112 | 3.930 | 2.506 |
| O | 0.301 | -3.464 | -0.270 |
| H | -0.054 | -3.044 | 0.530 |
| H | -0.229 | -3.122 | -0.992 |
| O | -1.131 | 1.900 | -1.607 |
| H | -1.829 | 1.313 | -1.946 |
| H | -1.356 | 2.799 | -1.864 |
| O | 8.584 | 6.016 | -4.998 |
| H | 8.766 | 5.336 | -4.345 |
| H | 9.316 | 6.635 | -5.020 |
| O | 4.861 | 9.640 | -4.215 |
| H | 5.072 | 9.887 | -5.125 |
| H | 5.668 | 9.335 | -3.793 |
| O | 1.203 | 4.364 | 1.406 |
| H | 1.160 | 3.821 | 0.612 |
| H | 1.861 | 5.035 | 1.211 |
| O | -4.367 | -7.565 | 2.430 |
| H | -3.750 | -7.577 | 1.695 |
| H | -4.115 | -6.811 | 2.979 |
| O | 2.755 | 4.571 | -5.173 |
| H | 2.006 | 4.027 | -5.429 |
| H | 2.569 | 4.820 | -4.253 |
| O | 4.962 | 2.891 | -4.899 |
| H | 4.364 | 3.646 | -4.951 |
| H | 4.346 | 2.168 | -4.725 |
| O | -6.025 | -4.488 | 1.174 |
| H | -5.783 | -5.413 | 1.050 |
| H | -6.777 | -4.520 | 1.772 |
| O | 9.507 | 4.299 | -0.602 |
| H | 8.850 | 3.614 | -0.461 |
| H | 9.416 | 4.906 | 0.135 |
| O | 3.116 | 6.020 | 0.873 |
| H | 3.053 | 6.841 | 0.368 |
| H | 3.254 | 6.321 | 1.779 |
| O | 7.273 | 8.288 | -3.284 |
| H | 7.785 | 7.555 | -3.654 |
| H | 7.844 | 9.060 | -3.314 |
| O | -4.500 | -5.182 | 4.114 |
| H | -4.079 | -4.318 | 4.124 |
| H | -4.659 | -5.397 | 5.037 |
|  |  |  |  |
| B1-B1 |  |  |  |
| N | 0.003 | -0.002 | -0.003 |
| C | 1.492 | -0.002 | -0.005 |
| C | 2.102 | 1.402 | -0.006 |
| N | 3.095 | 1.634 | -0.854 |
| C | 3.671 | 2.956 | -1.090 |
| C | 4.344 | 2.886 | -2.456 |
| N | 4.295 | 3.975 | -3.221 |
| C | 5.015 | 4.088 | -4.462 |
| C | 5.932 | 5.294 | -4.481 |
| O | 6.770 | 5.232 | -5.493 |
| H | -0.362 | -0.874 | 0.407 |
| H | -0.361 | 0.797 | 0.543 |
| H | -0.346 | 0.032 | -0.968 |
| H | 1.819 | -0.501 | 0.908 |
| H | 1.826 | -0.599 | -0.847 |
| O | 1.567 | 2.256 | 0.705 |
| H | 3.505 | 0.889 | -1.410 |
| H | 2.875 | 3.700 | -1.077 |
| C | 4.740 | 3.303 | -0.047 |
| H | 5.532 | 2.552 | -0.096 |
| H | 5.137 | 4.306 | -0.174 |
| H | 4.341 | 3.145 | 0.956 |
| O | 4.867 | 1.829 | -2.799 |
| H | 3.994 | 4.846 | -2.795 |
| H | 5.622 | 3.198 | -4.615 |
| H | 4.331 | 4.149 | -5.314 |
| O | 6.022 | 6.063 | -3.548 |
| H | 7.464 | 5.945 | -5.438 |
| O | 7.565 | 1.478 | -2.513 |
| H | 8.243 | 1.975 | -2.973 |
| H | 6.709 | 1.820 | -2.786 |
| O | 2.573 | 6.082 | -2.201 |
| H | 2.204 | 6.949 | -2.382 |
| H | 2.228 | 5.479 | -2.882 |
| O | 8.538 | 7.201 | -5.813 |
| H | 7.925 | 7.925 | -5.962 |
| H | 9.089 | 7.412 | -5.033 |
| O | -0.324 | 3.555 | -1.552 |
| H | -0.957 | 4.255 | -1.729 |
| H | -0.240 | 3.454 | -0.601 |
| O | 10.511 | 10.204 | -10.235 |
| H | 10.061 | 9.677 | -10.898 |
| H | 9.891 | 10.890 | -9.978 |
| O | 2.831 | 4.004 | 2.756 |
| H | 2.284 | 3.336 | 2.328 |
| H | 3.063 | 4.622 | 2.061 |
| O | 1.769 | 2.033 | -5.989 |
| H | 2.188 | 2.234 | -6.835 |
| H | 0.833 | 1.996 | -6.211 |
| O | -1.287 | 2.485 | 1.110 |
| H | -2.115 | 2.431 | 1.596 |
| H | -0.924 | 3.353 | 1.308 |
| O | 9.458 | 7.920 | -3.361 |
| H | 10.328 | 8.142 | -3.021 |
| H | 8.873 | 8.686 | -3.291 |
| O | 6.661 | 6.823 | 0.525 |
| H | 6.921 | 6.011 | 0.080 |
| H | 6.842 | 7.530 | -0.095 |
| O | 6.836 | 8.833 | -3.432 |
| H | 6.695 | 7.983 | -2.996 |
| H | 5.984 | 9.055 | -3.811 |
| O | 1.373 | -3.618 | 3.279 |
| H | 1.486 | -3.043 | 4.047 |
| H | 0.923 | -3.036 | 2.660 |
| O | 10.197 | 4.985 | -6.085 |
| H | 9.602 | 5.732 | -5.991 |
| H | 9.817 | 4.442 | -6.777 |
| O | -2.889 | -1.314 | -1.565 |
| H | -2.632 | -2.231 | -1.458 |
| H | -2.363 | -0.908 | -2.260 |
| O | 1.652 | -3.020 | -1.296 |
| H | 0.951 | -3.563 | -0.920 |
| H | 2.439 | -3.561 | -1.143 |
| O | 5.702 | -0.100 | -4.700 |
| H | 5.312 | 0.481 | -4.038 |
| H | 4.950 | -0.315 | -5.257 |
| O | 4.112 | -1.814 | -2.534 |
| H | 3.980 | -1.123 | -3.201 |
| H | 3.234 | -1.916 | -2.170 |
| O | 7.237 | 6.235 | -10.267 |
| H | 8.013 | 6.754 | -10.039 |
| H | 7.559 | 5.638 | -10.948 |
| O | -0.443 | -0.275 | 4.258 |
| H | 0.295 | -0.541 | 4.829 |
| H | -1.105 | 0.112 | 4.836 |
| O | 8.868 | 5.295 | -1.545 |
| H | 8.360 | 4.651 | -2.043 |
| H | 9.010 | 6.023 | -2.163 |
| O | 4.644 | 1.659 | 4.539 |
| H | 4.704 | 0.704 | 4.604 |
| H | 3.717 | 1.872 | 4.666 |
| O | -0.821 | 0.786 | -2.585 |
| H | -0.694 | 0.851 | -3.534 |
| H | -1.268 | 1.602 | -2.321 |
| O | 1.230 | -2.069 | -3.733 |
| H | 1.410 | -2.437 | -2.857 |
| H | 0.281 | -2.045 | -3.867 |
| O | 5.123 | 3.422 | -8.510 |
| H | 5.702 | 4.069 | -8.926 |
| H | 5.726 | 2.744 | -8.193 |
| O | 1.475 | -1.907 | 5.749 |
| H | 0.732 | -2.235 | 6.265 |
| H | 2.103 | -1.583 | 6.402 |
| O | 9.338 | 10.793 | -5.561 |
| H | 8.842 | 11.024 | -4.774 |
| H | 10.266 | 10.772 | -5.315 |
| O | 1.406 | 3.957 | -3.788 |
| H | 1.601 | 3.330 | -4.496 |
| H | 0.932 | 3.503 | -3.079 |
| O | -0.952 | -1.643 | 1.837 |
| H | -0.788 | -1.203 | 2.699 |
| H | -1.277 | -2.519 | 2.066 |
| O | 2.898 | -0.003 | -4.287 |
| H | 2.731 | 0.731 | -4.890 |
| H | 2.078 | -0.518 | -4.313 |
| O | 12.923 | 9.414 | -8.812 |
| H | 13.570 | 9.130 | -9.459 |
| H | 12.172 | 9.754 | -9.315 |
|  |  |  |  |
| B1-B2 |  |  |  |
| N | 0.015 | 0.011 | 0.003 |
| C | 1.487 | 0.012 | 0.004 |
| C | 2.031 | 1.437 | 0.006 |
| N | 3.378 | 1.525 | 0.001 |
| C | 4.052 | 2.782 | -0.276 |
| C | 3.795 | 3.269 | -1.703 |
| N | 3.305 | 4.515 | -1.771 |
| C | 2.982 | 5.165 | -3.008 |
| C | 1.588 | 5.737 | -2.921 |
| O | 1.044 | 5.953 | -4.106 |
| H | -0.350 | 0.774 | 0.598 |
| H | -0.369 | 0.128 | -0.945 |
| H | -0.354 | -0.823 | 0.486 |
| H | 1.836 | -0.509 | 0.894 |
| H | 1.850 | -0.530 | -0.867 |
| O | 1.279 | 2.394 | 0.039 |
| H | 3.901 | 0.679 | -0.195 |
| H | 3.588 | 3.498 | 0.406 |
| C | 5.544 | 2.671 | -0.007 |
| H | 5.994 | 1.880 | -0.607 |
| H | 6.029 | 3.598 | -0.311 |
| H | 5.740 | 2.519 | 1.054 |
| O | 3.978 | 2.547 | -2.673 |
| H | 3.036 | 4.996 | -0.927 |
| H | 3.662 | 5.997 | -3.216 |
| H | 3.062 | 4.440 | -3.815 |
| O | 1.097 | 6.100 | -1.874 |
| H | 0.079 | 6.146 | -3.952 |
| O | -3.860 | -2.983 | 0.233 |
| H | -4.553 | -2.685 | 0.823 |
| H | -3.009 | -2.931 | 0.680 |
| O | -1.509 | 6.262 | -3.715 |
| H | -1.998 | 6.251 | -2.894 |
| H | -1.991 | 6.754 | -4.395 |
| O | 0.728 | -3.187 | -0.688 |
| H | 1.572 | -3.632 | -0.568 |
| H | 0.393 | -3.553 | -1.513 |
| O | 5.222 | -3.268 | 4.852 |
| H | 5.596 | -3.006 | 4.006 |
| H | 4.648 | -2.529 | 5.108 |
| O | 4.932 | 10.491 | -4.562 |
| H | 4.261 | 9.797 | -4.483 |
| H | 4.860 | 11.007 | -3.756 |
| O | 5.122 | 0.070 | 2.792 |
| H | 5.449 | 0.965 | 2.916 |
| H | 5.900 | -0.503 | 2.855 |
| O | 7.519 | 9.323 | -4.272 |
| H | 6.647 | 9.671 | -4.523 |
| H | 8.120 | 10.035 | -4.500 |
| O | 4.865 | 8.295 | -0.473 |
| H | 4.754 | 9.207 | -0.749 |
| H | 5.815 | 8.157 | -0.507 |
| O | -1.750 | 1.886 | 0.928 |
| H | -1.592 | 2.814 | 0.717 |
| H | -2.655 | 1.717 | 0.659 |
| O | 2.911 | 8.728 | -4.532 |
| H | 1.950 | 8.678 | -4.509 |
| H | 3.137 | 8.130 | -5.251 |
| O | -1.103 | -2.199 | 1.127 |
| H | -0.662 | -2.846 | 0.552 |
| H | -1.103 | -2.628 | 1.992 |
| O | 6.942 | 2.471 | -3.042 |
| H | 5.981 | 2.489 | -3.062 |
| H | 7.291 | 3.289 | -2.681 |
| O | -1.993 | -4.597 | 3.783 |
| H | -2.159 | -5.447 | 3.373 |
| H | -1.053 | -4.418 | 3.678 |
| O | -3.224 | -1.226 | -2.337 |
| H | -3.044 | -1.891 | -1.664 |
| H | -2.433 | -0.678 | -2.383 |
| O | -2.955 | 1.885 | -4.128 |
| H | -3.824 | 2.130 | -3.783 |
| H | -3.108 | 1.056 | -4.592 |
| O | -2.605 | 8.313 | -5.716 |
| H | -2.947 | 8.704 | -6.524 |
| H | -1.684 | 8.137 | -5.982 |
| O | -0.091 | 8.144 | -6.459 |
| H | 0.387 | 7.576 | -7.069 |
| H | 0.117 | 9.054 | -6.744 |
| O | 0.495 | -4.029 | 2.717 |
| H | 1.029 | -3.395 | 3.204 |
| H | 0.857 | -4.910 | 2.857 |
| O | 4.812 | -1.107 | 0.123 |
| H | 5.025 | -1.023 | 1.060 |
| H | 5.285 | -1.876 | -0.198 |
| O | 6.852 | -1.943 | 2.245 |
| H | 7.799 | -2.020 | 2.401 |
| H | 6.674 | -2.611 | 1.578 |
| O | 3.671 | -5.861 | 4.901 |
| H | 4.063 | -6.654 | 5.270 |
| H | 4.225 | -5.118 | 5.176 |
| O | 7.930 | 4.767 | -2.425 |
| H | 8.258 | 4.772 | -1.524 |
| H | 8.454 | 5.434 | -2.877 |
| O | -5.002 | 1.964 | -2.272 |
| H | -5.606 | 1.258 | -2.515 |
| H | -4.942 | 1.951 | -1.316 |
| O | -1.188 | 5.756 | -0.048 |
| H | -1.879 | 6.078 | -0.629 |
| H | -0.392 | 5.824 | -0.599 |
| O | 0.242 | 0.314 | 3.055 |
| H | 0.840 | 0.877 | 3.555 |
| H | 0.075 | -0.415 | 3.657 |
| O | -0.843 | -0.004 | -2.530 |
| H | -0.605 | 0.767 | -3.090 |
| H | -0.546 | -0.766 | -3.037 |
| O | 3.523 | -0.870 | 4.857 |
| H | 3.605 | -0.072 | 5.381 |
| H | 4.033 | -0.765 | 4.043 |
| O | 10.015 | 6.579 | -1.003 |
| H | 10.351 | 5.949 | -0.361 |
| H | 10.801 | 6.916 | -1.440 |
| O | 0.570 | 10.912 | -7.050 |
| H | -0.098 | 11.209 | -6.424 |
| H | 0.380 | 11.386 | -7.864 |
| O | -0.278 | 2.077 | -4.157 |
| H | -1.253 | 2.077 | -4.226 |
| H | 0.069 | 2.569 | -4.901 |

**Table S3.** Atomic coordinates of the (GVGVP)_2_(H_2_O)_40_ clusters extracted at 15°C.

| B1-A1 | x (Å) | y (Å) | z (Å) |
| --- | --- | --- | --- |
| N | 0.006 | -0.005 | 0.006 |
| C | 1.468 | -0.006 | 0.005 |
| C | 2.029 | 1.417 | 0.005 |
| H | 1.741 | -0.426 | 0.973 |
| H | 1.834 | -0.498 | -0.902 |
| N | 3.324 | 1.600 | 0.262 |
| O | 1.334 | 2.380 | -0.313 |
| C | 4.018 | 2.872 | 0.162 |
| H | 3.822 | 0.778 | 0.540 |
| C | 4.349 | 3.127 | -1.315 |
| C | 5.278 | 2.795 | 1.023 |
| H | 3.341 | 3.680 | 0.429 |
| C | 5.095 | 2.362 | 2.483 |
| C | 6.006 | 4.139 | 1.045 |
| H | 5.908 | 2.089 | 0.504 |
| N | 4.992 | 2.172 | -1.986 |
| O | 3.949 | 4.146 | -1.862 |
| C | 5.115 | 2.152 | -3.431 |
| H | 5.056 | 1.303 | -1.482 |
| C | 4.428 | 0.896 | -3.957 |
| H | 4.557 | 3.036 | -3.751 |
| H | 6.160 | 2.162 | -3.731 |
| N | 5.068 | 0.129 | -4.840 |
| O | 3.334 | 0.528 | -3.513 |
| C | 4.675 | -1.251 | -5.049 |
| H | 5.983 | 0.420 | -5.156 |
| C | 5.607 | -2.170 | -4.274 |
| N | 5.136 | -3.316 | -3.769 |
| C | 5.920 | -4.217 | -2.955 |
| C | 3.737 | -3.679 | -3.617 |
| C | 4.966 | -4.946 | -1.994 |
| C | 6.672 | -5.327 | -3.683 |
| C | 3.717 | -5.016 | -2.885 |
| N | 7.583 | -5.958 | -2.923 |
| C | 8.438 | -6.998 | -3.446 |
| C | 9.915 | -6.639 | -3.395 |
| N | 10.801 | -7.625 | -3.588 |
| C | 10.592 | -9.016 | -3.917 |
| C | 10.325 | -9.151 | -5.416 |
| N | 11.124 | -8.453 | -6.222 |
| C | 11.082 | -8.389 | -7.670 |
| C | 10.866 | -6.964 | -8.187 |
| N | 10.918 | -5.977 | -7.291 |
| C | 10.794 | -4.565 | -7.616 |
| C | 11.983 | -3.793 | -7.042 |
| N | 12.485 | -2.758 | -7.726 |
| C | 13.662 | -2.070 | -7.256 |
| C | 12.094 | -2.367 | -9.066 |
| C | 14.123 | -1.287 | -8.479 |
| C | 12.908 | -1.118 | -9.393 |
| C | 9.853 | -10.027 | -3.034 |
| C | 9.399 | -4.021 | -7.269 |
| C | 13.340 | -1.080 | -6.130 |
| C | 4.758 | -1.495 | -6.566 |
| H | 6.600 | -3.619 | -2.349 |
| H | 5.386 | -5.919 | -1.754 |
| H | 4.727 | -4.357 | -1.114 |
| H | 3.802 | -5.891 | -3.527 |
| H | 2.812 | -5.117 | -2.286 |
| H | 3.224 | -3.713 | -4.578 |
| H | 3.307 | -2.892 | -2.998 |
| O | 6.417 | -5.737 | -4.811 |
| H | 7.669 | -5.753 | -1.939 |
| H | 8.156 | -7.906 | -2.899 |
| H | 8.155 | -7.239 | -4.466 |
| O | 10.249 | -5.508 | -3.055 |
| H | 11.754 | -7.309 | -3.570 |
| H | 11.578 | -9.441 | -3.720 |
| O | 9.320 | -9.728 | -5.823 |
| H | 11.902 | -7.919 | -5.859 |
| H | 12.028 | -8.763 | -8.052 |
| H | 10.201 | -8.949 | -7.966 |
| O | 10.633 | -6.794 | -9.384 |
| H | 11.180 | -6.200 | -6.344 |
| H | 10.906 | -4.517 | -8.702 |
| O | 12.460 | -4.158 | -5.970 |
| H | 14.499 | -2.639 | -6.858 |
| H | 14.544 | -0.296 | -8.308 |
| H | 14.898 | -1.876 | -8.970 |
| H | 12.390 | -0.201 | -9.132 |
| H | 13.219 | -1.060 | -10.435 |
| H | 11.036 | -2.126 | -9.196 |
| H | 12.301 | -3.150 | -9.786 |
| H | 3.620 | -1.365 | -4.824 |
| O | 6.744 | -1.747 | -4.097 |
| C | 3.836 | -0.524 | -7.302 |
| C | 4.371 | -2.922 | -6.957 |
| H | 5.739 | -1.167 | -6.917 |
| C | 10.192 | -9.762 | -1.559 |
| C | 10.067 | -11.499 | -3.367 |
| H | 8.780 | -9.858 | -3.125 |
| C | 9.053 | -3.855 | -5.792 |
| C | 8.293 | -4.767 | -8.020 |
| H | 9.414 | -3.012 | -7.690 |
| O | 14.238 | -0.297 | -5.768 |
| O | 12.189 | -1.003 | -5.620 |
| H | -0.340 | 0.484 | -0.820 |
| H | -0.357 | -0.941 | 0.106 |
| H | -0.325 | 0.570 | 0.779 |
| H | 6.092 | 2.443 | 2.904 |
| H | 4.455 | 3.036 | 3.046 |
| H | 4.763 | 1.334 | 2.626 |
| H | 5.342 | 4.901 | 1.460 |
| H | 6.812 | 4.155 | 1.783 |
| H | 6.391 | 4.444 | 0.077 |
| H | 3.816 | -0.725 | -8.382 |
| H | 2.802 | -0.656 | -6.960 |
| H | 4.016 | 0.536 | -7.122 |
| H | 3.333 | -3.076 | -6.659 |
| H | 4.365 | -3.103 | -8.029 |
| H | 5.075 | -3.626 | -6.525 |
| H | 11.255 | -9.941 | -1.415 |
| H | 9.932 | -8.744 | -1.277 |
| H | 9.494 | -10.374 | -0.993 |
| H | 9.606 | -12.290 | -2.765 |
| H | 9.950 | -11.716 | -4.428 |
| H | 11.111 | -11.737 | -3.175 |
| H | 8.090 | -3.372 | -5.650 |
| H | 8.925 | -4.827 | -5.308 |
| H | 9.877 | -3.406 | -5.224 |
| H | 8.439 | -4.806 | -9.097 |
| H | 8.225 | -5.807 | -7.706 |
| H | 7.346 | -4.311 | -7.731 |
| O | 6.216 | 3.340 | -6.773 |
| H | 6.618 | 4.057 | -6.262 |
| H | 6.605 | 3.322 | -7.690 |
| O | 8.918 | 0.194 | -9.570 |
| H | 9.226 | -0.086 | -8.685 |
| H | 7.986 | -0.122 | -9.706 |
| O | 7.715 | -5.600 | -0.054 |
| H | 8.243 | -6.259 | 0.438 |
| H | 6.768 | -5.683 | 0.205 |
| O | 5.250 | -0.368 | -0.851 |
| H | 6.086 | -0.816 | -1.146 |
| H | 4.881 | -0.857 | -0.079 |
| O | 2.798 | 6.151 | -3.523 |
| H | 3.109 | 5.513 | -2.840 |
| H | 3.274 | 6.007 | -4.369 |
| O | 10.011 | -0.217 | -6.850 |
| H | 9.260 | -0.077 | -6.206 |
| H | 10.829 | -0.479 | -6.340 |
| O | 2.835 | -6.916 | 0.346 |
| H | 3.764 | -6.573 | 0.477 |
| H | 2.225 | -6.151 | 0.197 |
| O | 8.902 | 2.443 | -3.414 |
| H | 8.832 | 2.524 | -2.425 |
| H | 8.898 | 3.329 | -3.821 |
| O | 13.009 | -6.211 | -4.158 |
| H | 13.534 | -6.470 | -4.951 |
| H | 12.889 | -5.214 | -4.143 |
| O | 4.803 | -9.329 | -2.480 |
| H | 3.861 | -9.088 | -2.650 |
| H | 5.377 | -8.978 | -3.211 |
| O | 11.686 | -1.718 | -3.197 |
| H | 11.980 | -1.395 | -4.089 |
| H | 10.730 | -1.509 | -3.071 |
| O | 8.789 | -11.900 | -7.216 |
| H | 9.212 | -11.346 | -6.512 |
| H | 9.485 | -12.288 | -7.790 |
| O | 7.444 | -8.983 | -7.612 |
| H | 7.681 | -9.468 | -8.439 |
| H | 8.080 | -9.229 | -6.890 |
| O | 13.214 | -12.317 | -6.898 |
| H | 13.716 | -12.966 | -7.464 |
| H | 12.967 | -12.746 | -6.042 |
| O | 7.828 | 1.187 | -5.698 |
| H | 8.137 | 1.694 | -4.897 |
| H | 7.397 | 1.785 | -6.295 |
| O | 0.642 | 0.530 | -4.322 |
| H | 1.558 | 0.576 | -3.972 |
| H | 0.585 | 1.019 | -5.188 |
| O | 9.071 | -3.572 | -1.397 |
| H | 8.825 | -4.352 | -0.889 |
| H | 9.543 | -3.839 | -2.213 |
| O | 12.816 | -8.787 | -11.690 |
| H | 12.344 | -8.173 | -12.274 |
| H | 13.684 | -8.398 | -11.419 |
| O | 15.013 | -6.783 | -10.080 |
| H | 14.977 | -6.780 | -9.085 |
| H | 14.648 | -5.943 | -10.422 |
| O | 3.771 | -1.726 | 1.136 |
| H | 3.266 | -2.506 | 1.474 |
| H | 4.557 | -1.573 | 1.700 |
| O | 12.651 | -4.106 | -2.074 |
| H | 12.364 | -3.311 | -2.625 |
| H | 11.988 | -4.820 | -2.177 |
| O | 5.230 | -6.121 | 1.034 |
| H | 5.199 | -5.425 | 1.732 |
| H | 5.521 | -6.980 | 1.416 |
| O | 0.758 | -2.104 | -4.960 |
| H | 0.589 | -1.164 | -4.722 |
| H | 1.080 | -2.599 | -4.159 |
| O | 8.067 | -0.928 | -0.769 |
| H | 8.477 | -0.778 | -1.657 |
| H | 8.335 | -1.801 | -0.432 |
| O | 2.241 | -3.580 | 2.654 |
| H | 2.234 | -4.221 | 3.388 |
| H | 1.742 | -2.781 | 2.921 |
| O | 10.207 | -4.556 | -11.428 |
| H | 11.100 | -4.166 | -11.601 |
| H | 10.328 | -5.368 | -10.896 |
| O | 9.108 | -0.324 | -3.161 |
| H | 8.360 | -0.667 | -3.689 |
| H | 9.300 | 0.587 | -3.435 |
| O | 1.052 | 5.199 | 0.667 |
| H | 1.135 | 4.641 | -0.145 |
| H | 0.702 | 4.640 | 1.416 |
| O | 5.544 | -7.082 | -7.062 |
| H | 5.755 | -6.661 | -6.190 |
| H | 6.208 | -7.797 | -7.252 |
| O | 10.984 | -12.649 | -8.542 |
| H | 11.263 | -12.228 | -9.387 |
| H | 11.719 | -12.549 | -7.869 |
| O | 9.230 | 2.302 | -0.197 |
| H | 8.671 | 1.500 | -0.146 |
| H | 10.110 | 2.066 | -0.533 |
| O | 14.015 | -9.456 | -5.718 |
| H | 14.674 | -9.366 | -4.982 |
| H | 13.779 | -10.413 | -5.814 |
| O | -0.593 | 3.975 | 2.241 |
| H | -1.157 | 4.547 | 1.691 |
| H | -0.726 | 3.024 | 2.000 |
| O | 0.369 | 1.211 | 2.924 |
| H | 0.603 | 0.268 | 3.158 |
| H | 0.896 | 1.827 | 3.485 |
| O | 8.503 | 4.540 | -5.349 |
| H | 7.971 | 4.712 | -4.550 |
| H | 8.593 | 5.350 | -5.854 |
| O | 14.684 | -6.127 | -6.570 |
| H | 15.286 | -6.911 | -6.542 |
| H | 15.180 | -5.324 | -6.283 |
| O | 11.781 | 1.428 | -3.991 |
| H | 11.998 | 2.225 | -4.478 |
| H | 12.055 | 0.637 | -4.524 |
| O | 11.109 | 2.142 | -8.109 |
| H | 12.069 | 2.086 | -7.902 |
| H | 10.614 | 1.459 | -7.603 |
| O | 7.316 | 5.414 | -2.987 |
| H | 7.237 | 5.076 | -2.065 |
| H | 7.262 | 6.407 | -2.983 |
| O | 8.679 | -2.475 | -10.907 |
| H | 9.143 | -3.292 | -11.177 |
| H | 9.305 | -1.882 | -10.420 |
|  |  |  |  |
| B1-A2 |  |  |  |
| N | 0.000 | 0.000 | -0.006 |
| C | 1.479 | -0.002 | -0.005 |
| C | 2.036 | 1.422 | -0.002 |
| H | 1.760 | -0.444 | 0.954 |
| H | 1.844 | -0.493 | -0.908 |
| N | 3.330 | 1.580 | 0.261 |
| O | 1.344 | 2.389 | -0.304 |
| C | 4.031 | 2.857 | 0.164 |
| H | 3.840 | 0.752 | 0.533 |
| C | 4.365 | 3.120 | -1.313 |
| C | 5.299 | 2.781 | 1.022 |
| H | 3.354 | 3.663 | 0.443 |
| C | 5.104 | 2.344 | 2.479 |
| C | 6.012 | 4.132 | 1.032 |
| H | 5.938 | 2.072 | 0.505 |
| N | 5.008 | 2.151 | -1.968 |
| O | 3.967 | 4.145 | -1.873 |
| C | 5.138 | 2.122 | -3.410 |
| H | 5.068 | 1.281 | -1.460 |
| C | 4.445 | 0.865 | -3.933 |
| H | 4.587 | 3.003 | -3.744 |
| H | 6.183 | 2.126 | -3.709 |
| N | 5.090 | 0.090 | -4.810 |
| O | 3.348 | 0.519 | -3.480 |
| C | 4.692 | -1.292 | -5.015 |
| H | 6.015 | 0.373 | -5.119 |
| C | 5.611 | -2.221 | -4.231 |
| N | 5.103 | -3.365 | -3.734 |
| C | 5.893 | -4.256 | -2.915 |
| C | 3.695 | -3.698 | -3.603 |
| C | 4.903 | -4.961 | -1.957 |
| C | 6.641 | -5.361 | -3.652 |
| C | 3.657 | -5.027 | -2.864 |
| N | 7.558 | -5.988 | -2.901 |
| C | 8.411 | -7.024 | -3.436 |
| C | 9.885 | -6.633 | -3.386 |
| N | 10.801 | -7.600 | -3.586 |
| C | 10.587 | -8.992 | -3.923 |
| C | 10.305 | -9.148 | -5.422 |
| N | 11.113 | -8.446 | -6.228 |
| C | 11.080 | -8.381 | -7.670 |
| C | 10.867 | -6.959 | -8.195 |
| N | 10.912 | -5.969 | -7.297 |
| C | 10.789 | -4.564 | -7.643 |
| C | 11.983 | -3.795 | -7.061 |
| N | 12.488 | -2.772 | -7.764 |
| C | 13.671 | -2.080 | -7.297 |
| C | 12.098 | -2.397 | -9.111 |
| C | 14.142 | -1.311 | -8.530 |
| C | 12.922 | -1.155 | -9.442 |
| C | 9.840 | -9.971 | -3.007 |
| C | 9.410 | -3.989 | -7.304 |
| C | 13.357 | -1.077 | -6.174 |
| C | 4.790 | -1.549 | -6.537 |
| H | 6.572 | -3.667 | -2.306 |
| H | 5.298 | -5.938 | -1.700 |
| H | 4.670 | -4.360 | -1.083 |
| H | 3.738 | -5.908 | -3.499 |
| H | 2.743 | -5.110 | -2.275 |
| H | 3.193 | -3.730 | -4.569 |
| H | 3.259 | -2.908 | -2.991 |
| O | 6.386 | -5.759 | -4.784 |
| H | 7.639 | -5.789 | -1.913 |
| H | 8.164 | -7.938 | -2.892 |
| H | 8.127 | -7.254 | -4.456 |
| O | 10.208 | -5.501 | -3.049 |
| H | 11.744 | -7.262 | -3.581 |
| H | 11.585 | -9.403 | -3.742 |
| O | 9.309 | -9.738 | -5.826 |
| H | 11.892 | -7.914 | -5.856 |
| H | 12.025 | -8.755 | -8.057 |
| H | 10.204 | -8.945 | -7.984 |
| O | 10.641 | -6.788 | -9.393 |
| H | 11.173 | -6.182 | -6.348 |
| H | 10.906 | -4.534 | -8.725 |
| O | 12.457 | -4.148 | -5.984 |
| H | 14.499 | -2.661 | -6.894 |
| H | 14.571 | -0.327 | -8.352 |
| H | 14.913 | -1.905 | -9.025 |
| H | 12.399 | -0.236 | -9.186 |
| H | 13.228 | -1.105 | -10.485 |
| H | 11.040 | -2.162 | -9.233 |
| H | 12.307 | -3.193 | -9.826 |
| H | 3.636 | -1.393 | -4.789 |
| O | 6.761 | -1.841 | -4.036 |
| C | 3.875 | -0.574 | -7.284 |
| C | 4.399 | -2.976 | -6.920 |
| H | 5.779 | -1.244 | -6.882 |
| C | 10.209 | -9.671 | -1.542 |
| C | 10.083 | -11.443 | -3.324 |
| H | 8.772 | -9.811 | -3.087 |
| C | 9.087 | -3.791 | -5.829 |
| C | 8.294 | -4.730 | -8.038 |
| H | 9.442 | -2.986 | -7.741 |
| O | 14.268 | -0.313 | -5.841 |
| O | 12.186 | -1.007 | -5.668 |
| H | -0.349 | 0.494 | -0.837 |
| H | -0.363 | -0.941 | 0.087 |
| H | -0.327 | 0.564 | 0.778 |
| H | 6.093 | 2.416 | 2.924 |
| H | 4.453 | 3.014 | 3.035 |
| H | 4.771 | 1.313 | 2.609 |
| H | 5.349 | 4.893 | 1.447 |
| H | 6.826 | 4.144 | 1.764 |
| H | 6.396 | 4.432 | 0.061 |
| H | 3.864 | -0.782 | -8.358 |
| H | 2.839 | -0.693 | -6.947 |
| H | 4.078 | 0.482 | -7.106 |
| H | 3.357 | -3.133 | -6.634 |
| H | 4.410 | -3.146 | -7.996 |
| H | 5.086 | -3.694 | -6.480 |
| H | 11.264 | -9.856 | -1.382 |
| H | 9.941 | -8.652 | -1.275 |
| H | 9.523 | -10.276 | -0.938 |
| H | 9.615 | -12.202 | -2.693 |
| H | 9.944 | -11.681 | -4.379 |
| H | 11.135 | -11.669 | -3.141 |
| H | 8.133 | -3.287 | -5.689 |
| H | 8.948 | -4.756 | -5.333 |
| H | 9.920 | -3.340 | -5.281 |
| H | 8.445 | -4.788 | -9.114 |
| H | 8.215 | -5.764 | -7.701 |
| H | 7.347 | -4.259 | -7.768 |
| O | 7.754 | -7.773 | 0.441 |
| H | 8.215 | -7.000 | 0.057 |
| H | 7.959 | -7.837 | 1.379 |
| O | 9.975 | -0.459 | -7.315 |
| H | 10.666 | -0.799 | -6.704 |
| H | 10.319 | 0.310 | -7.785 |
| O | 6.721 | -9.622 | -4.747 |
| H | 7.594 | -9.719 | -5.165 |
| H | 6.188 | -8.983 | -5.244 |
| O | 4.959 | -7.674 | -6.119 |
| H | 5.188 | -6.842 | -5.665 |
| H | 4.169 | -7.533 | -6.655 |
| O | 7.636 | 0.343 | -1.058 |
| H | 7.068 | -0.026 | -1.743 |
| H | 8.261 | 1.008 | -1.461 |
| O | 4.966 | -0.902 | -0.444 |
| H | 4.431 | -1.638 | -0.116 |
| H | 5.875 | -0.963 | -0.088 |
| O | 9.535 | -2.399 | 0.127 |
| H | 8.781 | -1.980 | 0.584 |
| H | 9.504 | -2.169 | -0.818 |
| O | 7.738 | 1.095 | -5.622 |
| H | 7.480 | 1.320 | -6.539 |
| H | 8.519 | 0.521 | -5.651 |
| O | 12.192 | -6.431 | -11.507 |
| H | 11.710 | -5.613 | -11.660 |
| H | 11.911 | -6.834 | -10.653 |
| O | 13.321 | -7.112 | -5.235 |
| H | 13.691 | -7.969 | -4.941 |
| H | 13.766 | -6.831 | -6.057 |
| O | 7.598 | -1.069 | -8.746 |
| H | 8.217 | -0.962 | -8.002 |
| H | 8.015 | -1.596 | -9.436 |
| O | 1.244 | -1.295 | -3.447 |
| H | 0.848 | -1.297 | -4.323 |
| H | 1.806 | -0.497 | -3.345 |
| O | 9.566 | 2.103 | -1.917 |
| H | 9.419 | 2.818 | -2.543 |
| H | 10.034 | 1.367 | -2.366 |
| O | 12.229 | -2.711 | -3.366 |
| H | 12.198 | -3.496 | -3.921 |
| H | 12.449 | -1.934 | -3.915 |
| O | 0.291 | 4.026 | -2.037 |
| H | 0.195 | 4.946 | -1.786 |
| H | 0.777 | 3.555 | -1.346 |
| O | 10.917 | -0.252 | -2.875 |
| H | 10.457 | -1.106 | -2.799 |
| H | 11.277 | -0.150 | -3.760 |
| O | 11.768 | 1.639 | -5.956 |
| H | 12.180 | 2.279 | -5.379 |
| H | 12.069 | 0.745 | -5.719 |
| O | 8.683 | 3.466 | -4.336 |
| H | 9.590 | 3.578 | -4.667 |
| H | 8.257 | 2.709 | -4.783 |
| O | 15.096 | -5.603 | -9.809 |
| H | 15.779 | -4.937 | -9.682 |
| H | 15.004 | -5.793 | -10.751 |
| O | 3.686 | 6.456 | -0.348 |
| H | 3.764 | 5.842 | -1.124 |
| H | 4.521 | 6.910 | -0.201 |
| O | 5.648 | 5.569 | -4.558 |
| H | 6.584 | 5.791 | -4.434 |
| H | 5.267 | 5.264 | -3.713 |
| O | 14.248 | -10.680 | -7.789 |
| H | 14.245 | -11.638 | -7.689 |
| H | 13.735 | -10.428 | -8.574 |
| O | 3.877 | 3.650 | -6.368 |
| H | 4.421 | 4.112 | -5.724 |
| H | 4.034 | 4.026 | -7.244 |
| O | 0.885 | -3.280 | -1.426 |
| H | 0.904 | -2.537 | -2.064 |
| H | 0.690 | -4.094 | -1.908 |
| O | 13.916 | -10.083 | -5.127 |
| H | 13.410 | -10.746 | -4.656 |
| H | 13.987 | -10.328 | -6.068 |
| O | 5.141 | -7.619 | -0.177 |
| H | 5.013 | -8.154 | -0.977 |
| H | 6.087 | -7.573 | 0.048 |
| O | -0.680 | 1.212 | -2.240 |
| H | -1.029 | 1.140 | -3.145 |
| H | -0.325 | 2.121 | -2.099 |
| O | 14.466 | -4.522 | -4.218 |
| H | 13.989 | -4.642 | -5.060 |
| H | 15.260 | -5.074 | -4.232 |
| O | 4.713 | -9.100 | -2.455 |
| H | 5.133 | -9.363 | -3.284 |
| H | 3.988 | -8.492 | -2.637 |
| O | 9.043 | -5.447 | -0.481 |
| H | 9.660 | -5.310 | 0.247 |
| H | 9.545 | -5.503 | -1.318 |
| O | 7.504 | -0.681 | 1.387 |
| H | 8.136 | -0.249 | 1.964 |
| H | 7.667 | -0.404 | 0.454 |
| O | 9.147 | -2.515 | -2.638 |
| H | 8.252 | -2.343 | -2.981 |
| H | 9.484 | -3.340 | -3.015 |
| O | 7.064 | 1.618 | -8.299 |
| H | 6.272 | 1.908 | -8.759 |
| H | 7.392 | 0.805 | -8.708 |
| O | 9.019 | -2.650 | -10.585 |
| H | 9.608 | -2.953 | -11.285 |
| H | 8.130 | -2.980 | -10.749 |
| O | 13.194 | -9.889 | -10.057 |
| H | 12.276 | -10.164 | -10.148 |
| H | 13.430 | -9.310 | -10.796 |
| O | 14.740 | -5.678 | -7.177 |
| H | 15.527 | -5.231 | -6.849 |
| H | 14.707 | -5.586 | -8.148 |
| O | 3.207 | -2.828 | 0.252 |
| H | 2.358 | -3.014 | -0.178 |
| H | 3.357 | -3.476 | 0.951 |
| O | 1.474 | 5.647 | 0.792 |
| H | 2.355 | 5.987 | 0.537 |
| H | 1.412 | 5.606 | 1.752 |
| O | 7.394 | -10.120 | -7.863 |
| H | 7.763 | -10.989 | -8.036 |
| H | 8.105 | -9.502 | -7.687 |
| O | 16.647 | -8.089 | -8.768 |
| H | 16.162 | -8.750 | -8.252 |
| H | 16.050 | -7.373 | -9.062 |
|  |  |  |  |
| B1-B1 |  |  |  |
| N | -0.019 | 0.022 | -0.031 |
| C | 1.486 | 0.014 | -0.039 |
| C | 1.977 | 1.453 | -0.027 |
| H | 1.827 | -0.573 | 0.808 |
| H | 1.821 | -0.465 | -0.959 |
| N | 3.253 | 1.632 | 0.267 |
| O | 1.153 | 2.297 | -0.370 |
| C | 3.979 | 2.885 | 0.101 |
| H | 3.842 | 0.788 | 0.339 |
| C | 5.041 | 2.721 | -1.002 |
| C | 4.517 | 3.424 | 1.442 |
| H | 3.261 | 3.619 | -0.274 |
| C | 5.454 | 2.401 | 2.088 |
| C | 3.359 | 3.604 | 2.432 |
| H | 5.031 | 4.367 | 1.263 |
| N | 5.675 | 3.849 | -1.369 |
| O | 5.282 | 1.622 | -1.479 |
| C | 6.857 | 3.832 | -2.199 |
| H | 5.384 | 4.750 | -1.020 |
| C | 8.011 | 4.590 | -1.559 |
| H | 7.137 | 2.801 | -2.386 |
| H | 6.659 | 4.310 | -3.163 |
| N | 9.223 | 4.080 | -1.764 |
| O | 7.808 | 5.592 | -0.857 |
| C | 10.434 | 4.817 | -1.409 |
| H | 9.330 | 3.278 | -2.386 |
| C | 10.483 | 6.085 | -2.277 |
| N | 10.658 | 7.280 | -1.691 |
| C | 10.933 | 8.461 | -2.494 |
| C | 10.577 | 7.554 | -0.254 |
| C | 10.664 | 9.632 | -1.529 |
| C | 12.409 | 8.496 | -2.926 |
| C | 10.830 | 9.058 | -0.122 |
| N | 12.641 | 9.026 | -4.146 |
| C | 13.856 | 8.807 | -4.892 |
| C | 13.604 | 7.867 | -6.063 |
| N | 14.528 | 7.740 | -7.052 |
| C | 15.905 | 8.208 | -7.182 |
| C | 16.842 | 7.636 | -6.096 |
| N | 16.926 | 6.304 | -6.035 |
| C | 17.588 | 5.592 | -4.960 |
| C | 16.704 | 4.583 | -4.237 |
| N | 15.884 | 3.855 | -5.007 |
| C | 15.165 | 2.694 | -4.514 |
| C | 16.189 | 1.561 | -4.355 |
| N | 16.396 | 1.024 | -3.161 |
| C | 17.386 | -0.035 | -2.950 |
| C | 15.665 | 1.333 | -1.928 |
| C | 17.638 | 0.039 | -1.449 |
| C | 16.246 | 0.356 | -0.901 |
| C | 16.072 | 9.729 | -7.369 |
| C | 14.087 | 2.302 | -5.530 |
| C | 16.846 | -1.436 | -3.395 |
| C | 11.683 | 3.946 | -1.614 |
| H | 10.280 | 8.488 | -3.364 |
| H | 11.328 | 10.475 | -1.713 |
| H | 9.639 | 9.975 | -1.679 |
| H | 11.848 | 9.223 | 0.228 |
| H | 10.152 | 9.520 | 0.593 |
| H | 11.328 | 6.989 | 0.295 |
| H | 9.585 | 7.273 | 0.103 |
| O | 13.274 | 8.063 | -2.189 |
| H | 11.820 | 9.230 | -4.709 |
| H | 14.266 | 9.753 | -5.245 |
| H | 14.603 | 8.376 | -4.221 |
| O | 12.550 | 7.260 | -6.142 |
| H | 14.196 | 7.099 | -7.754 |
| H | 16.244 | 7.756 | -8.121 |
| O | 17.395 | 8.382 | -5.301 |
| H | 16.573 | 5.758 | -6.811 |
| H | 18.461 | 5.049 | -5.333 |
| H | 17.927 | 6.315 | -4.225 |
| O | 16.825 | 4.394 | -3.038 |
| H | 15.944 | 3.938 | -6.009 |
| H | 14.709 | 2.963 | -3.561 |
| O | 16.847 | 1.270 | -5.360 |
| H | 18.277 | 0.168 | -3.541 |
| H | 18.037 | -0.907 | -1.092 |
| H | 18.341 | 0.841 | -1.214 |
| H | 15.651 | -0.557 | -0.870 |
| H | 16.251 | 0.779 | 0.103 |
| H | 14.594 | 1.178 | -2.064 |
| H | 15.843 | 2.372 | -1.644 |
| H | 10.368 | 5.083 | -0.356 |
| O | 10.428 | 5.958 | -3.498 |
| C | 11.613 | 2.675 | -0.768 |
| C | 12.952 | 4.734 | -1.306 |
| H | 11.693 | 3.666 | -2.672 |
| C | 15.055 | 10.268 | -8.373 |
| C | 17.486 | 10.051 | -7.858 |
| H | 15.934 | 10.221 | -6.404 |
| C | 13.077 | 3.425 | -5.737 |
| C | 13.418 | 0.993 | -5.139 |
| H | 14.617 | 2.126 | -6.472 |
| O | 17.443 | -2.406 | -2.925 |
| O | 15.871 | -1.423 | -4.197 |
| H | -0.330 | 0.699 | -0.732 |
| H | -0.396 | -0.899 | -0.236 |
| H | -0.326 | 0.320 | 0.905 |
| H | 5.987 | 2.839 | 2.928 |
| H | 4.893 | 1.539 | 2.450 |
| H | 6.215 | 2.042 | 1.393 |
| H | 3.741 | 3.946 | 3.393 |
| H | 2.614 | 4.324 | 2.087 |
| H | 2.858 | 2.649 | 2.594 |
| H | 10.664 | 2.150 | -0.864 |
| H | 12.409 | 1.990 | -1.059 |
| H | 11.761 | 2.901 | 0.291 |
| H | 13.835 | 4.107 | -1.427 |
| H | 13.073 | 5.603 | -1.950 |
| H | 12.958 | 5.079 | -0.268 |
| H | 14.029 | 10.016 | -8.110 |
| H | 15.135 | 11.353 | -8.444 |
| H | 15.246 | 9.861 | -9.370 |
| H | 18.231 | 9.914 | -7.079 |
| H | 17.749 | 9.428 | -8.718 |
| H | 17.533 | 11.092 | -8.181 |
| H | 12.373 | 3.162 | -6.525 |
| H | 12.498 | 3.606 | -4.828 |
| H | 13.557 | 4.366 | -6.007 |
| H | 12.934 | 1.082 | -4.163 |
| H | 12.652 | 0.723 | -5.867 |
| H | 14.143 | 0.180 | -5.090 |
| O | 4.724 | 7.377 | -1.559 |
| H | 5.089 | 7.659 | -0.707 |
| H | 3.806 | 7.127 | -1.448 |
| O | 15.833 | 4.419 | -8.063 |
| H | 15.484 | 5.066 | -8.676 |
| H | 16.345 | 3.762 | -8.541 |
| O | 7.370 | -1.047 | -0.043 |
| H | 7.609 | -0.564 | -0.862 |
| H | 8.019 | -0.845 | 0.652 |
| O | 8.408 | 2.193 | 3.328 |
| H | 8.559 | 1.518 | 2.660 |
| H | 8.328 | 3.049 | 2.894 |
| O | 10.492 | 10.489 | -5.626 |
| H | 9.527 | 10.428 | -5.524 |
| H | 10.812 | 11.233 | -5.117 |
| O | 19.383 | 2.018 | -5.607 |
| H | 19.451 | 2.199 | -6.544 |
| H | 18.472 | 1.724 | -5.404 |
| O | 9.623 | 1.605 | -3.623 |
| H | 9.973 | 2.056 | -4.407 |
| H | 10.207 | 0.861 | -3.372 |
| O | 8.350 | 6.996 | -4.907 |
| H | 8.498 | 7.563 | -5.663 |
| H | 9.203 | 6.626 | -4.605 |
| O | 10.326 | 5.980 | -7.090 |
| H | 9.646 | 6.649 | -7.096 |
| H | 11.102 | 6.308 | -6.610 |
| O | 6.150 | 7.455 | 0.842 |
| H | 6.745 | 6.738 | 0.586 |
| H | 6.631 | 8.096 | 1.367 |
| O | 12.510 | -0.433 | 1.949 |
| H | 12.149 | -0.498 | 1.049 |
| H | 12.128 | -1.112 | 2.504 |
| O | 1.536 | 4.504 | -1.829 |
| H | 0.945 | 5.141 | -1.430 |
| H | 1.569 | 3.704 | -1.292 |
| O | 20.540 | 7.394 | -6.894 |
| H | 20.557 | 7.972 | -6.097 |
| H | 20.863 | 7.891 | -7.653 |
| O | 8.008 | 10.678 | -4.515 |
| H | 8.242 | 11.261 | -3.794 |
| H | 7.603 | 9.862 | -4.155 |
| O | 21.383 | 4.676 | -7.698 |
| H | 22.014 | 4.508 | -6.998 |
| H | 20.975 | 5.538 | -7.570 |
| O | 18.292 | 10.033 | -3.343 |
| H | 17.801 | 9.664 | -4.086 |
| H | 18.285 | 9.414 | -2.611 |
| O | 9.922 | -3.135 | 0.209 |
| H | 9.114 | -3.594 | 0.489 |
| H | 10.684 | -3.671 | 0.439 |
| O | 18.944 | 6.525 | -8.927 |
| H | 18.952 | 7.241 | -9.561 |
| H | 19.300 | 6.838 | -8.077 |
| O | 9.059 | -0.216 | 1.769 |
| H | 9.925 | -0.429 | 1.389 |
| H | 9.090 | -0.355 | 2.718 |
| O | 24.409 | 6.364 | -8.140 |
| H | 25.057 | 6.969 | -8.501 |
| H | 23.535 | 6.587 | -8.463 |
| O | 8.614 | 4.653 | 1.593 |
| H | 8.494 | 5.202 | 0.802 |
| H | 9.548 | 4.474 | 1.720 |
| O | 21.074 | 12.103 | -8.499 |
| H | 20.253 | 12.295 | -8.952 |
| H | 21.423 | 11.267 | -8.820 |
| O | 13.522 | -1.578 | -3.169 |
| H | 13.641 | -1.958 | -2.297 |
| H | 14.418 | -1.374 | -3.562 |
| O | 6.584 | 8.417 | -3.560 |
| H | 6.303 | 8.089 | -2.702 |
| H | 7.153 | 7.764 | -4.004 |
| O | 21.291 | 3.003 | -3.903 |
| H | 20.561 | 2.551 | -4.354 |
| H | 21.792 | 3.512 | -4.536 |
| O | 4.719 | -0.892 | 0.514 |
| H | 4.666 | -1.031 | 1.472 |
| H | 5.669 | -0.905 | 0.229 |
| O | 16.465 | 4.562 | -0.315 |
| H | 16.749 | 4.554 | -1.244 |
| H | 16.916 | 3.871 | 0.168 |
| O | 14.490 | 12.009 | -3.519 |
| H | 14.768 | 12.592 | -4.226 |
| H | 13.544 | 11.874 | -3.562 |
| O | 9.756 | 3.211 | -6.225 |
| H | 8.869 | 2.940 | -6.460 |
| H | 9.874 | 4.154 | -6.432 |
| O | 15.763 | 7.020 | -1.389 |
| H | 14.846 | 7.253 | -1.566 |
| H | 15.793 | 6.215 | -0.862 |
| O | 0.197 | -0.107 | 2.735 |
| H | -0.261 | -0.759 | 3.271 |
| H | 0.768 | 0.430 | 3.289 |
| O | 10.890 | -0.691 | -3.121 |
| H | 11.864 | -0.871 | -3.234 |
| H | 10.395 | -1.467 | -3.390 |
| O | 7.213 | -4.016 | 0.130 |
| H | 6.973 | -4.524 | -0.645 |
| H | 7.121 | -3.068 | -0.059 |
| O | 7.628 | 0.141 | -2.464 |
| H | 8.379 | 0.466 | -2.979 |
| H | 6.862 | 0.706 | -2.605 |
| O | 4.168 | -1.129 | 3.232 |
| H | 4.926 | -1.383 | 3.762 |
| H | 3.454 | -1.754 | 3.364 |
| O | 4.004 | -3.177 | -0.671 |
| H | 3.048 | -3.161 | -0.676 |
| H | 4.335 | -2.328 | -0.334 |
| O | 11.369 | -0.910 | -0.403 |
| H | 11.299 | -0.625 | -1.325 |
| H | 10.775 | -1.663 | -0.255 |
| O | 21.814 | 9.258 | -8.725 |
| H | 21.498 | 9.154 | -9.626 |
| H | 22.769 | 9.160 | -8.701 |
| O | -0.287 | 3.086 | 1.976 |
| H | -0.086 | 2.807 | 2.869 |
| H | -1.210 | 3.338 | 1.921 |
| O | 20.720 | 8.952 | -4.796 |
| H | 19.855 | 9.141 | -4.391 |
| H | 20.997 | 9.703 | -5.324 |
|  |  |  |  |
| B1-B2 |  |  |  |
| N | 0.001 | 0.060 | -0.012 |
| C | 1.496 | 0.051 | -0.016 |
| C | 1.946 | 1.503 | -0.013 |
| H | 1.851 | -0.504 | 0.849 |
| H | 1.822 | -0.446 | -0.929 |
| N | 3.211 | 1.704 | 0.284 |
| O | 1.076 | 2.325 | -0.278 |
| C | 3.942 | 2.951 | 0.127 |
| H | 3.816 | 0.896 | 0.345 |
| C | 4.989 | 2.687 | -0.980 |
| C | 4.491 | 3.472 | 1.470 |
| H | 3.232 | 3.682 | -0.264 |
| C | 5.429 | 2.450 | 2.119 |
| C | 3.330 | 3.647 | 2.460 |
| H | 4.989 | 4.418 | 1.275 |
| N | 5.665 | 3.757 | -1.393 |
| O | 5.135 | 1.546 | -1.405 |
| C | 6.845 | 3.699 | -2.231 |
| H | 5.473 | 4.650 | -0.951 |
| C | 8.011 | 4.446 | -1.612 |
| H | 7.095 | 2.654 | -2.391 |
| H | 6.645 | 4.172 | -3.198 |
| N | 9.218 | 3.928 | -1.798 |
| O | 7.805 | 5.455 | -0.907 |
| C | 10.426 | 4.680 | -1.447 |
| H | 9.318 | 3.262 | -2.561 |
| C | 10.474 | 5.925 | -2.342 |
| N | 10.637 | 7.129 | -1.764 |
| C | 10.914 | 8.322 | -2.546 |
| C | 10.570 | 7.389 | -0.319 |
| C | 10.663 | 9.488 | -1.566 |
| C | 12.365 | 8.395 | -3.014 |
| C | 10.836 | 8.889 | -0.171 |
| N | 12.573 | 8.955 | -4.213 |
| C | 13.788 | 8.748 | -4.953 |
| C | 13.581 | 7.835 | -6.147 |
| N | 14.560 | 7.772 | -7.095 |
| C | 15.926 | 8.293 | -7.120 |
| C | 16.774 | 7.678 | -5.983 |
| N | 16.886 | 6.361 | -5.962 |
| C | 17.468 | 5.625 | -4.858 |
| C | 16.528 | 4.578 | -4.276 |
| N | 15.792 | 3.866 | -5.158 |
| C | 15.076 | 2.691 | -4.691 |
| C | 16.152 | 1.620 | -4.467 |
| N | 16.337 | 1.101 | -3.274 |
| C | 17.375 | 0.101 | -3.007 |
| C | 15.549 | 1.385 | -2.064 |
| C | 17.570 | 0.211 | -1.503 |
| C | 16.148 | 0.457 | -1.003 |
| C | 16.032 | 9.831 | -7.244 |
| C | 14.041 | 2.243 | -5.726 |
| C | 16.908 | -1.323 | -3.473 |
| C | 11.668 | 3.790 | -1.623 |
| H | 10.248 | 8.359 | -3.407 |
| H | 11.340 | 10.321 | -1.746 |
| H | 9.644 | 9.850 | -1.707 |
| H | 11.858 | 9.052 | 0.171 |
| H | 10.173 | 9.346 | 0.561 |
| H | 11.319 | 6.806 | 0.215 |
| H | 9.586 | 7.101 | 0.053 |
| O | 13.270 | 7.948 | -2.319 |
| H | 11.751 | 9.193 | -4.748 |
| H | 14.166 | 9.711 | -5.293 |
| H | 14.535 | 8.335 | -4.272 |
| O | 12.527 | 7.250 | -6.327 |
| H | 14.251 | 7.246 | -7.895 |
| H | 16.349 | 7.883 | -8.043 |
| O | 17.360 | 8.390 | -5.170 |
| H | 16.518 | 5.825 | -6.730 |
| H | 18.389 | 5.125 | -5.167 |
| H | 17.738 | 6.311 | -4.065 |
| O | 16.441 | 4.393 | -3.078 |
| H | 16.115 | 3.813 | -6.112 |
| H | 14.582 | 2.956 | -3.759 |
| O | 16.887 | 1.380 | -5.447 |
| H | 18.271 | 0.339 | -3.578 |
| H | 18.010 | -0.704 | -1.116 |
| H | 18.216 | 1.058 | -1.260 |
| H | 15.600 | -0.484 | -0.971 |
| H | 16.099 | 0.902 | -0.012 |
| H | 14.492 | 1.177 | -2.232 |
| H | 15.681 | 2.434 | -1.797 |
| H | 10.337 | 4.955 | -0.397 |
| O | 10.469 | 5.782 | -3.563 |
| C | 11.573 | 2.546 | -0.748 |
| C | 12.950 | 4.556 | -1.352 |
| H | 11.669 | 3.474 | -2.672 |
| C | 15.053 | 10.358 | -8.292 |
| C | 17.449 | 10.290 | -7.586 |
| H | 15.780 | 10.278 | -6.281 |
| C | 13.010 | 3.340 | -5.973 |
| C | 13.387 | 0.932 | -5.307 |
| H | 14.592 | 2.058 | -6.655 |
| O | 17.513 | -2.270 | -2.982 |
| O | 15.986 | -1.323 | -4.344 |
| H | -0.296 | 0.737 | -0.725 |
| H | -0.396 | -0.858 | -0.200 |
| H | -0.359 | 0.412 | 0.874 |
| H | 5.961 | 2.896 | 2.960 |
| H | 4.890 | 1.587 | 2.505 |
| H | 6.196 | 2.091 | 1.431 |
| H | 3.705 | 3.977 | 3.429 |
| H | 2.592 | 4.378 | 2.121 |
| H | 2.816 | 2.698 | 2.611 |
| H | 10.616 | 2.040 | -0.845 |
| H | 12.348 | 1.831 | -1.016 |
| H | 11.721 | 2.796 | 0.305 |
| H | 13.813 | 3.900 | -1.458 |
| H | 13.090 | 5.395 | -2.033 |
| H | 12.979 | 4.938 | -0.331 |
| H | 14.028 | 10.046 | -8.098 |
| H | 15.085 | 11.447 | -8.307 |
| H | 15.326 | 10.010 | -9.293 |
| H | 18.124 | 10.178 | -6.744 |
| H | 17.861 | 9.751 | -8.443 |
| H | 17.440 | 11.349 | -7.845 |
| H | 12.325 | 3.055 | -6.774 |
| H | 12.424 | 3.519 | -5.066 |
| H | 13.467 | 4.292 | -6.243 |
| H | 12.868 | 1.039 | -4.350 |
| H | 12.658 | 0.614 | -6.055 |
| H | 14.129 | 0.140 | -5.193 |
| O | 13.183 | 0.111 | 1.297 |
| H | 12.663 | -0.208 | 2.034 |
| H | 12.735 | -0.109 | 0.463 |
| O | 9.926 | 10.190 | -5.395 |
| H | 9.795 | 10.330 | -6.335 |
| H | 9.085 | 10.335 | -4.923 |
| O | 8.232 | 4.377 | 1.684 |
| H | 7.484 | 4.474 | 2.273 |
| H | 8.046 | 4.840 | 0.848 |
| O | 16.302 | 4.640 | -8.006 |
| H | 15.500 | 4.935 | -8.443 |
| H | 16.899 | 4.254 | -8.650 |
| O | 19.374 | 6.262 | -7.934 |
| H | 18.788 | 6.369 | -8.681 |
| H | 19.667 | 5.337 | -7.867 |
| O | 20.293 | 8.781 | -6.233 |
| H | 20.704 | 8.242 | -6.913 |
| H | 19.341 | 8.774 | -6.352 |
| O | 9.434 | 6.737 | -6.024 |
| H | 10.023 | 6.285 | -5.406 |
| H | 9.719 | 7.645 | -6.138 |
| O | 6.092 | -0.864 | 0.465 |
| H | 5.444 | -1.123 | -0.189 |
| H | 6.861 | -1.464 | 0.424 |
| O | 2.849 | 0.047 | 3.227 |
| H | 2.680 | 0.440 | 4.087 |
| H | 2.434 | -0.817 | 3.184 |
| O | 9.909 | -0.155 | -1.975 |
| H | 9.270 | 0.290 | -1.396 |
| H | 9.643 | -0.056 | -2.896 |
| O | 12.344 | -0.909 | -1.017 |
| H | 12.942 | -1.104 | -1.763 |
| H | 11.480 | -0.640 | -1.356 |
| O | 4.754 | 6.334 | 0.061 |
| H | 5.118 | 7.019 | 0.620 |
| H | 3.788 | 6.372 | 0.091 |
| O | 13.631 | -1.955 | -3.418 |
| H | 14.509 | -1.731 | -3.832 |
| H | 12.959 | -2.004 | -4.096 |
| O | 9.857 | -1.193 | 1.918 |
| H | 9.689 | -1.740 | 1.153 |
| H | 9.476 | -0.320 | 1.778 |
| O | 18.840 | -0.695 | -6.074 |
| H | 19.261 | 0.133 | -6.288 |
| H | 17.940 | -0.532 | -5.774 |
| O | 7.224 | 8.072 | -2.159 |
| H | 7.602 | 7.448 | -1.520 |
| H | 6.883 | 8.839 | -1.697 |
| O | 20.530 | 3.754 | -7.921 |
| H | 21.363 | 3.308 | -7.770 |
| H | 19.803 | 3.242 | -7.514 |
| O | 9.510 | 3.204 | -4.631 |
| H | 10.189 | 3.884 | -4.712 |
| H | 9.776 | 2.416 | -5.107 |
| O | 7.319 | 10.263 | -4.437 |
| H | 6.748 | 10.038 | -5.174 |
| H | 7.100 | 9.715 | -3.681 |
| O | 7.442 | -3.095 | 0.322 |
| H | 8.309 | -3.493 | 0.218 |
| H | 6.955 | -3.173 | -0.500 |
| O | 22.304 | 8.303 | -8.724 |
| H | 22.081 | 7.564 | -8.150 |
| H | 23.095 | 8.735 | -8.404 |
| O | 2.049 | 6.801 | 0.098 |
| H | 1.581 | 6.955 | 0.923 |
| H | 1.770 | 7.458 | -0.547 |
| O | 12.257 | 11.752 | -5.857 |
| H | 11.431 | 11.273 | -5.736 |
| H | 12.376 | 11.957 | -6.785 |
| O | 4.954 | 6.278 | -3.187 |
| H | 5.394 | 6.337 | -2.338 |
| H | 5.614 | 6.326 | -3.895 |
| O | 21.176 | 3.258 | -4.097 |
| H | 20.971 | 2.371 | -4.401 |
| H | 21.754 | 3.680 | -4.732 |
| O | 8.960 | 1.589 | 2.212 |
| H | 9.375 | 1.494 | 3.071 |
| H | 8.789 | 2.529 | 2.033 |
| O | 16.589 | 10.429 | -3.816 |
| H | 16.839 | 9.648 | -4.333 |
| H | 17.201 | 10.540 | -3.087 |
| O | 21.942 | 6.424 | -6.894 |
| H | 21.073 | 6.021 | -6.911 |
| H | 22.420 | 6.133 | -6.116 |
| O | 20.101 | 5.256 | -2.603 |
| H | 20.008 | 6.053 | -3.138 |
| H | 20.604 | 4.598 | -3.080 |
| O | 7.273 | 6.560 | -4.536 |
| H | 8.002 | 6.715 | -5.172 |
| H | 7.330 | 7.204 | -3.824 |
| O | 7.969 | 0.873 | -0.513 |
| H | 7.100 | 0.479 | -0.358 |
| H | 8.353 | 1.153 | 0.328 |
| O | 0.720 | 4.919 | -0.985 |
| H | 1.258 | 5.543 | -0.498 |
| H | 0.985 | 4.025 | -0.760 |
| O | 10.838 | -3.200 | -2.636 |
| H | 11.146 | -3.578 | -1.812 |
| H | 10.598 | -2.279 | -2.499 |
| O | 8.799 | -0.293 | -4.436 |
| H | 8.179 | 0.301 | -4.862 |
| H | 9.059 | -0.970 | -5.064 |
| O | 19.930 | 7.599 | -3.782 |
| H | 19.231 | 7.924 | -3.212 |
| H | 19.834 | 7.979 | -4.667 |
| O | 14.483 | 6.973 | -0.045 |
| H | 13.997 | 7.219 | -0.842 |
| H | 14.008 | 7.284 | 0.725 |
| O | 17.095 | 5.804 | -0.820 |
| H | 16.907 | 5.197 | -1.543 |
| H | 16.267 | 6.113 | -0.439 |
| O | 14.539 | 10.439 | -1.571 |
| H | 15.005 | 10.633 | -2.393 |
| H | 14.259 | 9.521 | -1.587 |
| O | 5.443 | -0.847 | 3.168 |
| H | 5.903 | -0.735 | 2.332 |
| H | 4.531 | -0.580 | 3.065 |
| O | 18.850 | 1.716 | -7.148 |
| H | 18.162 | 1.721 | -6.440 |
| H | 18.435 | 1.524 | -7.990 |
|  |  |  |  |
| B2-C1 |  |  |  |
| N | 0.052 | -0.008 | -0.020 |
| C | 1.526 | -0.003 | -0.013 |
| C | 2.177 | 1.349 | -0.008 |
| H | 1.862 | -0.604 | 0.835 |
| H | 1.849 | -0.526 | -0.913 |
| N | 3.511 | 1.280 | -0.057 |
| O | 1.533 | 2.393 | -0.051 |
| C | 4.286 | 2.442 | -0.405 |
| H | 3.951 | 0.367 | 0.015 |
| C | 4.070 | 2.878 | -1.864 |
| C | 5.786 | 2.115 | -0.180 |
| H | 3.991 | 3.273 | 0.236 |
| C | 6.302 | 1.008 | -1.103 |
| C | 6.013 | 1.686 | 1.280 |
| H | 6.342 | 3.031 | -0.374 |
| N | 4.424 | 4.145 | -2.073 |
| O | 3.507 | 2.192 | -2.712 |
| C | 4.193 | 4.895 | -3.281 |
| H | 4.850 | 4.683 | -1.321 |
| C | 3.312 | 6.097 | -2.952 |
| H | 5.119 | 5.272 | -3.712 |
| H | 3.707 | 4.270 | -4.020 |
| N | 2.277 | 5.780 | -2.140 |
| O | 3.513 | 7.213 | -3.403 |
| C | 1.560 | 6.760 | -1.357 |
| H | 2.344 | 4.885 | -1.678 |
| C | 2.307 | 6.812 | -0.018 |
| N | 2.543 | 7.986 | 0.568 |
| C | 3.127 | 8.053 | 1.898 |
| C | 2.157 | 9.314 | 0.048 |
| C | 3.603 | 9.502 | 1.993 |
| C | 2.184 | 7.587 | 3.004 |
| C | 2.615 | 10.288 | 1.135 |
| N | 2.724 | 6.821 | 3.947 |
| C | 1.994 | 6.439 | 5.139 |
| C | 2.852 | 6.645 | 6.381 |
| N | 3.110 | 5.640 | 7.236 |
| C | 2.746 | 4.238 | 7.240 |
| C | 3.668 | 3.445 | 6.277 |
| N | 3.106 | 2.459 | 5.560 |
| C | 3.837 | 1.613 | 4.633 |
| C | 3.714 | 0.145 | 5.055 |
| N | 2.458 | -0.313 | 5.038 |
| C | 2.072 | -1.701 | 5.167 |
| C | 2.360 | -2.472 | 3.875 |
| N | 2.873 | -3.693 | 3.995 |
| C | 3.295 | -4.485 | 2.840 |
| C | 3.121 | -4.397 | 5.267 |
| C | 4.190 | -5.568 | 3.447 |
| C | 3.640 | -5.774 | 4.855 |
| C | 1.225 | 3.947 | 7.235 |
| C | 0.547 | -1.696 | 5.455 |
| C | 2.102 | -5.059 | 2.033 |
| C | 0.094 | 6.317 | -1.168 |
| H | 3.976 | 7.368 | 1.928 |
| H | 3.680 | 9.853 | 3.016 |
| H | 4.606 | 9.558 | 1.570 |
| H | 1.765 | 10.607 | 1.737 |
| H | 3.063 | 11.181 | 0.705 |
| H | 1.086 | 9.377 | -0.131 |
| H | 2.667 | 9.499 | -0.897 |
| O | 0.976 | 7.876 | 3.012 |
| H | 3.688 | 6.535 | 3.840 |
| H | 1.137 | 7.101 | 5.232 |
| H | 1.636 | 5.415 | 5.036 |
| O | 3.273 | 7.767 | 6.612 |
| H | 3.739 | 5.941 | 7.964 |
| H | 3.097 | 3.890 | 8.216 |
| O | 4.848 | 3.755 | 6.216 |
| H | 2.098 | 2.369 | 5.546 |
| H | 3.453 | 1.719 | 3.617 |
| H | 4.885 | 1.898 | 4.658 |
| O | 4.692 | -0.572 | 5.255 |
| H | 1.727 | 0.327 | 4.746 |
| H | 2.616 | -2.140 | 6.000 |
| O | 2.173 | -1.911 | 2.786 |
| H | 3.854 | -3.853 | 2.154 |
| H | 4.172 | -6.479 | 2.852 |
| H | 5.219 | -5.206 | 3.487 |
| H | 2.814 | -6.484 | 4.833 |
| H | 4.384 | -6.135 | 5.562 |
| H | 2.213 | -4.475 | 5.859 |
| H | 3.880 | -3.856 | 5.838 |
| H | 1.611 | 7.710 | -1.887 |
| O | 2.631 | 5.742 | 0.506 |
| C | -0.609 | 6.117 | -2.509 |
| C | -0.697 | 7.261 | -0.275 |
| H | 0.158 | 5.347 | -0.662 |
| C | 0.439 | 5.004 | 8.016 |
| C | 0.947 | 2.586 | 7.881 |
| H | 0.853 | 3.928 | 6.206 |
| C | 0.275 | -1.212 | 6.877 |
| C | -0.161 | -3.021 | 5.202 |
| H | 0.135 | -0.965 | 4.750 |
| O | 2.349 | -5.284 | 0.821 |
| O | 1.012 | -5.216 | 2.622 |
| H | -0.192 | -1.003 | -0.229 |
| H | -0.380 | 0.238 | 0.876 |
| H | -0.288 | 0.615 | -0.770 |
| H | 5.722 | 0.088 | -1.018 |
| H | 6.291 | 1.303 | -2.154 |
| H | 7.332 | 0.761 | -0.843 |
| H | 5.986 | 0.600 | 1.377 |
| H | 7.009 | 1.989 | 1.606 |
| H | 5.294 | 2.129 | 1.967 |
| H | -0.956 | 7.071 | -2.911 |
| H | 0.029 | 5.645 | -3.252 |
| H | -1.500 | 5.500 | -2.368 |
| H | -0.865 | 8.220 | -0.768 |
| H | -1.681 | 6.836 | -0.063 |
| H | -0.204 | 7.442 | 0.679 |
| H | 0.668 | 6.025 | 7.718 |
| H | -0.633 | 4.838 | 7.897 |
| H | 0.663 | 4.931 | 9.082 |
| H | 1.279 | 2.589 | 8.920 |
| H | -0.122 | 2.372 | 7.878 |
| H | 1.460 | 1.762 | 7.391 |
| H | 0.497 | -2.003 | 7.597 |
| H | 0.861 | -0.329 | 7.130 |
| H | -0.782 | -0.967 | 7.002 |
| H | 0.182 | -3.795 | 5.887 |
| H | -1.233 | -2.903 | 5.372 |
| H | -0.013 | -3.384 | 4.184 |
| O | 0.438 | 1.815 | 4.593 |
| H | 0.442 | 2.285 | 3.731 |
| H | -0.468 | 1.728 | 4.913 |
| O | -0.505 | 5.477 | 3.363 |
| H | -0.165 | 6.384 | 3.254 |
| H | -1.127 | 5.442 | 4.089 |
| O | -1.735 | 0.067 | 2.185 |
| H | -1.743 | -0.703 | 2.755 |
| H | -2.404 | 0.693 | 2.469 |
| O | 5.783 | 5.889 | -0.399 |
| H | 6.400 | 5.340 | 0.119 |
| H | 5.882 | 6.804 | -0.138 |
| O | 6.177 | 4.266 | 3.751 |
| H | 6.041 | 4.362 | 4.705 |
| H | 5.317 | 4.240 | 3.298 |
| O | 3.089 | 10.761 | -4.863 |
| H | 2.228 | 10.603 | -5.252 |
| H | 3.682 | 10.041 | -5.089 |
| O | 4.471 | 0.445 | 7.919 |
| H | 4.859 | -0.261 | 7.397 |
| H | 3.720 | 0.103 | 8.418 |
| O | 1.560 | 11.501 | -2.556 |
| H | 2.068 | 12.138 | -2.050 |
| H | 2.130 | 11.071 | -3.202 |
| O | 4.238 | -1.647 | 0.981 |
| H | 3.813 | -1.646 | 1.852 |
| H | 5.066 | -2.159 | 1.012 |
| O | 0.146 | -6.347 | 4.994 |
| H | -0.496 | -7.000 | 4.711 |
| H | 0.333 | -5.737 | 4.248 |
| O | 2.302 | -0.189 | 9.570 |
| H | 2.771 | 0.027 | 10.380 |
| H | 2.278 | -1.151 | 9.464 |
| O | -0.198 | 0.023 | 10.307 |
| H | -0.898 | -0.108 | 9.669 |
| H | 0.660 | 0.035 | 9.862 |
| O | 1.786 | 13.111 | 3.688 |
| H | 1.328 | 13.776 | 4.203 |
| H | 1.202 | 12.769 | 2.995 |
| O | 3.243 | 13.764 | -1.804 |
| H | 2.618 | 13.911 | -1.084 |
| H | 3.698 | 14.577 | -2.020 |
| O | 2.395 | -5.575 | 9.042 |
| H | 2.591 | -6.391 | 9.505 |
| H | 1.481 | -5.613 | 8.699 |
| O | 5.095 | -6.057 | -0.340 |
| H | 5.326 | -6.675 | 0.354 |
| H | 4.215 | -5.688 | -0.155 |
| O | 1.336 | 7.486 | -5.250 |
| H | 2.163 | 7.428 | -4.749 |
| H | 1.180 | 8.396 | -5.497 |
| O | 2.760 | -3.212 | -0.804 |
| H | 3.041 | -2.586 | -0.119 |
| H | 2.660 | -4.097 | -0.397 |
| O | 4.005 | -0.034 | -4.042 |
| H | 3.336 | -0.099 | -4.724 |
| H | 3.845 | 0.757 | -3.506 |
| O | 0.086 | -2.763 | -0.362 |
| H | 0.970 | -3.095 | -0.633 |
| H | -0.262 | -3.320 | 0.338 |
| O | 0.570 | 3.231 | 2.324 |
| H | 0.567 | 2.984 | 1.386 |
| H | 0.041 | 4.035 | 2.457 |
| O | -2.484 | 1.962 | 4.948 |
| H | -2.974 | 1.272 | 5.398 |
| H | -2.719 | 2.820 | 5.306 |
| O | 6.118 | -3.505 | 0.563 |
| H | 6.596 | -3.686 | 1.375 |
| H | 5.830 | -4.349 | 0.168 |
| O | 3.635 | 6.074 | -6.541 |
| H | 3.034 | 5.335 | -6.395 |
| H | 3.126 | 6.823 | -6.853 |
| O | 4.419 | 11.897 | 4.112 |
| H | 4.250 | 11.045 | 4.524 |
| H | 3.617 | 12.215 | 3.689 |
| O | -0.739 | 1.675 | -1.948 |
| H | -0.380 | 1.882 | -2.813 |
| H | -1.412 | 2.320 | -1.724 |
| O | 6.890 | -1.738 | 4.061 |
| H | 7.478 | -1.223 | 3.508 |
| H | 6.191 | -1.162 | 4.413 |
| O | 6.356 | -3.295 | 6.673 |
| H | 6.600 | -2.489 | 6.210 |
| H | 6.823 | -4.038 | 6.289 |
| O | 5.073 | 8.977 | 4.970 |
| H | 4.350 | 8.462 | 5.345 |
| H | 5.621 | 8.388 | 4.445 |
| O | 5.000 | -5.521 | 8.060 |
| H | 4.038 | -5.560 | 8.019 |
| H | 5.296 | -4.655 | 7.761 |
| O | 3.375 | 4.255 | 2.715 |
| H | 2.606 | 3.754 | 3.001 |
| H | 3.190 | 4.665 | 1.854 |
| O | 0.016 | -6.069 | 7.718 |
| H | 0.033 | -6.142 | 6.740 |
| H | -0.367 | -6.864 | 8.089 |
| O | 2.771 | -3.031 | 10.590 |
| H | 2.694 | -3.883 | 10.130 |
| H | 3.126 | -3.177 | 11.466 |
| O | 4.193 | -2.779 | -3.473 |
| H | 4.028 | -3.153 | -2.603 |
| H | 4.193 | -1.815 | -3.432 |
| O | -0.872 | 9.603 | 2.198 |
| H | -0.035 | 9.141 | 2.378 |
| H | -1.584 | 9.129 | 2.624 |
| O | 2.919 | 3.468 | -6.184 |
| H | 3.583 | 2.976 | -6.672 |
| H | 2.882 | 3.119 | -5.289 |
| O | 6.042 | 6.952 | 3.514 |
| H | 6.357 | 7.299 | 2.677 |
| H | 6.466 | 6.095 | 3.675 |
| O | 7.462 | 4.699 | 1.346 |
| H | 7.094 | 4.374 | 2.184 |
| H | 8.284 | 4.240 | 1.165 |
| O | 1.806 | 13.976 | 0.494 |
| H | 1.493 | 13.657 | 1.341 |
| H | 1.533 | 14.888 | 0.382 |
| O | -0.340 | 12.149 | 1.823 |
| H | -0.850 | 12.659 | 2.453 |
| H | -0.621 | 11.215 | 1.867 |
|  |  |  |  |
| B2-C2 |  |  |  |
| N | -0.018 | 0.005 | 0.007 |
| C | 1.470 | 0.001 | 0.019 |
| C | 2.190 | 1.325 | 0.037 |
| H | 1.773 | -0.604 | 0.877 |
| H | 1.785 | -0.533 | -0.878 |
| N | 3.526 | 1.214 | -0.009 |
| O | 1.586 | 2.394 | -0.057 |
| C | 4.333 | 2.358 | -0.344 |
| H | 3.950 | 0.292 | 0.138 |
| C | 4.111 | 2.779 | -1.778 |
| C | 5.841 | 2.065 | -0.107 |
| H | 4.042 | 3.200 | 0.289 |
| C | 6.372 | 0.962 | -1.027 |
| C | 6.086 | 1.650 | 1.352 |
| H | 6.378 | 2.989 | -0.323 |
| N | 4.475 | 4.030 | -2.046 |
| O | 3.580 | 2.040 | -2.606 |
| C | 4.215 | 4.720 | -3.287 |
| H | 4.922 | 4.552 | -1.305 |
| C | 3.349 | 5.955 | -3.020 |
| H | 5.134 | 5.051 | -3.767 |
| H | 3.710 | 4.029 | -3.960 |
| N | 2.322 | 5.713 | -2.172 |
| O | 3.567 | 7.027 | -3.556 |
| C | 1.609 | 6.731 | -1.430 |
| H | 2.377 | 4.842 | -1.662 |
| C | 2.358 | 6.872 | -0.098 |
| N | 2.597 | 8.078 | 0.437 |
| C | 3.185 | 8.131 | 1.760 |
| C | 2.134 | 9.383 | -0.042 |
| C | 3.586 | 9.604 | 1.909 |
| C | 2.231 | 7.679 | 2.853 |
| C | 2.545 | 10.355 | 1.073 |
| N | 2.763 | 6.896 | 3.791 |
| C | 2.004 | 6.535 | 4.966 |
| C | 2.928 | 6.753 | 6.158 |
| N | 3.195 | 5.744 | 7.013 |
| C | 2.793 | 4.355 | 7.055 |
| C | 3.637 | 3.510 | 6.067 |
| N | 3.033 | 2.525 | 5.386 |
| C | 3.776 | 1.681 | 4.460 |
| C | 3.660 | 0.225 | 4.916 |
| N | 2.425 | -0.281 | 4.928 |
| C | 2.142 | -1.696 | 5.104 |
| C | 2.473 | -2.461 | 3.822 |
| N | 3.085 | -3.641 | 3.991 |
| C | 3.548 | -4.401 | 2.850 |
| C | 3.328 | -4.334 | 5.265 |
| C | 4.470 | -5.465 | 3.462 |
| C | 3.899 | -5.692 | 4.859 |
| C | 1.264 | 4.110 | 7.120 |
| C | 0.631 | -1.787 | 5.457 |
| C | 2.408 | -5.008 | 2.041 |
| C | 0.146 | 6.297 | -1.201 |
| H | 4.057 | 7.480 | 1.791 |
| H | 3.601 | 9.928 | 2.948 |
| H | 4.592 | 9.727 | 1.513 |
| H | 1.687 | 10.605 | 1.696 |
| H | 2.941 | 11.287 | 0.672 |
| H | 1.058 | 9.389 | -0.204 |
| H | 2.629 | 9.615 | -0.987 |
| O | 1.059 | 8.048 | 2.870 |
| H | 3.679 | 6.489 | 3.644 |
| H | 1.161 | 7.216 | 5.043 |
| H | 1.624 | 5.517 | 4.880 |
| O | 3.425 | 7.852 | 6.322 |
| H | 3.891 | 6.024 | 7.686 |
| H | 3.171 | 4.015 | 8.024 |
| O | 4.816 | 3.810 | 5.905 |
| H | 2.026 | 2.404 | 5.412 |
| H | 3.378 | 1.778 | 3.449 |
| H | 4.822 | 1.965 | 4.486 |
| O | 4.670 | -0.409 | 5.218 |
| H | 1.683 | 0.290 | 4.540 |
| H | 2.743 | -2.061 | 5.933 |
| O | 2.221 | -1.983 | 2.715 |
| H | 4.108 | -3.752 | 2.180 |
| H | 4.508 | -6.368 | 2.855 |
| H | 5.480 | -5.056 | 3.511 |
| H | 3.092 | -6.425 | 4.835 |
| H | 4.640 | -6.041 | 5.574 |
| H | 2.414 | -4.435 | 5.841 |
| H | 4.048 | -3.768 | 5.861 |
| H | 1.651 | 7.653 | -2.010 |
| O | 2.726 | 5.846 | 0.472 |
| C | -0.577 | 6.026 | -2.519 |
| C | -0.635 | 7.276 | -0.332 |
| H | 0.218 | 5.351 | -0.653 |
| C | 0.545 | 5.200 | 7.918 |
| C | 0.962 | 2.768 | 7.796 |
| H | 0.844 | 4.090 | 6.112 |
| C | 0.405 | -1.281 | 6.882 |
| C | -0.009 | -3.159 | 5.279 |
| H | 0.137 | -1.106 | 4.753 |
| O | 2.652 | -5.227 | 0.827 |
| O | 1.327 | -5.292 | 2.585 |
| H | -0.280 | -0.981 | -0.211 |
| H | -0.431 | 0.362 | 0.885 |
| H | -0.381 | 0.637 | -0.716 |
| H | 5.796 | 0.042 | -0.925 |
| H | 6.359 | 1.258 | -2.078 |
| H | 7.401 | 0.727 | -0.756 |
| H | 6.087 | 0.565 | 1.464 |
| H | 7.079 | 1.979 | 1.661 |
| H | 5.370 | 2.098 | 2.040 |
| H | -0.934 | 6.956 | -2.968 |
| H | 0.060 | 5.508 | -3.233 |
| H | -1.454 | 5.403 | -2.338 |
| H | -0.814 | 8.218 | -0.855 |
| H | -1.619 | 6.862 | -0.100 |
| H | -0.131 | 7.490 | 0.611 |
| H | 0.799 | 6.204 | 7.587 |
| H | -0.533 | 5.071 | 7.834 |
| H | 0.820 | 5.140 | 8.974 |
| H | 1.339 | 2.778 | 8.821 |
| H | -0.117 | 2.609 | 7.832 |
| H | 1.413 | 1.912 | 7.299 |
| H | 0.717 | -2.043 | 7.598 |
| H | 0.957 | -0.361 | 7.084 |
| H | -0.659 | -1.095 | 7.052 |
| H | 0.397 | -3.901 | 5.967 |
| H | -1.076 | -3.091 | 5.497 |
| H | 0.083 | -3.529 | 4.259 |
| O | 0.414 | 1.865 | 4.550 |
| H | -0.480 | 1.533 | 4.685 |
| H | 0.397 | 2.593 | 3.903 |
| O | 6.008 | 6.001 | 0.658 |
| H | 5.741 | 5.564 | 1.476 |
| H | 5.439 | 6.760 | 0.492 |
| O | 0.263 | -6.744 | 4.575 |
| H | 0.678 | -6.151 | 3.930 |
| H | 0.366 | -7.651 | 4.288 |
| O | -1.141 | 9.490 | 3.071 |
| H | -2.023 | 9.132 | 2.962 |
| H | -0.505 | 8.753 | 3.074 |
| O | 4.399 | -1.575 | 7.733 |
| H | 4.892 | -1.313 | 6.948 |
| H | 3.905 | -0.813 | 8.059 |
| O | 4.739 | -5.887 | -0.499 |
| H | 3.955 | -5.592 | 0.020 |
| H | 4.842 | -6.834 | -0.406 |
| O | -1.180 | 11.378 | 0.575 |
| H | -0.512 | 11.842 | 0.065 |
| H | -0.749 | 10.797 | 1.208 |
| O | 2.147 | -3.554 | -1.013 |
| H | 2.731 | -3.685 | -1.759 |
| H | 2.387 | -4.178 | -0.291 |
| O | 4.316 | -1.460 | 0.713 |
| H | 3.544 | -1.995 | 0.923 |
| H | 5.124 | -1.952 | 0.909 |
| O | 6.176 | -3.626 | -0.043 |
| H | 6.786 | -3.444 | -0.761 |
| H | 5.745 | -4.492 | -0.210 |
| O | -0.299 | -6.254 | 7.390 |
| H | 0.601 | -6.241 | 7.735 |
| H | -0.294 | -6.561 | 6.476 |
| O | 1.108 | 2.278 | -3.149 |
| H | 1.154 | 2.532 | -4.073 |
| H | 2.013 | 2.221 | -2.777 |
| O | -1.143 | 1.326 | 2.098 |
| H | -1.125 | 2.281 | 2.219 |
| H | -1.833 | 0.951 | 2.644 |
| O | 0.439 | 12.855 | -0.968 |
| H | -0.229 | 13.459 | -1.303 |
| H | 1.232 | 13.353 | -0.757 |
| O | 2.298 | -6.519 | 8.230 |
| H | 2.477 | -5.581 | 8.045 |
| H | 2.334 | -6.679 | 9.173 |
| O | 4.902 | 0.878 | -4.721 |
| H | 5.246 | -0.010 | -4.815 |
| H | 4.340 | 0.920 | -3.945 |
| O | 3.047 | -1.348 | -4.232 |
| H | 2.303 | -0.777 | -4.018 |
| H | 3.239 | -1.892 | -3.468 |
| O | 5.455 | 5.038 | 3.331 |
| H | 5.303 | 4.581 | 4.178 |
| H | 6.268 | 5.546 | 3.392 |
| O | 5.618 | 8.562 | -0.560 |
| H | 5.869 | 8.441 | -1.480 |
| H | 5.695 | 9.488 | -0.332 |
| O | -1.242 | -3.999 | 2.031 |
| H | -1.225 | -3.433 | 1.265 |
| H | -0.399 | -4.469 | 2.088 |
| O | 0.086 | 11.858 | 3.861 |
| H | 0.394 | 11.660 | 4.745 |
| H | -0.388 | 11.103 | 3.495 |
| O | 0.914 | 3.700 | 2.486 |
| H | 1.606 | 3.666 | 1.817 |
| H | 0.118 | 4.082 | 2.104 |
| O | 0.771 | 11.079 | -3.059 |
| H | 0.776 | 11.700 | -2.318 |
| H | -0.092 | 10.673 | -3.142 |
| O | 5.084 | 10.495 | -4.683 |
| H | 4.215 | 10.234 | -5.016 |
| H | 5.023 | 10.725 | -3.758 |
| O | 2.774 | -3.741 | 8.330 |
| H | 2.181 | -3.369 | 8.998 |
| H | 3.352 | -3.043 | 7.986 |
| O | 2.419 | 9.581 | -4.803 |
| H | 2.653 | 8.664 | -4.616 |
| H | 1.854 | 9.919 | -4.096 |
| O | -1.270 | 8.642 | 6.554 |
| H | -0.400 | 8.512 | 6.930 |
| H | -1.196 | 8.928 | 5.639 |
| O | 3.218 | 0.644 | 9.567 |
| H | 3.477 | 1.506 | 9.892 |
| H | 2.322 | 0.440 | 9.860 |
| O | 7.467 | 5.167 | -1.438 |
| H | 7.138 | 5.730 | -2.139 |
| H | 6.904 | 5.256 | -0.659 |
| O | -1.293 | 2.398 | -1.549 |
| H | -1.793 | 2.079 | -2.301 |
| H | -0.375 | 2.562 | -1.814 |
| O | -1.631 | 4.176 | 1.953 |
| H | -2.233 | 3.919 | 1.249 |
| H | -2.001 | 4.901 | 2.458 |
| O | -0.176 | -2.724 | -0.194 |
| H | -0.946 | -3.012 | -0.691 |
| H | 0.641 | -3.063 | -0.643 |
| O | 6.470 | -2.320 | 4.319 |
| H | 5.766 | -1.755 | 4.671 |
| H | 6.829 | -2.847 | 5.034 |
| O | 1.662 | -2.299 | 10.405 |
| H | 1.075 | -1.543 | 10.369 |
| H | 2.525 | -2.029 | 10.723 |
| O | 7.275 | -1.908 | 1.630 |
| H | 7.011 | -2.672 | 1.104 |
| H | 6.998 | -2.039 | 2.547 |
| O | 0.763 | 0.130 | 10.626 |
| H | 0.736 | 0.466 | 11.526 |
| H | -0.012 | 0.424 | 10.140 |
| O | -2.517 | 4.197 | 5.583 |
| H | -2.759 | 4.769 | 4.857 |
| H | -2.375 | 3.302 | 5.273 |
| O | 1.485 | -7.919 | 1.743 |
| H | 2.239 | -8.149 | 2.285 |
| H | 1.300 | -6.971 | 1.816 |
| O | -2.342 | 0.695 | 4.491 |
| H | -2.879 | 0.758 | 5.284 |
| H | -2.458 | -0.174 | 4.094 |
| O | 0.512 | -0.270 | -3.284 |
| H | 0.382 | -0.530 | -4.198 |
| H | 0.553 | 0.702 | -3.212 |

**Table S4.** Atomic coordinates of the (GVGVP)_2_(H_2_O)_40_ clusters extracted at 85°C.

| B1-A1 |  |  |  |
| --- | --- | --- | --- |
| N | -0.005 | -0.040 | 0.049 |
| C | 1.481 | -0.006 | 0.048 |
| C | 2.057 | 1.403 | 0.031 |
| H | 1.841 | -0.542 | 0.924 |
| H | 1.834 | -0.532 | -0.840 |
| N | 3.389 | 1.394 | -0.110 |
| O | 1.355 | 2.396 | 0.124 |
| C | 4.239 | 2.565 | -0.269 |
| H | 3.865 | 0.498 | -0.072 |
| C | 5.085 | 2.334 | -1.550 |
| C | 5.100 | 2.893 | 0.955 |
| H | 3.556 | 3.403 | -0.440 |
| C | 4.286 | 2.767 | 2.242 |
| C | 5.764 | 4.259 | 0.768 |
| H | 5.901 | 2.151 | 0.990 |
| N | 4.411 | 2.223 | -2.709 |
| O | 6.281 | 2.090 | -1.461 |
| C | 5.007 | 1.650 | -3.890 |
| H | 3.448 | 2.516 | -2.739 |
| C | 5.049 | 2.613 | -5.081 |
| H | 6.029 | 1.373 | -3.637 |
| H | 4.483 | 0.738 | -4.179 |
| N | 5.248 | 2.027 | -6.278 |
| O | 4.814 | 3.800 | -4.950 |
| C | 5.309 | 2.760 | -7.538 |
| H | 5.553 | 1.064 | -6.308 |
| C | 5.030 | 1.716 | -8.614 |
| N | 4.103 | 1.995 | -9.556 |
| C | 3.978 | 1.097 | -10.690 |
| C | 3.428 | 3.286 | -9.732 |
| C | 2.944 | 1.776 | -11.615 |
| C | 5.307 | 0.923 | -11.426 |
| C | 2.782 | 3.219 | -11.119 |
| N | 5.377 | -0.190 | -12.150 |
| C | 6.521 | -0.598 | -12.942 |
| C | 6.922 | -2.011 | -12.521 |
| N | 7.450 | -2.112 | -11.294 |
| C | 7.937 | -3.362 | -10.740 |
| C | 6.991 | -3.888 | -9.653 |
| N | 6.238 | -2.950 | -9.038 |
| C | 5.233 | -3.314 | -8.081 |
| C | 3.969 | -2.506 | -8.322 |
| N | 3.224 | -2.278 | -7.233 |
| C | 1.999 | -1.515 | -7.246 |
| C | 1.967 | -0.758 | -5.923 |
| N | 1.310 | 0.408 | -5.886 |
| C | 1.100 | 1.102 | -4.625 |
| C | 0.709 | 1.087 | -7.039 |
| C | 0.558 | 2.466 | -5.056 |
| C | -0.084 | 2.236 | -6.422 |
| C | 9.388 | -3.216 | -10.234 |
| C | 0.712 | -2.353 | -7.452 |
| C | 0.152 | 0.431 | -3.628 |
| C | 6.646 | 3.487 | -7.899 |
| H | 3.655 | 0.109 | -10.361 |
| H | 3.253 | 1.733 | -12.658 |
| H | 2.001 | 1.234 | -11.538 |
| H | 3.307 | 3.907 | -11.779 |
| H | 1.736 | 3.518 | -11.091 |
| H | 4.146 | 4.104 | -9.690 |
| H | 2.690 | 3.432 | -8.941 |
| O | 6.213 | 1.766 | -11.376 |
| H | 4.528 | -0.750 | -12.223 |
| H | 6.273 | -0.614 | -14.000 |
| H | 7.335 | 0.105 | -12.790 |
| O | 6.718 | -2.975 | -13.254 |
| H | 7.790 | -1.245 | -10.878 |
| H | 7.915 | -4.111 | -11.534 |
| O | 6.975 | -5.066 | -9.346 |
| H | 6.083 | -2.080 | -9.525 |
| H | 5.000 | -4.374 | -8.208 |
| H | 5.594 | -3.182 | -7.059 |
| O | 3.685 | -2.089 | -9.437 |
| H | 3.548 | -2.563 | -6.320 |
| H | 2.077 | -0.809 | -8.074 |
| O | 2.541 | -1.215 | -4.936 |
| H | 2.051 | 1.203 | -4.104 |
| H | -0.138 | 2.873 | -4.327 |
| H | 1.372 | 3.179 | -5.144 |
| H | -1.130 | 1.940 | -6.318 |
| H | -0.046 | 3.124 | -7.049 |
| H | 0.073 | 0.418 | -7.617 |
| H | 1.502 | 1.457 | -7.695 |
| H | 4.518 | 3.506 | -7.498 |
| O | 5.624 | 0.639 | -8.601 |
| C | 7.874 | 2.822 | -7.284 |
| C | 6.612 | 4.983 | -7.598 |
| H | 6.721 | 3.387 | -8.987 |
| C | 10.309 | -2.757 | -11.367 |
| C | 9.920 | -4.495 | -9.595 |
| H | 9.369 | -2.431 | -9.469 |
| C | 0.478 | -3.335 | -6.304 |
| C | 0.730 | -3.057 | -8.805 |
| H | -0.122 | -1.647 | -7.467 |
| O | -0.624 | -0.465 | -4.005 |
| O | 0.183 | 0.935 | -2.464 |
| H | -0.293 | 0.462 | -0.822 |
| H | -0.347 | -0.993 | -0.021 |
| H | -0.361 | 0.397 | 0.909 |
| H | 4.812 | 3.240 | 3.072 |
| H | 3.319 | 3.269 | 2.148 |
| H | 4.104 | 1.725 | 2.502 |
| H | 6.601 | 4.197 | 0.075 |
| H | 5.061 | 5.009 | 0.396 |
| H | 6.164 | 4.615 | 1.718 |
| H | 7.759 | 1.744 | -7.198 |
| H | 8.764 | 3.043 | -7.874 |
| H | 8.055 | 3.193 | -6.275 |
| H | 5.685 | 5.449 | -7.932 |
| H | 6.707 | 5.184 | -6.532 |
| H | 7.443 | 5.480 | -8.102 |
| H | 10.605 | -3.609 | -11.984 |
| H | 9.858 | -1.996 | -12.003 |
| H | 11.231 | -2.342 | -10.955 |
| H | 9.413 | -4.728 | -8.662 |
| H | 9.800 | -5.349 | -10.263 |
| H | 10.986 | -4.379 | -9.386 |
| H | 1.399 | -3.841 | -6.007 |
| H | 0.117 | -2.812 | -5.418 |
| H | -0.257 | -4.086 | -6.587 |
| H | -0.180 | -3.644 | -8.931 |
| H | 0.803 | -2.350 | -9.630 |
| H | 1.576 | -3.743 | -8.872 |
| O | 8.484 | 0.473 | -10.685 |
| H | 9.243 | 1.021 | -10.461 |
| H | 7.736 | 1.050 | -10.939 |
| O | 3.534 | 6.761 | -5.364 |
| H | 3.764 | 6.564 | -4.455 |
| H | 2.722 | 6.296 | -5.597 |
| O | 2.900 | -1.541 | -12.756 |
| H | 1.993 | -1.246 | -12.842 |
| H | 2.924 | -2.426 | -12.306 |
| O | 10.377 | 0.777 | -13.442 |
| H | 10.560 | 1.707 | -13.572 |
| H | 10.180 | 0.624 | -12.513 |
| O | 6.241 | -1.262 | -5.019 |
| H | 6.697 | -1.022 | -5.849 |
| H | 6.889 | -1.288 | -4.304 |
| O | 5.088 | -0.377 | -16.333 |
| H | 5.203 | -1.083 | -15.692 |
| H | 4.154 | -0.193 | -16.425 |
| O | -1.203 | -3.132 | -3.086 |
| H | -1.377 | -3.554 | -3.927 |
| H | -0.912 | -2.226 | -3.244 |
| O | 7.912 | -0.760 | -7.166 |
| H | 7.645 | -0.423 | -8.027 |
| H | 8.825 | -0.533 | -7.002 |
| O | 3.795 | -5.898 | -5.841 |
| H | 4.306 | -6.182 | -6.599 |
| H | 4.194 | -5.105 | -5.458 |
| O | 6.469 | 5.952 | -4.149 |
| H | 6.018 | 6.415 | -3.443 |
| H | 5.870 | 5.304 | -4.538 |
| O | 4.100 | -2.847 | -14.798 |
| H | 3.522 | -3.559 | -15.071 |
| H | 3.761 | -2.467 | -13.980 |
| O | -1.639 | 2.848 | -1.798 |
| H | -2.093 | 3.172 | -2.577 |
| H | -0.934 | 2.221 | -2.061 |
| O | 3.462 | -1.400 | -2.351 |
| H | 3.540 | -1.223 | -3.299 |
| H | 3.115 | -2.288 | -2.227 |
| O | 0.122 | 5.404 | -3.017 |
| H | 0.571 | 5.939 | -2.343 |
| H | -0.311 | 4.656 | -2.596 |
| O | -0.247 | 0.661 | 2.614 |
| H | -0.949 | 1.091 | 3.109 |
| H | 0.392 | 0.289 | 3.223 |
| O | 5.171 | -3.841 | -4.337 |
| H | 5.341 | -3.843 | -3.397 |
| H | 5.437 | -2.987 | -4.718 |
| O | 5.247 | -5.435 | -13.476 |
| H | 5.930 | -4.758 | -13.364 |
| H | 4.601 | -5.126 | -14.110 |
| O | 3.631 | 7.088 | -1.929 |
| H | 2.897 | 6.824 | -1.376 |
| H | 3.887 | 7.982 | -1.700 |
| O | -2.223 | 0.182 | -9.147 |
| H | -1.766 | 1.019 | -9.127 |
| H | -2.420 | -0.098 | -8.240 |
| O | -3.177 | 0.089 | -3.042 |
| H | -3.430 | 0.917 | -3.448 |
| H | -2.216 | -0.011 | -3.101 |
| O | -1.088 | 3.480 | 0.983 |
| H | -1.365 | 3.486 | 0.056 |
| H | -0.178 | 3.168 | 1.053 |
| O | 2.257 | -3.050 | 0.209 |
| H | 2.688 | -3.262 | 1.040 |
| H | 1.397 | -3.479 | 0.178 |
| O | 8.221 | -0.683 | -2.729 |
| H | 7.886 | -0.134 | -2.015 |
| H | 9.024 | -1.125 | -2.449 |
| O | 2.261 | 5.741 | 1.434 |
| H | 3.109 | 5.820 | 1.871 |
| H | 1.585 | 5.470 | 2.073 |
| O | 9.596 | 1.060 | -5.055 |
| H | 9.768 | 0.682 | -5.917 |
| H | 9.059 | 0.452 | -4.539 |
| O | 8.783 | -3.691 | -14.974 |
| H | 8.141 | -3.268 | -14.387 |
| H | 9.664 | -3.401 | -14.741 |
| O | 10.506 | -0.649 | -15.752 |
| H | 10.356 | -0.094 | -14.979 |
| H | 10.506 | -1.564 | -15.480 |
| O | 9.216 | 2.227 | -1.239 |
| H | 9.318 | 1.433 | -0.717 |
| H | 8.292 | 2.321 | -1.500 |
| O | 10.444 | -0.031 | -7.454 |
| H | 10.936 | -0.855 | -7.454 |
| H | 10.855 | 0.588 | -8.060 |
| O | 3.879 | -4.320 | -1.323 |
| H | 3.309 | -3.764 | -0.783 |
| H | 4.789 | -4.047 | -1.205 |
| O | 3.127 | -3.971 | -11.729 |
| H | 2.903 | -4.185 | -10.819 |
| H | 3.969 | -4.387 | -11.965 |
| O | -2.433 | -0.384 | -6.291 |
| H | -3.228 | -0.702 | -5.857 |
| H | -1.673 | -0.461 | -5.695 |
| O | 5.834 | 4.546 | -11.841 |
| H | 6.012 | 4.857 | -12.729 |
| H | 5.972 | 3.589 | -11.793 |
| O | 5.015 | -1.175 | 0.285 |
| H | 4.752 | -1.326 | -0.639 |
| H | 4.547 | -1.789 | 0.848 |
| O | 11.068 | 1.540 | -10.834 |
| H | 11.865 | 1.080 | -10.563 |
| H | 11.302 | 2.272 | -11.408 |
| O | 0.939 | 5.337 | -5.562 |
| H | 0.686 | 5.393 | -4.621 |
| H | 0.161 | 5.204 | -6.101 |
| O | 2.457 | -6.306 | -9.687 |
| H | 1.954 | -6.098 | -8.898 |
| H | 3.263 | -6.765 | -9.445 |
| O | 7.746 | -6.407 | -14.621 |
| H | 8.137 | -5.528 | -14.703 |
| H | 6.877 | -6.338 | -14.213 |
| O | -0.177 | 5.393 | 2.824 |
| H | -0.648 | 4.655 | 2.404 |
| H | -0.782 | 5.887 | 3.375 |
| O | 0.913 | 7.045 | -1.021 |
| H | 0.277 | 7.690 | -0.710 |
| H | 1.208 | 6.492 | -0.279 |
|  |  |  |  |
| B1-A2 |  |  |  |
| N | 0.055 | -0.029 | 0.009 |
| C | 1.531 | -0.020 | 0.004 |
| C | 2.083 | 1.389 | -0.006 |
| H | 1.913 | -0.565 | 0.867 |
| H | 1.858 | -0.555 | -0.887 |
| N | 3.413 | 1.413 | -0.149 |
| O | 1.359 | 2.362 | 0.125 |
| C | 4.213 | 2.618 | -0.302 |
| H | 3.896 | 0.518 | -0.153 |
| C | 5.032 | 2.392 | -1.576 |
| C | 5.057 | 2.983 | 0.924 |
| H | 3.496 | 3.425 | -0.481 |
| C | 4.244 | 2.819 | 2.210 |
| C | 5.665 | 4.374 | 0.748 |
| H | 5.886 | 2.270 | 0.962 |
| N | 4.342 | 2.248 | -2.725 |
| O | 6.241 | 2.235 | -1.523 |
| C | 4.968 | 1.669 | -3.883 |
| H | 3.338 | 2.319 | -2.698 |
| C | 4.993 | 2.595 | -5.088 |
| H | 5.994 | 1.439 | -3.597 |
| H | 4.467 | 0.735 | -4.151 |
| N | 5.222 | 1.991 | -6.258 |
| O | 4.781 | 3.797 | -4.989 |
| C | 5.268 | 2.721 | -7.515 |
| H | 5.568 | 1.033 | -6.252 |
| C | 5.037 | 1.681 | -8.597 |
| N | 4.114 | 1.950 | -9.533 |
| C | 4.011 | 1.076 | -10.684 |
| C | 3.381 | 3.215 | -9.681 |
| C | 2.942 | 1.720 | -11.591 |
| C | 5.351 | 0.966 | -11.392 |
| C | 2.725 | 3.145 | -11.063 |
| N | 5.476 | -0.123 | -12.145 |
| C | 6.647 | -0.469 | -12.920 |
| C | 7.046 | -1.881 | -12.493 |
| N | 7.532 | -1.950 | -11.234 |
| C | 8.023 | -3.166 | -10.620 |
| C | 7.056 | -3.696 | -9.549 |
| N | 6.244 | -2.775 | -8.978 |
| C | 5.216 | -3.138 | -8.043 |
| C | 3.910 | -2.404 | -8.303 |
| N | 3.167 | -2.209 | -7.207 |
| C | 1.895 | -1.518 | -7.195 |
| C | 1.820 | -0.746 | -5.876 |
| N | 1.079 | 0.371 | -5.870 |
| C | 0.807 | 1.093 | -4.638 |
| C | 0.422 | 0.949 | -7.046 |
| C | 0.143 | 2.394 | -5.125 |
| C | -0.472 | 2.048 | -6.479 |
| C | 9.457 | -2.990 | -10.071 |
| C | 0.693 | -2.498 | -7.315 |
| C | -0.077 | 0.364 | -3.612 |
| C | 6.572 | 3.521 | -7.822 |
| H | 3.722 | 0.081 | -10.345 |
| H | 3.257 | 1.726 | -12.632 |
| H | 2.026 | 1.137 | -11.522 |
| H | 3.192 | 3.867 | -11.732 |
| H | 1.668 | 3.395 | -11.004 |
| H | 4.061 | 4.062 | -9.609 |
| H | 2.634 | 3.298 | -8.890 |
| O | 6.213 | 1.839 | -11.283 |
| H | 4.667 | -0.723 | -12.266 |
| H | 6.415 | -0.473 | -13.982 |
| H | 7.431 | 0.257 | -12.710 |
| O | 6.857 | -2.848 | -13.210 |
| H | 7.803 | -1.081 | -10.790 |
| H | 8.029 | -3.935 | -11.394 |
| O | 7.055 | -4.872 | -9.239 |
| H | 6.153 | -1.873 | -9.417 |
| H | 5.049 | -4.214 | -8.128 |
| H | 5.533 | -2.955 | -7.015 |
| O | 3.603 | -1.997 | -9.422 |
| H | 3.512 | -2.503 | -6.304 |
| H | 1.886 | -0.840 | -8.046 |
| O | 2.422 | -1.142 | -4.879 |
| H | 1.746 | 1.296 | -4.124 |
| H | -0.579 | 2.793 | -4.422 |
| H | 0.904 | 3.165 | -5.243 |
| H | -1.488 | 1.671 | -6.351 |
| H | -0.523 | 2.914 | -7.135 |
| H | -0.159 | 0.190 | -7.564 |
| H | 1.166 | 1.339 | -7.742 |
| H | 4.439 | 3.424 | -7.484 |
| O | 5.705 | 0.637 | -8.620 |
| C | 7.813 | 2.922 | -7.160 |
| C | 6.445 | 5.013 | -7.530 |
| H | 6.702 | 3.425 | -8.906 |
| C | 10.422 | -2.563 | -11.176 |
| C | 9.968 | -4.240 | -9.361 |
| H | 9.404 | -2.172 | -9.345 |
| C | 0.608 | -3.448 | -6.119 |
| C | 0.743 | -3.263 | -8.635 |
| H | -0.219 | -1.897 | -7.305 |
| O | -0.762 | -0.614 | -3.939 |
| O | -0.049 | 0.903 | -2.459 |
| H | -0.239 | 0.488 | -0.868 |
| H | -0.290 | -0.981 | -0.080 |
| H | -0.349 | 0.408 | 0.847 |
| H | 4.746 | 3.314 | 3.041 |
| H | 3.255 | 3.275 | 2.117 |
| H | 4.099 | 1.771 | 2.471 |
| H | 6.507 | 4.353 | 0.059 |
| H | 4.932 | 5.100 | 0.388 |
| H | 6.043 | 4.740 | 1.703 |
| H | 7.755 | 1.839 | -7.081 |
| H | 8.709 | 3.176 | -7.726 |
| H | 7.936 | 3.325 | -6.153 |
| H | 5.506 | 5.423 | -7.906 |
| H | 6.496 | 5.208 | -6.462 |
| H | 7.264 | 5.553 | -8.007 |
| H | 10.751 | -3.430 | -11.755 |
| H | 9.999 | -1.818 | -11.848 |
| H | 11.314 | -2.121 | -10.730 |
| H | 9.426 | -4.433 | -8.436 |
| H | 9.850 | -5.127 | -9.985 |
| H | 11.029 | -4.124 | -9.134 |
| H | 1.583 | -3.846 | -5.833 |
| H | 0.200 | -2.930 | -5.252 |
| H | -0.049 | -4.286 | -6.350 |
| H | -0.106 | -3.945 | -8.695 |
| H | 0.720 | -2.601 | -9.501 |
| H | 1.646 | -3.871 | -8.712 |
| O | 4.540 | -1.126 | 0.739 |
| H | 4.738 | -2.065 | 0.698 |
| H | 5.009 | -0.734 | 1.477 |
| O | 6.875 | 3.739 | -13.328 |
| H | 6.488 | 3.182 | -12.646 |
| H | 7.268 | 3.191 | -14.005 |
| O | 3.102 | -3.310 | -3.049 |
| H | 2.484 | -2.678 | -3.423 |
| H | 3.989 | -3.147 | -3.389 |
| O | 4.307 | 5.986 | -3.559 |
| H | 4.526 | 5.074 | -3.815 |
| H | 4.779 | 6.598 | -4.124 |
| O | -0.365 | 3.518 | -1.856 |
| H | 0.207 | 3.777 | -1.129 |
| H | -0.165 | 2.593 | -2.110 |
| O | 2.792 | -1.963 | -12.279 |
| H | 3.070 | -1.650 | -11.411 |
| H | 3.194 | -2.820 | -12.456 |
| O | 6.567 | -2.498 | -1.326 |
| H | 7.248 | -1.895 | -1.636 |
| H | 5.919 | -2.625 | -2.026 |
| O | -3.013 | 3.905 | -2.294 |
| H | -3.558 | 3.206 | -1.932 |
| H | -2.095 | 3.773 | -2.012 |
| O | 5.683 | -2.975 | -4.308 |
| H | 5.710 | -2.139 | -4.795 |
| H | 6.500 | -3.461 | -4.469 |
| O | -3.159 | 1.726 | -4.183 |
| H | -2.879 | 2.641 | -4.273 |
| H | -2.391 | 1.164 | -4.068 |
| O | -0.455 | 6.053 | 0.275 |
| H | -1.327 | 6.099 | -0.124 |
| H | -0.539 | 5.845 | 1.207 |
| O | 8.924 | 1.869 | -10.998 |
| H | 7.958 | 1.985 | -10.957 |
| H | 9.265 | 2.274 | -11.799 |
| O | -0.433 | 5.329 | -3.896 |
| H | 0.379 | 5.632 | -4.315 |
| H | -0.225 | 4.719 | -3.181 |
| O | 8.444 | 0.159 | -8.822 |
| H | 7.470 | 0.272 | -8.771 |
| H | 8.823 | 0.873 | -9.356 |
| O | -0.363 | -3.393 | -2.470 |
| H | -0.610 | -2.648 | -3.033 |
| H | -0.112 | -4.141 | -3.014 |
| O | 3.361 | -4.232 | -13.884 |
| H | 3.316 | -5.095 | -13.474 |
| H | 3.123 | -4.310 | -14.811 |
| O | 7.838 | -1.975 | -17.104 |
| H | 8.584 | -1.468 | -17.431 |
| H | 7.058 | -1.420 | -17.088 |
| O | -2.315 | -1.781 | -5.884 |
| H | -2.581 | -2.623 | -5.516 |
| H | -1.798 | -1.283 | -5.227 |
| O | 7.245 | -5.529 | -14.236 |
| H | 6.676 | -4.916 | -13.766 |
| H | 8.107 | -5.127 | -14.379 |
| O | 4.607 | 1.424 | -14.966 |
| H | 5.233 | 1.237 | -15.660 |
| H | 4.123 | 0.617 | -14.745 |
| O | 8.773 | -1.281 | -6.727 |
| H | 8.865 | -0.650 | -7.493 |
| H | 9.604 | -1.735 | -6.590 |
| O | -1.306 | 0.876 | 2.277 |
| H | -1.212 | 0.501 | 3.156 |
| H | -2.220 | 1.127 | 2.127 |
| O | 8.086 | 5.144 | -4.345 |
| H | 8.666 | 4.986 | -3.598 |
| H | 7.174 | 5.144 | -4.047 |
| O | -0.248 | -1.450 | -11.864 |
| H | -0.710 | -1.356 | -11.031 |
| H | 0.679 | -1.250 | -11.725 |
| O | 8.945 | 2.272 | -1.083 |
| H | 9.221 | 2.011 | -1.968 |
| H | 7.982 | 2.327 | -1.056 |
| O | -0.826 | 3.923 | 3.762 |
| H | -0.600 | 3.234 | 3.132 |
| H | -0.249 | 4.675 | 3.631 |
| O | 8.575 | -0.753 | -3.125 |
| H | 8.866 | -1.665 | -3.190 |
| H | 9.339 | -0.184 | -2.971 |
| O | 6.696 | -0.552 | -5.456 |
| H | 7.406 | -0.983 | -5.946 |
| H | 7.057 | -0.129 | -4.672 |
| O | 10.170 | 1.778 | -3.766 |
| H | 11.111 | 1.922 | -3.666 |
| H | 9.999 | 1.222 | -4.529 |
| O | 8.044 | -3.719 | -5.942 |
| H | 8.171 | -2.830 | -6.286 |
| H | 8.889 | -4.171 | -5.915 |
| O | 11.083 | -1.511 | -14.534 |
| H | 10.435 | -2.203 | -14.343 |
| H | 11.967 | -1.883 | -14.500 |
| O | 11.172 | 0.456 | -6.007 |
| H | 10.798 | -0.382 | -5.733 |
| H | 11.612 | 0.346 | -6.853 |
| O | -0.304 | 1.104 | -10.013 |
| H | -1.108 | 0.792 | -9.594 |
| H | -0.528 | 1.513 | -10.852 |
| O | 3.799 | -1.377 | -15.177 |
| H | 4.582 | -1.874 | -15.441 |
| H | 3.340 | -1.859 | -14.484 |
| O | 1.835 | 7.158 | -0.722 |
| H | 2.659 | 6.702 | -0.555 |
| H | 1.108 | 6.684 | -0.301 |
| O | 5.754 | -3.299 | -16.236 |
| H | 5.779 | -4.260 | -16.247 |
| H | 6.647 | -2.965 | -16.322 |
| O | 1.862 | 6.164 | -5.194 |
| H | 2.182 | 6.421 | -6.056 |
| H | 2.582102 | 6.232138 | -4.54548 |
| O | 9.454 | -3.765 | -15.158 |
| H | 8.923 | -3.083 | -15.578 |
| H | 10.206 | -3.986 | -15.714 |
| O | 10.368 | 1.247 | -14.244 |
| H | 11.000 | 1.697 | -13.681 |
| H | 10.653 | 0.324 | -14.353 |
| O | 11.797 | 1.057 | -10.340 |
| H | 12.427 | 0.713 | -10.974 |
| H | 11.153 | 1.602 | -10.790 |
|  |  |  |  |
| B1-B1 |  |  |  |
| N | -0.066 | -0.019 | -0.057 |
| C | 1.405 | -0.016 | -0.040 |
| C | 1.915 | 1.412 | -0.039 |
| H | 1.757 | -0.524 | 0.856 |
| H | 1.780 | -0.543 | -0.916 |
| N | 3.199 | 1.535 | -0.398 |
| O | 1.207 | 2.321 | 0.370 |
| C | 3.840 | 2.838 | -0.548 |
| H | 3.624 | 0.749 | -0.875 |
| C | 3.887 | 3.071 | -2.069 |
| C | 5.263 | 2.791 | 0.036 |
| H | 3.230 | 3.583 | -0.040 |
| C | 5.216 | 2.855 | 1.559 |
| C | 6.135 | 3.896 | -0.558 |
| H | 5.690 | 1.828 | -0.261 |
| N | 3.052 | 4.019 | -2.547 |
| O | 4.554 | 2.335 | -2.786 |
| C | 2.950 | 4.378 | -3.944 |
| H | 2.561 | 4.597 | -1.882 |
| C | 3.226 | 5.851 | -4.220 |
| H | 3.623 | 3.724 | -4.494 |
| H | 1.934 | 4.183 | -4.299 |
| N | 4.074 | 6.181 | -5.204 |
| O | 2.698 | 6.696 | -3.498 |
| C | 4.293 | 7.580 | -5.552 |
| H | 4.412 | 5.461 | -5.827 |
| C | 3.028 | 8.110 | -6.269 |
| N | 2.550 | 9.330 | -5.982 |
| C | 1.338 | 9.842 | -6.645 |
| C | 3.222 | 10.321 | -5.138 |
| C | 1.133 | 11.240 | -6.044 |
| C | 1.406 | 9.856 | -8.186 |
| C | 2.455 | 11.625 | -5.367 |
| N | 0.592 | 9.000 | -8.842 |
| C | 0.485 | 9.017 | -10.286 |
| C | 1.004 | 7.774 | -11.004 |
| N | 1.944 | 7.082 | -10.349 |
| C | 2.666 | 5.918 | -10.824 |
| C | 2.442 | 4.732 | -9.876 |
| N | 2.010 | 5.034 | -8.640 |
| C | 1.490 | 4.023 | -7.776 |
| C | 0.322 | 4.518 | -6.958 |
| N | -0.648 | 3.627 | -6.733 |
| C | -1.681 | 3.922 | -5.755 |
| C | -1.111 | 3.434 | -4.419 |
| N | -1.282 | 4.206 | -3.336 |
| C | -0.694 | 3.812 | -2.054 |
| C | -1.980 | 5.502 | -3.256 |
| C | -0.534 | 5.153 | -1.347 |
| C | -1.744 | 5.967 | -1.816 |
| C | 4.158 | 6.170 | -11.145 |
| C | -2.931 | 3.059 | -6.065 |
| C | -1.605 | 2.779 | -1.395 |
| C | 5.589 | 7.827 | -6.353 |
| H | 0.516 | 9.181 | -6.362 |
| H | 0.845 | 11.960 | -6.804 |
| H | 0.329 | 11.191 | -5.311 |
| H | 3.030 | 12.273 | -6.027 |
| H | 2.292 | 12.165 | -4.437 |
| H | 4.270 | 10.423 | -5.420 |
| H | 3.182 | 10.005 | -4.093 |
| O | 2.109 | 10.676 | -8.769 |
| H | 0.024 | 8.343 | -8.325 |
| H | -0.557 | 9.127 | -10.585 |
| H | 1.054 | 9.856 | -10.675 |
| O | 0.572 | 7.498 | -12.113 |
| H | 2.249 | 7.496 | -9.479 |
| H | 2.172 | 5.627 | -11.754 |
| O | 2.625 | 3.581 | -10.242 |
| H | 1.963 | 5.978 | -8.288 |
| H | 1.218 | 3.162 | -8.380 |
| H | 2.223 | 3.685 | -7.044 |
| O | 0.289 | 5.668 | -6.515 |
| H | -0.462 | 2.664 | -6.988 |
| H | -1.907 | 4.982 | -5.764 |
| O | -0.498 | 2.371 | -4.395 |
| H | 0.256 | 3.316 | -2.247 |
| H | -0.477 | 5.069 | -0.264 |
| H | 0.385 | 5.623 | -1.702 |
| H | -2.609 | 5.739 | -1.199 |
| H | -1.579 | 7.041 | -1.762 |
| H | -3.040 | 5.391 | -3.475 |
| H | -1.548 | 6.196 | -3.979 |
| H | 4.391 | 8.096 | -4.597 |
| O | 2.494 | 7.414 | -7.124 |
| C | 5.429 | 7.538 | -7.846 |
| C | 6.791 | 7.098 | -5.745 |
| H | 5.777 | 8.900 | -6.258 |
| C | 4.349 | 7.463 | -11.925 |
| C | 4.757 | 4.996 | -11.918 |
| H | 4.692 | 6.262 | -10.194 |
| C | -3.439 | 3.244 | -7.497 |
| C | -4.048 | 3.300 | -5.047 |
| H | -2.609 | 2.021 | -5.941 |
| O | -2.604 | 3.145 | -0.754 |
| O | -1.334 | 1.563 | -1.636 |
| H | -0.469 | 0.357 | 0.814 |
| H | -0.481 | 0.620 | -0.799 |
| H | -0.444 | -0.952 | -0.186 |
| H | 6.224 | 2.816 | 1.971 |
| H | 4.772 | 3.800 | 1.880 |
| H | 4.634 | 2.041 | 1.990 |
| H | 5.574 | 4.826 | -0.656 |
| H | 7.003 | 4.086 | 0.073 |
| H | 6.483 | 3.620 | -1.553 |
| H | 5.001 | 6.548 | -8.020 |
| H | 4.765 | 8.256 | -8.327 |
| H | 6.394 | 7.587 | -8.350 |
| H | 7.170 | 7.632 | -4.872 |
| H | 6.597 | 6.061 | -5.478 |
| H | 7.605 | 7.088 | -6.471 |
| H | 4.067 | 8.349 | -11.359 |
| H | 5.399 | 7.573 | -12.198 |
| H | 3.786 | 7.443 | -12.858 |
| H | 4.592 | 4.046 | -11.417 |
| H | 4.306 | 4.930 | -12.911 |
| H | 5.830 | 5.143 | -12.050 |
| H | -3.316 | 4.268 | -7.843 |
| H | -2.904 | 2.601 | -8.195 |
| H | -4.495 | 2.982 | -7.572 |
| H | -4.834 | 2.556 | -5.176 |
| H | -3.697 | 3.228 | -4.017 |
| H | -4.506 | 4.285 | -5.161 |
| O | 5.019 | 11.576 | -14.925 |
| H | 4.280 | 12.177 | -14.908 |
| H | 5.041 | 11.082 | -14.102 |
| O | -2.680 | 4.980 | 1.341 |
| H | -3.567 | 5.332 | 1.405 |
| H | -2.627 | 4.350 | 0.607 |
| O | 2.862 | 1.762 | -6.057 |
| H | 2.155 | 1.323 | -6.555 |
| H | 3.625 | 1.181 | -6.004 |
| O | 4.347 | -0.583 | -2.317 |
| H | 4.437 | -1.114 | -3.109 |
| H | 4.715 | 0.300 | -2.473 |
| O | 1.557 | -2.208 | -3.350 |
| H | 1.388 | -2.591 | -2.490 |
| H | 1.691 | -1.250 | -3.261 |
| O | 1.501 | 0.641 | -3.376 |
| H | 1.944 | 1.451 | -3.637 |
| H | 0.548 | 0.787 | -3.358 |
| O | 4.421 | 6.699 | 1.014 |
| H | 5.282 | 6.892 | 0.639 |
| H | 3.740 | 7.072 | 0.436 |
| O | -0.822 | 5.384 | 3.411 |
| H | 0.126 | 5.265 | 3.338 |
| H | -1.198 | 5.466 | 2.529 |
| O | -2.388 | 0.864 | 1.184 |
| H | -2.664 | 1.265 | 0.349 |
| H | -2.538 | 1.470 | 1.911 |
| O | 5.483 | 9.286 | -1.608 |
| H | 5.628 | 8.358 | -1.404 |
| H | 6.331 | 9.709 | -1.734 |
| O | -1.221 | 4.944 | -10.172 |
| H | -1.697 | 5.427 | -9.492 |
| H | -1.125 | 5.495 | -10.956 |
| O | 7.369 | 3.432 | -8.672 |
| H | 7.938 | 4.176 | -8.866 |
| H | 6.580 | 3.737 | -8.224 |
| O | -2.115 | -0.120 | -3.565 |
| H | -1.759 | 0.603 | -3.021 |
| H | -3.063 | -0.165 | -3.439 |
| O | -1.289 | 1.473 | -10.137 |
| H | -1.354 | 2.305 | -9.663 |
| H | -1.479 | 0.747 | -9.542 |
| O | -2.598 | 8.690 | -5.278 |
| H | -3.148 | 9.428 | -5.548 |
| H | -2.161 | 8.321 | -6.060 |
| O | 1.958 | 2.791 | 3.241 |
| H | 1.622 | 2.468 | 2.393 |
| H | 1.529 | 2.306 | 3.948 |
| O | -4.159 | 6.375 | -7.118 |
| H | -4.643 | 7.010 | -7.654 |
| H | -4.775 | 5.873 | -6.584 |
| O | -1.961 | 5.914 | -13.459 |
| H | -1.803 | 4.973 | -13.540 |
| H | -1.206 | 6.402 | -13.798 |
| O | 0.596 | 1.090 | -7.294 |
| H | 0.230 | 0.473 | -6.612 |
| H | 1.262 | 0.630 | -7.811 |
| O | 4.497 | 11.156 | -12.315 |
| H | 5.000 | 10.632 | -11.679 |
| H | 4.059 | 11.873 | -11.862 |
| O | 2.183 | 9.140 | -14.391 |
| H | 2.008 | 10.047 | -14.123 |
| H | 2.273 | 8.591 | -13.613 |
| O | 6.228 | 9.995 | -10.768 |
| H | 6.358 | 10.576 | -10.018 |
| H | 7.059 | 9.587 | -11.018 |
| O | 8.492 | 5.696 | -2.534 |
| H | 9.341 | 5.418 | -2.882 |
| H | 8.456 | 6.655 | -2.509 |
| O | -1.656 | 7.064 | -7.603 |
| H | -2.528 | 6.648 | -7.501 |
| H | -0.970 | 6.511 | -7.167 |
| O | -4.996 | 2.048 | -1.504 |
| H | -4.935 | 1.766 | -2.415 |
| H | -4.150 | 2.435 | -1.223 |
| O | 6.911 | 6.994 | -0.477 |
| H | 7.530 | 6.358 | -0.853 |
| H | 7.392 | 7.789 | -0.231 |
| O | 0.095 | 0.050 | 2.816 |
| H | -0.678 | -0.496 | 2.981 |
| H | 0.857 | -0.332 | 3.255 |
| O | 2.429 | 5.309 | 3.212 |
| H | 2.182 | 4.370 | 3.192 |
| H | 3.376 | 5.393 | 3.081 |
| O | 2.570 | 7.570 | -0.788 |
| H | 2.275 | 8.480 | -0.861 |
| H | 2.608 | 7.171 | -1.672 |
| O | -2.877 | 8.916 | -9.502 |
| H | -2.563 | 8.314 | -8.820 |
| H | -2.398 | 9.743 | -9.444 |
| O | 1.364 | 7.134 | 1.604 |
| H | 1.568 | 7.010 | 0.677 |
| H | 1.799 | 6.439 | 2.123 |
| O | 1.145 | 11.506 | -13.447 |
| H | 0.666 | 11.701 | -12.631 |
| H | 1.685 | 12.256 | -13.695 |
| O | 8.392 | 2.471 | -3.346 |
| H | 8.474 | 2.091 | -2.472 |
| H | 8.391 | 3.430 | -3.279 |
| O | 0.380 | 12.240 | -10.887 |
| H | 0.981 | 11.802 | -10.266 |
| H | 0.705 | 13.120 | -11.088 |
| O | 6.970 | 3.610 | -5.648 |
| H | 6.193 | 3.264 | -6.107 |
| H | 7.161 | 3.063 | -4.881 |
| O | -1.987 | 9.815 | -2.761 |
| H | -1.115 | 9.420 | -2.764 |
| H | -2.484 | 9.482 | -3.511 |
| O | -1.244 | 9.841 | -13.343 |
| H | -0.686 | 9.286 | -12.790 |
| H | -2.013 | 9.345 | -13.624 |
| O | -0.030 | -0.846 | -5.536 |
| H | 0.694 | -1.036 | -4.936 |
| H | -0.821 | -0.596 | -5.030 |
| O | 4.782 | 3.223 | -7.250 |
| H | 4.845 | 2.872 | -8.144 |
| H | 3.937 | 2.951 | -6.857 |
| O | -4.053 | 5.436 | -10.960 |
| H | -3.635 | 4.689 | -10.531 |
| H | -3.926 | 5.381 | -11.908 |
|  |  |  |  |
| B1-B2 |  |  |  |
| N | 0.025 | -0.016 | -0.021 |
| C | 1.492 | 0.000 | -0.009 |
| C | 2.000 | 1.427 | -0.006 |
| H | 1.849 | -0.521 | 0.881 |
| H | 1.848 | -0.529 | -0.893 |
| N | 3.282 | 1.576 | -0.366 |
| O | 1.239 | 2.352 | 0.227 |
| C | 3.875 | 2.900 | -0.500 |
| H | 3.855 | 0.779 | -0.635 |
| C | 3.902 | 3.132 | -2.010 |
| C | 5.236 | 2.807 | 0.235 |
| H | 3.218 | 3.617 | -0.010 |
| C | 4.980 | 2.867 | 1.738 |
| C | 6.267 | 3.837 | -0.176 |
| H | 5.649 | 1.826 | -0.011 |
| N | 3.033 | 4.046 | -2.482 |
| O | 4.657 | 2.495 | -2.737 |
| C | 2.929 | 4.381 | -3.883 |
| H | 2.480 | 4.572 | -1.823 |
| C | 3.156 | 5.849 | -4.186 |
| H | 3.635 | 3.743 | -4.413 |
| H | 1.924 | 4.142 | -4.245 |
| N | 4.002 | 6.150 | -5.174 |
| O | 2.587 | 6.707 | -3.507 |
| C | 4.212 | 7.527 | -5.590 |
| H | 4.402 | 5.404 | -5.720 |
| C | 2.963 | 8.034 | -6.341 |
| N | 2.522 | 9.273 | -6.087 |
| C | 1.336 | 9.835 | -6.740 |
| C | 3.216 | 10.237 | -5.231 |
| C | 1.166 | 11.230 | -6.129 |
| C | 1.477 | 9.842 | -8.267 |
| C | 2.494 | 11.569 | -5.443 |
| N | 0.662 | 9.012 | -8.950 |
| C | 0.609 | 9.011 | -10.389 |
| C | 1.110 | 7.739 | -11.074 |
| N | 1.998 | 7.016 | -10.387 |
| C | 2.665 | 5.823 | -10.875 |
| C | 2.395 | 4.605 | -9.984 |
| N | 1.968 | 4.863 | -8.735 |
| C | 1.404 | 3.841 | -7.902 |
| C | 0.258 | 4.396 | -7.084 |
| N | -0.759 | 3.564 | -6.883 |
| C | -1.777 | 3.917 | -5.905 |
| C | -1.213 | 3.366 | -4.590 |
| N | -1.321 | 4.134 | -3.493 |
| C | -0.755 | 3.729 | -2.206 |
| C | -1.973 | 5.452 | -3.423 |
| C | -0.553 | 5.069 | -1.504 |
| C | -1.733 | 5.919 | -1.988 |
| C | 4.160 | 6.037 | -11.214 |
| C | -3.106 | 3.210 | -6.256 |
| C | -1.706 | 2.775 | -1.490 |
| C | 5.511 | 7.701 | -6.411 |
| H | 0.482 | 9.208 | -6.476 |
| H | 0.887 | 11.968 | -6.877 |
| H | 0.366 | 11.179 | -5.393 |
| H | 3.092 | 12.220 | -6.081 |
| H | 2.336 | 12.091 | -4.502 |
| H | 4.262 | 10.297 | -5.523 |
| H | 3.164 | 9.911 | -4.191 |
| O | 2.309 | 10.561 | -8.808 |
| H | -0.025 | 8.459 | -8.446 |
| H | -0.414 | 9.144 | -10.732 |
| H | 1.212 | 9.845 | -10.753 |
| O | 0.691 | 7.431 | -12.182 |
| H | 2.277 | 7.395 | -9.490 |
| H | 2.165 | 5.581 | -11.816 |
| O | 2.489 | 3.472 | -10.428 |
| H | 1.983 | 5.789 | -8.328 |
| H | 1.086 | 3.010 | -8.529 |
| H | 2.137 | 3.434 | -7.194 |
| O | 0.287 | 5.550 | -6.642 |
| H | -0.718 | 2.603 | -7.220 |
| H | -1.894 | 4.996 | -5.893 |
| O | -0.598 | 2.308 | -4.604 |
| H | 0.175 | 3.191 | -2.380 |
| H | -0.499 | 4.973 | -0.422 |
| H | 0.386 | 5.504 | -1.853 |
| H | -2.617 | 5.737 | -1.385 |
| H | -1.533 | 6.990 | -1.974 |
| H | -3.037 | 5.376 | -3.634 |
| H | -1.518 | 6.128 | -4.146 |
| H | 4.332 | 8.093 | -4.667 |
| O | 2.420 | 7.320 | -7.179 |
| C | 5.338 | 7.325 | -7.884 |
| C | 6.698 | 6.990 | -5.757 |
| H | 5.724 | 8.771 | -6.388 |
| C | 4.350 | 7.336 | -11.989 |
| C | 4.729 | 4.858 | -11.999 |
| H | 4.711 | 6.118 | -10.272 |
| C | -3.537 | 3.537 | -7.688 |
| C | -4.214 | 3.559 | -5.264 |
| H | -2.918 | 2.136 | -6.176 |
| O | -2.659 | 3.257 | -0.845 |
| O | -1.529 | 1.536 | -1.583 |
| H | -0.376 | 0.360 | 0.868 |
| H | -0.346 | 0.655 | -0.713 |
| H | -0.340 | -0.937 | -0.278 |
| H | 5.920 | 2.798 | 2.286 |
| H | 4.528 | 3.825 | 2.007 |
| H | 4.318 | 2.077 | 2.081 |
| H | 5.822 | 4.819 | -0.326 |
| H | 7.050 | 3.915 | 0.579 |
| H | 6.732 | 3.545 | -1.114 |
| H | 4.891 | 6.335 | -8.003 |
| H | 4.691 | 8.034 | -8.400 |
| H | 6.300 | 7.322 | -8.392 |
| H | 7.088 | 7.571 | -4.920 |
| H | 6.470 | 5.982 | -5.418 |
| H | 7.505 | 6.903 | -6.484 |
| H | 4.084 | 8.203 | -11.387 |
| H | 5.399 | 7.437 | -12.272 |
| H | 3.745 | 7.337 | -12.899 |
| H | 4.549 | 3.909 | -11.501 |
| H | 4.271 | 4.805 | -12.990 |
| H | 5.803 | 4.988 | -12.133 |
| H | -3.278 | 4.561 | -7.957 |
| H | -3.033 | 2.899 | -8.415 |
| H | -4.611 | 3.394 | -7.809 |
| H | -5.071 | 2.908 | -5.429 |
| H | -3.919 | 3.398 | -4.228 |
| H | -4.541 | 4.596 | -5.378 |
| O | -2.167 | 8.263 | -5.376 |
| H | -1.907 | 7.889 | -6.232 |
| H | -3.098 | 8.477 | -5.390 |
| O | 2.856 | 6.764 | 0.634 |
| H | 2.020 | 7.172 | 0.371 |
| H | 3.472 | 6.810 | -0.109 |
| O | -0.109 | 0.116 | -3.223 |
| H | -0.705 | 0.547 | -2.595 |
| H | 0.187 | 0.755 | -3.875 |
| O | 6.819 | 3.182 | -5.294 |
| H | 7.415 | 3.333 | -4.560 |
| H | 6.465 | 2.293 | -5.232 |
| O | 6.624 | 5.643 | -2.418 |
| H | 7.064 | 4.886 | -2.798 |
| H | 7.265 | 6.209 | -1.977 |
| O | 2.237 | 5.880 | 3.285 |
| H | 2.982 | 6.139 | 2.734 |
| H | 1.575 | 6.569 | 3.266 |
| O | -2.151 | 5.876 | -9.815 |
| H | -1.772 | 5.680 | -10.687 |
| H | -3.032 | 6.234 | -9.932 |
| O | -1.157 | 1.323 | 1.963 |
| H | -0.909 | 1.844 | 2.734 |
| H | -2.092 | 1.487 | 1.747 |
| O | 4.595 | -0.054 | -3.783 |
| H | 4.672 | 0.799 | -3.327 |
| H | 3.687 | -0.355 | -3.739 |
| O | -4.949 | 4.407 | -1.607 |
| H | -4.206 | 3.825 | -1.417 |
| H | -5.260 | 4.803 | -0.793 |
| O | -0.421 | -2.129 | -1.892 |
| H | -0.984 | -2.887 | -2.053 |
| H | -0.597 | -1.441 | -2.560 |
| O | -1.183 | 4.976 | -12.797 |
| H | -1.615 | 5.401 | -13.542 |
| H | -0.293 | 5.328 | -12.711 |
| O | 0.129 | 8.730 | -3.587 |
| H | 0.793 | 8.040 | -3.475 |
| H | -0.592 | 8.398 | -4.142 |
| O | 5.645 | 10.270 | -11.313 |
| H | 6.561 | 10.459 | -11.528 |
| H | 5.162 | 10.079 | -12.119 |
| O | 1.677 | -0.018 | -7.138 |
| H | 2.495 | -0.214 | -7.599 |
| H | 1.461 | -0.760 | -6.569 |
| O | -0.680 | 0.748 | -8.250 |
| H | -0.408 | 0.963 | -9.143 |
| H | 0.057 | 0.333 | -7.783 |
| O | 5.121 | -0.618 | -0.830 |
| H | 4.792 | -1.354 | -1.350 |
| H | 5.136 | -0.874 | 0.097 |
| O | -3.685 | 0.687 | -3.088 |
| H | -4.303 | 0.319 | -2.456 |
| H | -2.925 | 1.041 | -2.615 |
| O | 8.556 | 8.973 | -8.451 |
| H | 9.225 | 8.893 | -7.768 |
| H | 7.854 | 9.556 | -8.132 |
| O | 2.355 | 7.533 | -15.477 |
| H | 3.037 | 8.200 | -15.557 |
| H | 1.490 | 7.950 | -15.480 |
| O | 2.559 | 10.495 | -15.436 |
| H | 3.117 | 10.931 | -14.787 |
| H | 1.641 | 10.617 | -15.206 |
| O | 8.837 | 6.075 | -9.003 |
| H | 9.104 | 6.313 | -9.891 |
| H | 8.590 | 6.865 | -8.516 |
| O | 5.346 | 12.597 | -9.818 |
| H | 6.264 | 12.782 | -9.623 |
| H | 5.262 | 11.718 | -10.229 |
| O | 6.328 | 10.888 | -7.461 |
| H | 6.946 | 11.529 | -7.104 |
| H | 5.585 | 11.352 | -7.868 |
| O | 0.067 | 10.745 | -14.588 |
| H | 0.348 | 11.663 | -14.587 |
| H | -0.572 | 10.602 | -13.888 |
| O | 4.838 | 7.497 | -1.774 |
| H | 5.500 | 6.871 | -2.075 |
| H | 4.014 | 7.339 | -2.252 |
| O | -3.074 | 4.498 | 1.365 |
| H | -2.842 | 4.112 | 0.504 |
| H | -3.388 | 3.804 | 1.947 |
| O | -0.670 | 3.746 | 3.195 |
| H | -0.697 | 4.406 | 3.889 |
| H | -1.097 | 4.090 | 2.397 |
| O | 2.030 | 3.086 | 3.344 |
| H | 1.085 | 2.953 | 3.234 |
| H | 2.274 | 3.943 | 2.983 |
| O | 2.865 | 0.509 | 3.819 |
| H | 2.865 | 1.411 | 3.463 |
| H | 2.523 | 0.516 | 4.712 |
| O | -3.527 | 1.164 | 0.647 |
| H | -3.351 | 1.983 | 0.149 |
| H | -3.117 | 0.416 | 0.205 |
| O | 6.297 | 10.785 | -3.932 |
| H | 5.973 | 11.010 | -4.806 |
| H | 5.720 | 11.170 | -3.271 |
| O | -1.433 | 7.378 | -7.681 |
| H | -0.750 | 6.769 | -7.308 |
| H | -1.877 | 6.932 | -8.423 |
| O | -2.552 | 8.245 | -11.862 |
| H | -3.442 | 7.953 | -11.662 |
| H | -2.379 | 9.074 | -11.414 |
| O | -2.532 | -1.358 | 0.671 |
| H | -3.105 | -1.105 | 1.400 |
| H | -2.309 | -2.286 | 0.760 |
| O | 3.012 | 13.427 | -8.653 |
| H | 2.711 | 12.569 | -8.341 |
| H | 3.766 | 13.311 | -9.239 |
| O | 3.977 | 11.696 | -13.394 |
| H | 4.842 | 11.532 | -13.775 |
| H | 3.845 | 12.643 | -13.311 |
| O | -0.695 | 7.760 | -14.578 |
| H | -0.841 | 8.664 | -14.855 |
| H | -0.382 | 7.750 | -13.662 |
| O | 0.445 | 8.052 | -0.393 |
| H | -0.204 | 8.005 | -1.099 |
| H | 0.417 | 8.924 | 0.003 |
| O | -3.391 | 9.487 | -8.075 |
| H | -2.806 | 8.731 | -8.145 |
| H | -4.153 | 9.255 | -7.544 |

**References**

1. N. V. Ilawe, R. Schweitzer-Stenner, D. DiGuiseppi, B. M. Wong, *Phys. Chem. Chem. Phys.* **2018**, *20*, 18158. [↑](#endnote-ref-2)
2. A. Kumar, S. E. Toal, D. DiGuiseppi, R. Schweitzer-Stenner, B. M. Wong, *J. Phys. Chem. B* **2020**, *124*, 2579. [↑](#endnote-ref-3)
3. N. V. Ilawe, A. E. Raeber, R. Schweitzer-Stenner, S. E. Toal, B. M. Wong, *Phys. Chem. Chem. Phys.* **2015**, *17*, 24917. [↑](#endnote-ref-4)
4. G. Brancato, N. Rega and V. Barone, *J. Chem. Phys*. **2008**, *128*, 144501. [↑](#endnote-ref-5)
